# The role of E3 ubiquitin ligase WWP2 and the regulation of PARP1 by ubiquitinated degradation in acute lymphoblastic leukemia

**DOI:** 10.1038/s41420-022-01209-9

**Published:** 2022-10-18

**Authors:** Xinxin Lu, Xinyue Huang, Haiqi Xu, Saien Lu, Shilong You, Jiaqi Xu, Qianru Zhan, Chao Dong, Ning Zhang, Ying Zhang, Liu Cao, Xingang Zhang, Naijin Zhang, Lijun Zhang

**Affiliations:** 1 Department of Hematology, the First Hospital of China Medical University, Shenyang, Liaoning China; 2 Department of Cardiology, the First Hospital of China Medical University, Shenyang, Liaoning China; 3 Department of Hematology, General Hospital of PLA Northern Theater Command, Shenyang, Liaoning China; 4 Institute of Health Sciences, China Medical University, Shenyang, Liaoning China

**Keywords:** Acute lymphocytic leukaemia, Ubiquitylation, Biomarkers

## Abstract

Acute lymphoblastic leukemia (ALL) has been a huge threat for people's health and finding effective target therapy is urgent and important. WWP2, as one of E3 ubiquitin ligase, is involved in many biological processes by specifically binding to substrates. PARP1 plays a role in cell apoptosis and is considered as a therapeutic target of certain cancers. In this study, we firstly found that WWP2 expressed higher in newly diagnosed ALL patients comparing with complete remission (CR) ALL patients and normal control people, and WWP2 in relapse ALL patients expressed higher than normal control people. WWP2 expression was related with the FAB subtype of ALL and the proportion of blast cells in bone marrow blood tested by flow cytometry. We demonstrated knockout WWP2 inhibited the ALL growth and enhanced apoptosis induced by Dox in vitro and vivo for the first time. WWP2 negatively regulated and interacted with PARP1 and WWP2 mechanically degraded PARP1 through polyubiquitin-proteasome pathway in ALL. These findings suggested WWP2 played a role in ALL development as well as growth and apoptosis, and also displayed a regulatory pathway of PARP1, which provided a new potential therapeutic target for the treatment of ALL.

## Introduction

Acute lymphoblastic leukemia (ALL) is malignant transformation and uncontrolled proliferation of lymphoid hematopoietic progenitor cells, finally invading bone marrow and blood [1, 2]. Although high cure rate can be achieved due to exist therapeutic regimens, subsequent chemotherapy resistance, disease relapse and poor prognosis remain a significant challenge [3]. Therefore, it is urgent and important to study the molecular mechanism of ALL and explore effective target therapy of ALL.

Protein ubiquitination is one of the most important post-translational modifications, leading to protein degradation by the proteasome or lysosome [4, 5]. The ubiquitin-proteasome system is composed of a ubiquitin-activating enzyme (E1), ubiquitin conjugation enzyme (E2), and ubiquitin ligase (E3). E1 activates and transfers ubiquitin to E2, and E3 recruits ubiquitin protein substrates specifically [6]. WWP2 is a HECT-type E3 ubiquitin ligase, one of the major members of the NEDD4 family [7], involved in many biological processes including cell cycle, immune response, apoptosis, and cell signal transduction [8–11]. Studies show WWP2 also participates in the regulation of the proliferation of malignant tumors such as liver cancer, lung cancer and gastric cancer [12–15].

Poly (ADP-ribose) polymerase 1 (PARP1) is the founding member of PARP family [16, 17], which participates in DNA repair and gene integrity [18–20]. And excessive activation of PARP1 induced by oxidative stress leads to the depletion of ATP, which causes cell apoptosis [21]. PARP1 is also involved in transcriptional and posttranscriptional regulation of gene expression. It is reported as a substrate of ubiquitin ligase and plays a role in oxidative-related cardiovascular disease [22]. Due to the crucial roles in many cellular procedures, PARP1 has been considered as a therapeutic target for the potential treatment of cancers [23, 24].

In this study, we firstly tested WWP2 expression in different period ALL patients and normal control people, and analyzed the relationship with clinicopathological factors. We determined that knockout WWP2 significantly inhibit the growth and enhance the apoptosis in ALL xenograft tumors induced by doxorubicin (Dox), as well as at cellular level. We also described WWP2 interacted with PARP1 and mechanically degraded PARP1 through polyubiquitin-proteasome pathway in ALL. The results above showed that WWP2 played a role in multiple effects of ALL, which provided a new potential therapeutic target for ALL.

## Results

### WWP2 expression differences in newly diagnosed ALL patients, CR ALL patients, relapse ALL patients and normal control people

**Fig. 1:**
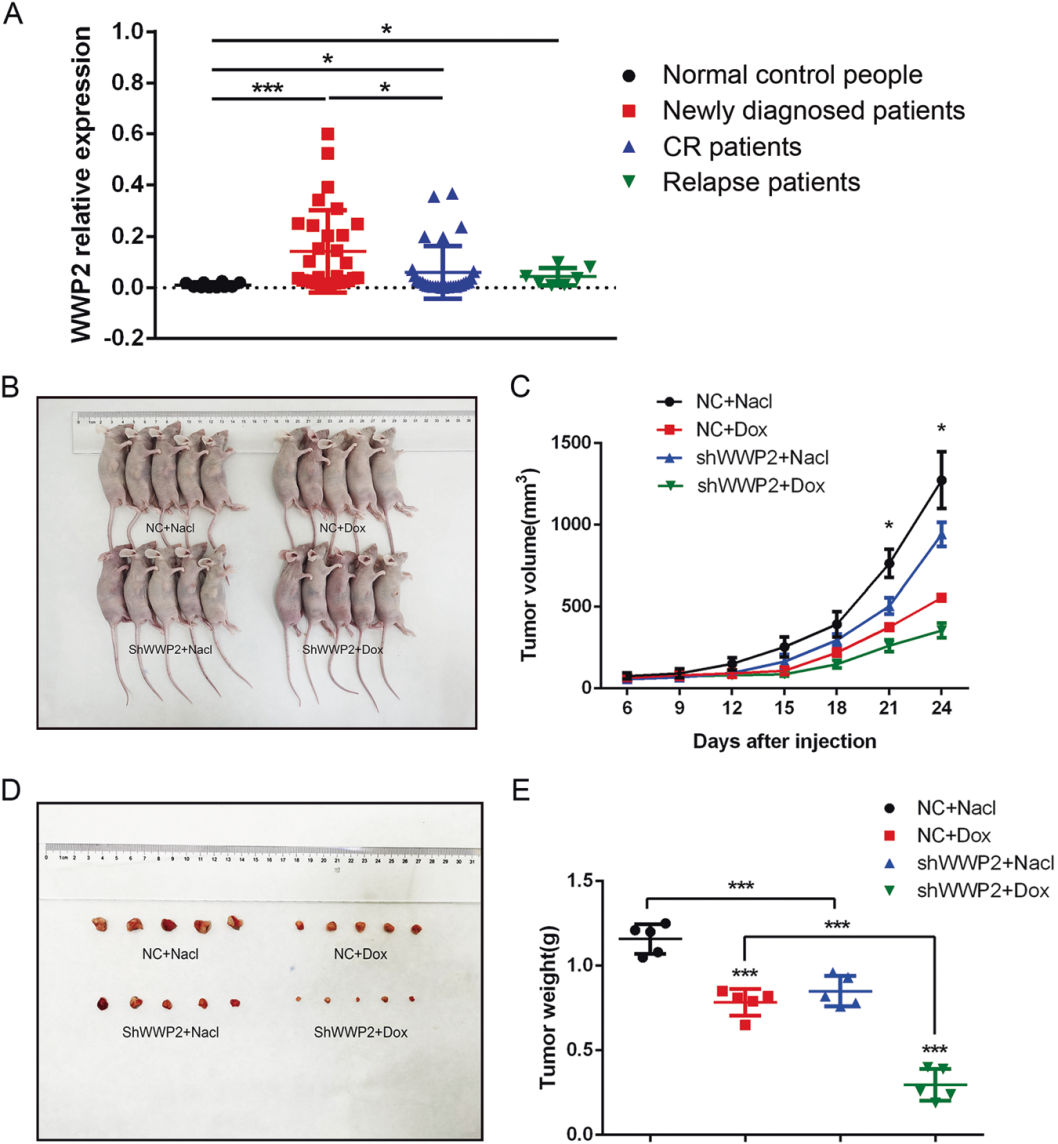
The WWP2 expression differences exist in different period ALL patients and normal control people, and knockout WWP2 inhibits ALL xenograft tumor growth. **A** The WWP2 relative expression levels of bone marrow samples of newly diagnosed ALL patients (*n* = 30), CR ALL patients (*n* = 30), relapse ALL patients (*n* = 7) and normal control people (*n* = 10) were evaluated by normalized fold expression by RT-qPCR. Data were shown as mean ± standard deviation (SD) (*p < 0.05, **p < 0.01, ***p < 0.001; unpaired Student’s *t* test). **B**-**E** The photos of nude mice and xenograft tumor excised showed the growing differences of xenograft tumors volume and weight at the end of observation period in NC + Nacl group (black), NC + Dox group (red), shWWP2 + Nacl group (blue) and shWWP2 + Dox group (green). Data were shown as mean ± SD, the line chart illustrates the variation of xenograft tumor volume growing in different groups (*n* = 5; *p < 0.05; one-way ANOVA with Bonferroni’s post-hoc test) and the scatter chart illustrates the weight of xenograft tumors excised of different groups (*n* = 5; *p < 0.05, **p < 0.01, ***p < 0.001; unpaired Student’s *t* test, two-way ANOVA with Bonferroni’s post-hoc test).

WWP2 relative expression in patients and normal control samples were evaluated by relative quantification using real-time PCR. The results showed WWP2 expression in newly diagnosed ALL patients (0.1405 ± 0.1609) was higher than that in CR ALL patients (0.0588 ± 0.1029) and normal control people (0.0099 ± 0.0092). And the expression of WWP2 in relapse ALL patients (0.0424 ± 0.0346) and CR ALL patients (0.0588 ± 0.1029) was higher than that in normal control people (0.0099 ± 0.0092) (Fig. 1A). It indicated that WWP2 expression differences existed in different period of ALL patients and normal control people, and WWP2 was related with ALL development.

### WWP2 has a relationship with clinicopathological characteristics in ALL patients

Next, we analyzed the clinicopathologic factors of ALL patients with WWP2 expression. Patients were divided into groups according to categorical variables (gender, FAB subtype, T/B subtype, BCR/ABL appearance, karyotype) and the median of continuous variables (age, blast cell proportion, WBC level, Hb level, PLT level). It was found that WWP2 expression was related with FAB subtype and WWP2 expressed higher in a larger proportion of blast cells in bone marrow blood tested by flow cytometry. While, there was no relationship with WWP2 expression and other clinicopathologic factors such as gender, age, T/B subtype, the proportion of blast cells (bone marrow smear), WBC level, Hb level, PLT level, BCR/ABL appearance and karyotype (Table 1).

**Table 1:** Correlation between WWP2 expression and clinicopathologic factors in ALL patients.

Clinicopathologic Factors	Cases	WWP2 expression	*P* value
Gender			0.162
Male	40	0.0731 ± 0.1059	
Female	27	0.1242 ± 0.1648	
Age			0.154
<42	33	0.0700 ± 0.1029	
≥42	44	0.1166 ± 0.1567	
FAB subtype			0.016*
L1	5	0.0265 ± 0.0103	
L2	54	0.0767 ± 0.1076	
L3	8	0.2500 ± 0.2195	
T/B subtype			0.458
T cell	8	0.1491 ± 0.2235	
B cell	59	0.0861 ± 0.1180	
Blast cell (Bone marrow smear)			0.493
<32%	33	0.0822 ± 0.1392	
≥32%	34	0.1048 ± 0.1299	
Blast cell (Flow cytometry)			0.032*
<32.8%	33	0.0583 ± 0.0984	
≥32.8%	34	0.1279 ± 0.1551	
WBC (*10^9^/L)			0.980
<5.03	34	0.0933 ± 0.1272	
≥5.03	33	0.0941 ± 0.1427	
Hb (g/L)			0.470
<100	33	0.1058 ± 0.1236	
≥100	34	0.0819 ± 0.1442	
PLT (*10^9^/L)			0.370
<131	32	0.1091 ± 0.1349	
≥131	35	0.0795 ± 0.1335	
BCR/ABL appearance			0.586
Positive	16	0.0780 ± 0.0940	
Negative	8	0.0531 ± 0.1273	
Karyotype			0.645
Normal	12	0.1827 ± 0.2074	
t (9;22)	4	0.1546 ± 0.1324	
Complex	4	0.1683 ± 0.1825	
Others	7	0.0828 ± 0.1111	

The significance of bold values is shown as follows: *p < 0.05; **p < 0.01.

Data loss exists in BCR/ABL appearance and Karyotype analysis.

### Knockout WWP2 inhibits the growth of ALL xenograft tumor

We established xenograft tumor model in order to investigate the WWP2 function in tumor growth. Nude mice were divided into two groups randomly and given subcutaneous injection of NC or shWWP2 Jurkat cells. Then we gave intraperitoneal injection of normal saline (NS) or Dox randomly in each group, aiming to evaluate WWP2 function to tumor growth under the stimulation of Dox. The results showed under the subcutaneous injection of the same Jurkat cells, tumors with Dox intraperitoneal injection had less volume and weight than tumors with NS intraperitoneal injection. And the volume and weight of tumor of NC + NS group is greater than that of shWWP2 + NS group. The volume and weight of tumor of NC + Dox group is greater than that of shWWP2 + Dox group (Fig. 1B–E). It indicated that Dox intraperitoneal injection inhibited tumor growing obviously and knockout WWP2 inhibited the growth of ALL xenograft tumor under both normal conditions and Dox stimulation.

### Knockout WWP2 enhances apoptosis of ALL xenograft tumor under the inducement of Dox

**Fig. 2:**
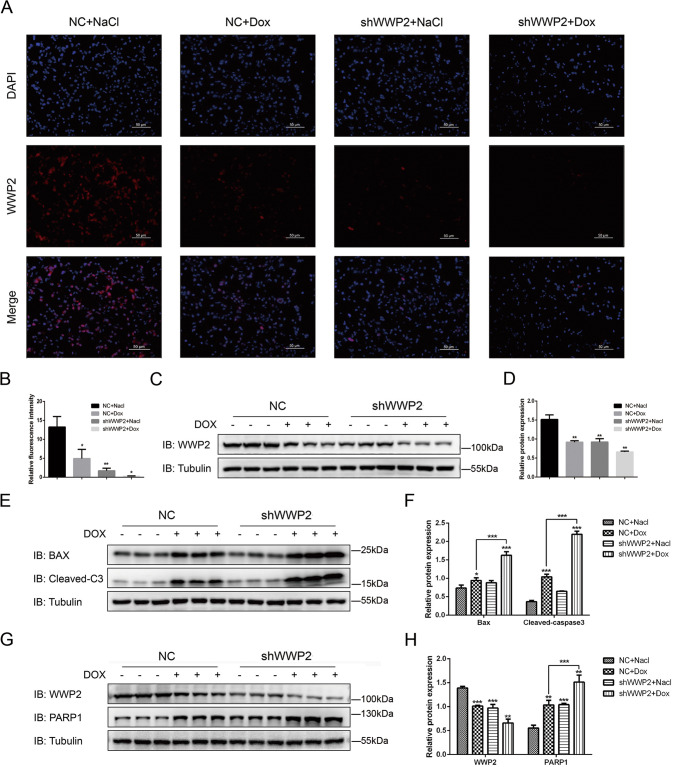
Knockout WWP2 enhances apoptosis level under inducement of Dox and WWP2 negatively regulates PARP1 stability in ALL xenograft tumor. **A**, **B** Immunofluorescence assays (WWP2 red; DAPI blue) was used to assess WWP2 expression level in different groups of xenograft tumors (scale bar 50 μm). Data were shown as mean ± SD (*p < 0.05, **p < 0.01; unpaired Student’s *t* test). **C**, **D** Western blot was used to assess WWP2 expression level in different groups of xenograft tumors. Data were shown as mean ± SD (*p < 0.05, **p < 0.01; unpaired Student’s *t* test). **E**, **F** Western blot was used to evaluate apoptosis protein (Bax and Cleaved-Caspase3 (Cleaved-C3)) expression in different groups of xenograft tumors. Data were shown as mean ± SD (*p < 0.05, **p < 0.01, ***p < 0.001; unpaired Student’s *t* test, two-way ANOVA with Bonferroni’s post-hoc test). **G**, **H** Western blot was used to evaluate PARP1 protein expression. Data were shown as mean ± SD (*p < 0.05, **p < 0.01, ***p < 0.001; unpaired Student’s *t* test, two-way ANOVA with Bonferroni’s post-hoc test).

We tested the expression of WWP2 through immunofluorescence assays and western blot. The results showed WWP2 expression in shWWP2+NS group is much lower than that in NC + NS group, which confirmed the WWP2 knockout efficiency in vivo. And the intraperitoneal injection of Dox reduced the expression of WWP2 (Fig. 2A–D). The apoptosis level in xenograft tumors was assessed by two apoptosis proteins (Bax and Cleaved-Caspase3(Cleaved-C3)) through western blot. The expression of Bax and Cleaved-C3 in both Dox groups is much higher than that in both NS groups, which proved the apoptosis-inducing efficiency of Dox in vivo. And the expression of Bax and Cleaved-C3 in shWWP2+Dox group is significantly higher than that in NC + Dox group (Fig. 2E, F). It indicated that knockout WWP2 enhanced apoptosis of ALL tumor cells induced by Dox in vivo.

### WWP2 negatively regulates PARP1 stability in ALL xenograft tumors

PARP1 was reported to be involved in apoptosis induced by oxidative stress and considered as a therapeutic target for cancers. In order to investigate the potential relationship between WWP2 and PARP1, we tested the expression of PARP1 by western blot meanwhile. It showed WWP2 influenced PARP1 stability and negatively regulate PARP1 expression in ALL xenograft tumors (Fig. 2G, H).

### WWP2 is related to apoptosis of Jurkat cells induced by Dox

As verified above, WWP2 was involved in apoptosis of ALL xenograft tumors and the downregulation of PARP1 may play a role in this process. Therefore, we further explored it in cellular level in order to confirm the results and elucidate this mechanism clearly.

**Fig. 3:**
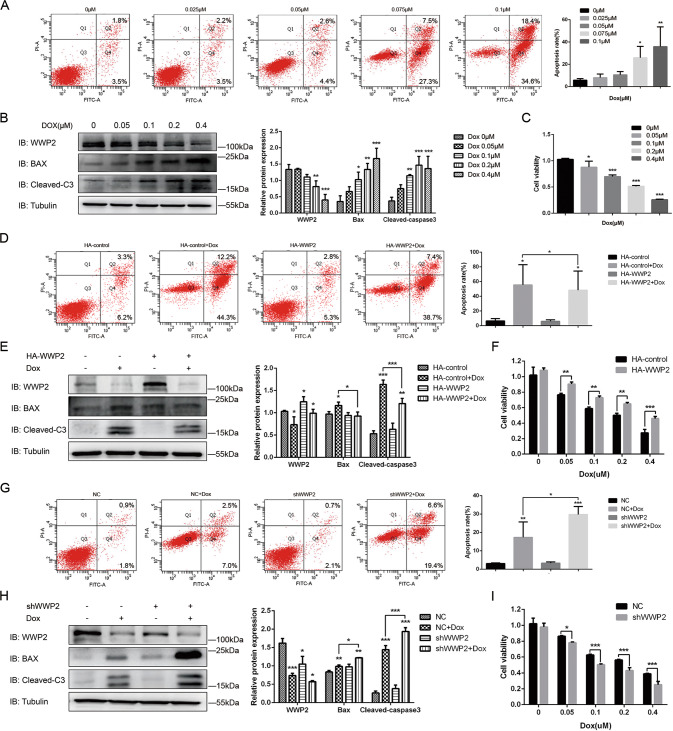
WWP2 overexpression alleviates apoptosis under the inducement of Dox in Jurkat cells while knockout WWP2 enhances this effect. **A**–**C** Concentration gradient of Dox was set for western blot and cell viability assay (0, 0.05, 0.1,0.2, 0.4 μM) and for flow cytometry analysis (0, 0.025, 0.05, 0.075, 0.1 μM). **A** Flow cytometry annexin-FITC/PI was used to assess apoptosis rate of Jurkat cells in different concentration of Dox. Data were shown as mean ± SD (*p < 0.05, **p < 0.01; one-way ANOVA with Bonferroni’s post-hoc test). **B** Western blot was used to evaluate apoptosis protein (Bax and cleaved-C3) expression of Jurkat cells in different concentration of Dox. Data were shown as mean ± SD (*p < 0.05, **p < 0.01, ***p < 0.001; one-way ANOVA with Bonferroni’s post-hoc test). **C** CCK-8 assay was used to assess the viability of Jurkat cells in different concentration of Dox. Data were shown as mean ± SD (*p < 0.05, **p < 0.01, ***p < 0.001; one-way ANOVA with Bonferroni’s post-hoc test). **D**–**F** Jurkat cells were transfected with HA-WWP2 or HA-control plasmids under the inducement of Dox for western blot and cell viability assay (0.2 μM) and for flow cytometry (0.075 μM). **D** Flow cytometry annexin-FITC/PI was used to assess apoptosis rate of Jurkat cells. Data were shown as mean ± SD (*p < 0.05, **p < 0.01, ***p < 0.001; unpaired Student’s *t* test, two-way ANOVA with Bonferroni’s post-hoc test). **E** Western blot was used to evaluate apoptosis protein (Bax and cleaved-C3) expression of Jurkat cells. Data were shown as mean ± SD (*p < 0.05, **p < 0.01, ***p < 0.001; unpaired Student’s *t* test, two-way ANOVA with Bonferroni’s post-hoc test). **F** CCK-8 assay was used to assess the viability of Jurkat cells. Data were shown as mean ± SD (*p < 0.05, **p < 0.01, ***p < 0.001; two-way ANOVA with Bonferroni’s post-hoc test). **G**–**I** Jurkat cells were infected with WWP2 knockout or control lentivirus under the inducement of Dox for western blot and cell viability assay (0.2 μM) and for flow cytometry (0.075 μM). **G** Flow cytometry, **H** western blot and **I** CCK-8 assays were performed as described above.

Given that no study has evaluate the effect of Dox-induced Jurkat cells apoptosis on WWP2 expression, we set a concentration gradient of Dox (0, 0.05, 0.1,0.2, 0.4 μM) for western blot and cell viability assay and concentration gradient of Dox (0, 0.025, 0.05, 0.075, 0.1 μM) for flow cytometry analysis. Results showed that the cell viability was decreasing while the apoptosis rate was increasing with growing Dox concentration (Fig. 3A, C). As Dox concentration increasing, the expression of WWP2 increased slightly at the beginning and following decreased gradually, while apoptosis proteins (Bax and Cleaved-C3) expressed higher and higher (Fig. 3B). The results above indicated that WWP2 was involved in apoptosis of ALL cells induced by dox.

### WWP2 overexpression alleviates apoptosis of Jurkat cells induced by dox while knockout WWP2 enhances this effect

In order to explore the role of WWP2 in ALL cell apoptosis, we made WWP2 overexpression and WWP2 knockout in Jurkat cells under stimulation of Dox (0.075 μM for flow cytometry; 0.02 μM for western blot and cell viability assay), respectively.

It was found that the apoptosis rate and apoptosis protein expression were at a lower level both in NC and WWP2 overexpression Jurkat cells, and Dox stimulation increased apoptosis level significantly. As a result, WWP2 overexpression alleviated the apoptosis rate and decreased apoptosis protein (Bax and Cleaved-C3) expression under Dox stimulation compared with NC + Dox group (Fig. 3D, E). And WWP2 overexpression increased cell viability in different concentration of Dox (Fig. 3F). Similarly, Apoptosis level of both NC and WWP2 knockout Jurkat cells increased under Dox stimulation. However, WWP2 knockout increased the apoptosis rate and the expression of Bax and Cleaved-C3 more under Dox stimulation compared with NC + Dox group (Fig. 3G, H). And WWP2 knockout decreased cell viability under Dox stimulation in different concentration (Fig. 3I). The series of tests suggested WWP2 overexpression alleviated apoptosis of Jurkat cells, but knockout WWP2 enhanced this effect contrarily.

### WWP2 interacts with PARP1 and the interaction is weakened under the stimulation of Dox in Jurkat cells

**Fig. 4:**
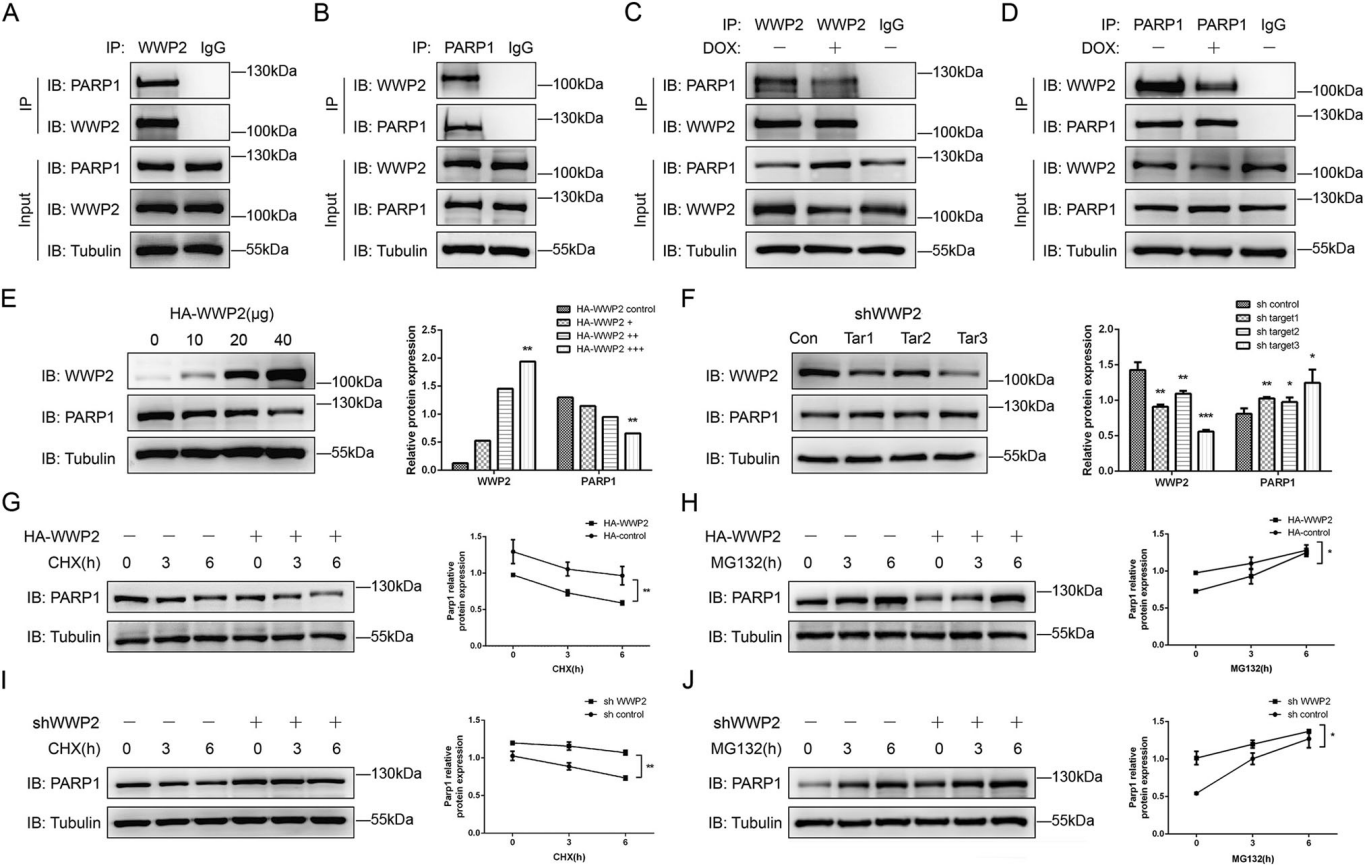
WWP2 interacts with PARP1 and negatively regulates PARP1 through proteosome degradation. **A**, **B** Coimmunoprecipitation assays were performed to assess bidirectional interaction between WWP2 and PARP1. **C**, **D** Coimmunoprecipitation assays were performed to assess bidirectional interaction between WWP2 and PARP1 under normal condition and stimulation of Dox (0.2 μM). **E** Western blot was carried out to assess PARP1 expression level with overexpression of WWP2 gradually. Data were shown as mean ± SD (*p < 0.05, **p < 0.01; one-way ANOVA with Bonferroni’s post-hoc test). **F** Western blot was carried out to assess the efficiency of WWP2 knockdown by three target shRNA-WWP2 and the expression of PARP1 in Jurkat cells. Data were shown as mean ± SD (*p < 0.05, **p < 0.01, ***p < 0.001; unpaired Student’s *t* test). **G**–**J** Western blot was performed to assess PARP1 expression in Jurkat cells with WWP2 overexpression under different durations of CHX (**G**) or MG132 (**H**), and PARP1 expression in Jurkat cells with WWP2 knockout under different durations of CHX (I) or MG132 (**J**). Data were shown as mean ± SD (*p < 0.05, **p < 0.01; two-way ANOVA with Bonferroni’s post-hoc test).

In previous tests, we have proved WWP2 negatively regulate PARP1 expression in vivo. Subsequently, coimmunoprecipitation tests indicated WWP2 interacted with PARP1 in ALL cells. This interaction was bidirectional in Jurkat cells and this interaction was bidirectionally weakened under Dox stimulation (Fig. 4A–D). It indicated that WWP2 and PARP1 interacted with each other by some mechanism in ALL and the interaction was affected by Dox stimulation. The results above also suggested that the interaction between WWP2 and PARP1 was the basis of WWP2 regulating PARP1.

### WWP2 negatively regulates PARP1 and mediates proteasome-degradation of PARP1

We successively transfected 0, 10, 20, 40 μg WWP2 plasmid with HA tag into Jurkat cells. It was found PARP1 expression decreased gradually as increasing expression of WWP2 (Fig. 4E). We tested PARP1 expression in three targeted shWWP2 Jurkat cells and higher expressions of PARP1 were observed in all of shWWP2 Jurkat cell lines (Fig. 4F). It suggested that WWP2 negatively regulated PARP1 in Jurkat cells.

It was verified that WWP2 had interaction with PARP1 and the negative regulation on PARP1 so far. Therefore, we further investigated WWP2 downregulated PARP1 whether by inhibiting PARP1 transcription or promoting proteasome degradation of PARP1. The protein synthesis inhibitor CHX and proteasome inhibitor MG132 were used to determine the effect of WWP2 on PARP1.

In Jurkat cells with transfection of HA-WWP2 plasmid, PARP1 expression was lower and attenuated faster than that in cells with transfection of empty vector under the treatment of CHX (Fig. 4G). And PARP1 expressed lower but accumulated more obviously than that in controls under the treatment of MG132 (Fig. 4H). In Jurkat cells with WWP2 knockout, PARP1 expression was higher and attenuated more slowly than that in normal controls under the treatment of CHX (Fig. 4I), while, PARP1 expressed higher and accumulated more slowly compared with that in normal Jurkat cells under the treatment of MG132 (Fig. 4J). The results above showed WWP2 overexpression contributed to a reduction of PARP1 half-life while WWP2 knockout could prolong it. A decreasing trend of PARP1 was tested in CHX assays, but accumulation of PARP1 existed in both MG132 assays whether WWP2 was overexpressed or knocked out. It indicated that MG132 did affect the degradation of PARP1 and WWP2 regulated PARP1 through proteasome degradation.

### WWP2 mediates polyubiquitination of PARP1

**Fig. 5:**
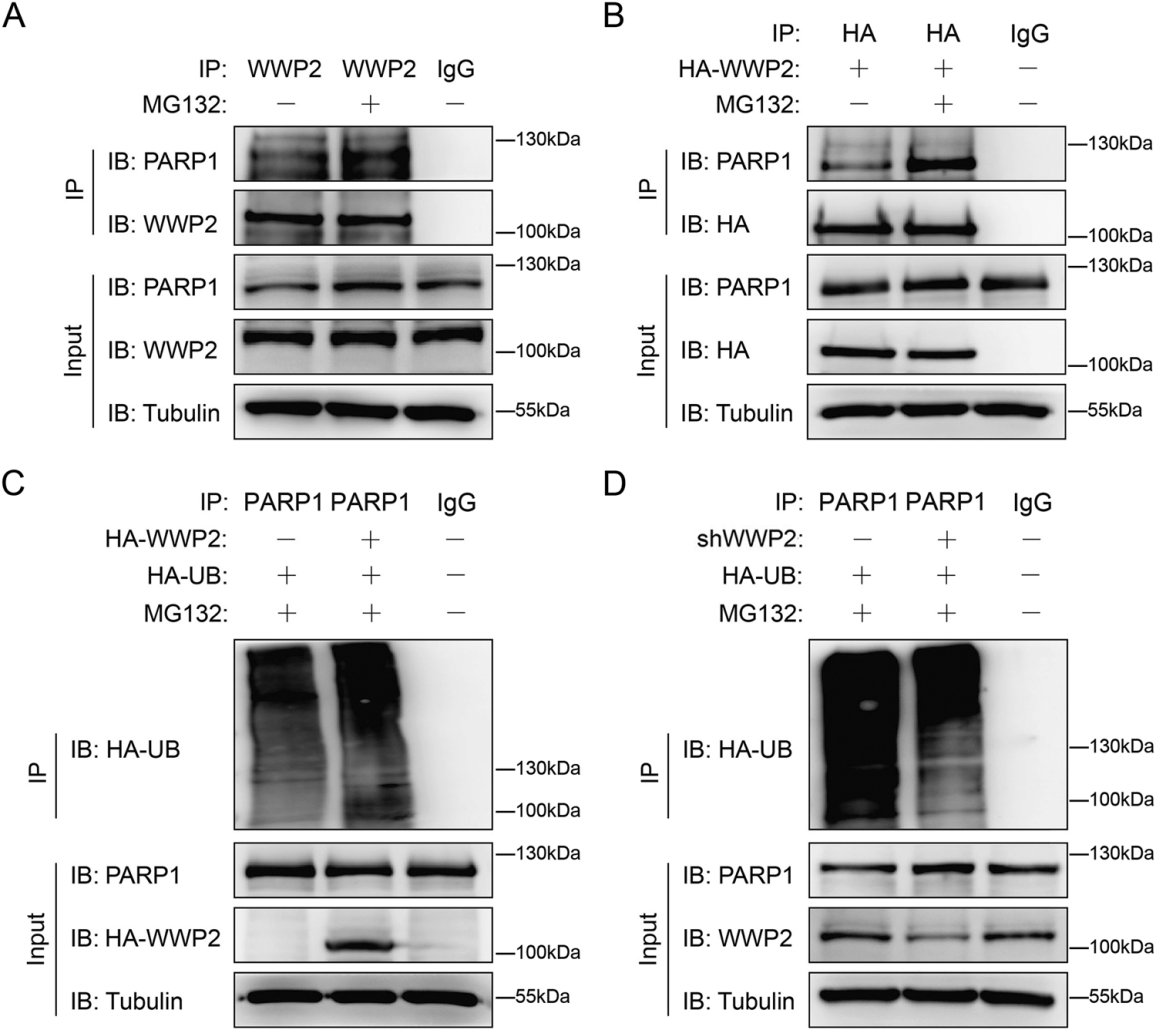
WWP2 degrades PARP1 through polyubiquitination-proteasome way. **A** Coimmunoprecipitation assays were performed to assess endogenous interaction between WWP2 and PARP1 with or without MG132 treatment in Jurkat cells. **B** Coimmunoprecipitation assays were performed to assess half-exogenous interaction between WWP2 and PARP1 with or without MG132 treatment in Jurkat cells. **C** HA-WWP2 and HA-ubiquitin (UB) were co-expressed with or without MG132 treatment in Jurkat cells, PARP1 was purified by IP and PARP1 ubiquitination levels were assessed by anti-HA antibodies. **D** ShWWP2 and HA-UB were co-expressed with or without MG132 treatment in Jurkat cells, PARP1 was purified by IP and PARP1 ubiquitination levels were assessed by anti-HA antibodies.

As mentioned above, WWP2 regulated PARP1 through proteasome degradation. Considering WWP2 was functioned as E3 ubiquitin ligase, it strongly suggested that PARP1 was one of the substrates of WWP2. We further studied whether WWP2 mediated polyubiquitination of PARP1. Firstly, we confirmed a stronger interaction in Jurkat cells between WWP2 and PARP1 when proteosome degradation activity was blocked by MG132 endogenously and half-exogenously (Fig. 5A, B). It proved inhibiting proteosome degradation activity enhanced the interaction between WWP2 and PARP1. Next, we tested polyubiquitination extent of PARP1 by transfection of HA-ubiquitin and using MG132 in WWP2-overexpression assay and WWP2 knockout assay respectively. The polyubiquitination level of PARP1 was increased after WWP2 overexpressed, while decreased after WWP2 knocked out (Fig. 5C, D). These results suggested WWP2 mediate PARP1 proteosome degradation by polyubiquitination of PARP1 in Jurkat cells.

## Discussion

ALL has always been huge threat to human health and finding effective therapeutic target for ALL make great significance. In this study, we firstly analyzed WWP2 expression in different period ALL patients and the relationship with clinicopathological factors. We explored the role of WWP2 in ALL growth and apoptosis in vitro and vivo, and elaborated that WWP2 downregulated PARP1 through ubiquitin-proteasome degradation in ALL cells for the first time in order to provide a new idea for target treatment for ALL.

WWP2, as a member of NEDD4 family ubiquitin ligases, was discovered for binding to atrophin-1 by yeast two-hybrid and vitro binding analysis in 1997 (ref. [9]). WWP2 widely expressed [25] and played a crucial part in pathogenesis in different types of tumors such as lung cancer, gastric cancer and liver cancer by regulating ubiquitin-dependent degradation of substrate proteins [14, 15, 26] In our study, we firstly analyzed ubiquitin ligase WWP2 expression level in ALL patients and the relationship with clinicopathological factors. The WWP2 expression difference in different period of ALL patients and normal control people and the relationship with FAB subtype and proportion of blast cells in bone marrow blood tested by flow cytometry indicated that WWP2 played a role in ALL development. And subsequent experiment proved that knockout WWP2 inhibit growth and aggravated apoptosis in ALL xenograft tumor. The experiment in cellular level also confirmed overexpression of WWP2 improved cells viability and alleviated apoptosis in ALL, while, knockout WWP2 inhibited cells viability and enhanced apoptosis in ALL, which was consistent with our results in vivo. It was obviously that WWP2 played a role in ALL growth and apoptosis.

Protein ubiquitination is involved in a wide range of cellular biological process [27]. E3 ubiquitin ligase plays an important part in ubiquitin-protein system for binding substrate specifically. Proteins such as membrane proteins, cell cycle regulators, transcription factors, tumor suppressors and oncogenes, are ubiquitinated and play roles in different cellular activities [4, 5, 7]. Ubiquitin-proteasome pathway is reported to be essential in leukemia [28–30] and ubiquitin ligases participated in the development and resistance of different leukemia [31–34]. And it was reported that E3 ubiquitin ligases also play other roles in leukemia. HERC1, as one member of HECT-type ubiquitin ligases, was recently discovered to expressed aberrantly in myeloid-related disorders and act as potential player in leukemic cell differentiation [35, 36]. In our study, we have proved that knockout WWP2 enhanced ALL apoptosis induced by dox in vitro and vivo. Therefore, we had a thought that WWP2 play roles in ALL by regulating substrate protein as ubiquitin ligase through ubiquitin-proteasome pathway.

PARP1 is known to play crucial roles in DNA damage response and promotes DNA repair serving as early sensor of DNA damage [37]. But PARP1 induces cell death for genome integrity in the case of extensive damage [17]. With the research goes further, PARP1 is reported be involved in many cellular processes such as cell apoptosis [18, 23], chromatin remodeling [38] and gene expression [20, 39]. PARP1 is also involved in malignant tumors development and resistance of cancers [40–44], and it is regarded as a potential therapeutic target in certain leukemia [24, 45]. Particularly, previous studies showed PARP1 regulated in cancers by inducing cell apoptosis [23, 46]. These years, PARP1 is reported to regulate in cardiac disease serving as substrate protein of many ubiquitin ligases and participating in ubiquitin-proteasome pathway [7, 47], which broaden the molecular mechanism study of PARP1. In this study, we firstly discovered WWP2 negatively regulated and interacted with PARP1 in ALL, and this interaction reduced under the apoptosis induced by Dox. Considering ubiquitin ligase function of WWP2 and the role of PARP1 in ubiquitin-proteasome as reported, it is suggested PARP1 may be a substrate of WWP2 in ALL and contribute to the regulation of apoptosis of ALL. CHX and MG132 assays further proved that WWP2 down-regulated PARP1 by proteasome degradation. And the polyubiquitination level of PARP1 in overexpression WWP2 and knockdown WWP2 assays showed WWP2 mediate PARP1 expression by polyubiquitination proteasome pathway.

Doxorubicin is widely applied in clinical therapy of many malignant tumors such as leukemia, lymphomas and several solid tumors [48]. It works through intercalation into double-strand DNA, inhibition of topoisomerase II and formation of ROS leading to cell apoptosis [49]. The apoptosis models in vitro and vivo in this study were induced by Dox, which could be regarded as a microcosm of ALL in treatment of Dox. The findings in this study suggested WWP2 was involved in ALL treatment process. The reduced interaction between WWP2 and PARP1, the decreased expression of WWP2 and increased expression of PARP1 participated in ALL apoptosis, which was consistent with PARP1 function mentioned above. Therefore, the regulation of PARP1 by WWP2 was probably potential target for ALL therapy.

According to results above, we firstly reported a new function of WWP2 and provide insight into a related mechanism in ALL. We determined the different expression of WWP2 in different period ALL patients and normal control people and analyzed relationship with clinicopathological factors for the first time. We proved knockout WWP2 inhibited ALL growth and enhanced ALL apoptosis in vitro and vivo, while overexpressed WWP2 showed opposite effect. We also illustrated WWP2 mechanically down-regulated PARP1 by polyubiquitinated-proteosome degradation in ALL. These findings suggested WWP2 played a role in ALL development as well as growth and apoptosis of ALL, and displayed a regulatory pathway of PARP1, which provide a new potential therapeutic target for the treatment of ALL.

## Materials and methods

### Patients and samples

Bone marrow blood samples were collected from 30 newly diagnosed ALL patients, 30 CR ALL patients, 7 relapse ALL patients and 10 normal control people from December 2018 to October 2020 in the Department of Hematology of the First Hospital of China Medical University. Diagnosis of patients were based on morphology, immunology, cytogenetics and molecular biology (MICM) according to WHO classification criteria [50] and normal control samples were selected with no malignancy and infectious disease. The mononuclear cells of bone marrow blood were collected after the centrifugation of samples at 800 × *g* for 20 min. All research was approved by the Ethics Committee of the First Hospital, China Medical University (No. [2021]110).

### RNA extraction and RT‑qPCR assay

RNA from bone marrow blood samples was extracted by TRIzol (Takara Bio, Japan), and reverse transcription was performed according to the manufacturer’s protocol of PrimeScript™ RT reagent Kit (Takara Bio, Japan). The PCR amplification of cDNA fractions was conducted by TB Green® Premix Ex Taq™ II (Takara Bio, Japan). The primer sequences were as follows: WWP2: forward primer: 5′-GGTGCGATACTTTGTGGACCAC-3′, reverse primer: 5′-GATACTTCCACCGAAAACTGCGG-3′, GAPDH: forward primer: 5′-CACCCACTCCTCCACCTTTG-3′, reverse primer: 5′-CCACCACCCTGTTGCTGTAG-3′. Relative expression levels were calculated using 2^−ΔCt^ method.

### Xenograft model

The study was approved by the Animal Subjects Committee of China Medical University (No. CMU2021473) and all animal experiment follow the NIH Guide for the Care and Use of Laboratory Animals. Four-week-old BALB/c nude mice were purchased from Vital River Laboratories (Beijing, China). Mice were randomly divided into two groups for subcutaneous injection of normal control (NC) or shWWP2 Jurkat cells (1 × 10^7^/200 μl) with cell organoid culture hydrogel (Biozellen, USA). When the tumor size reached 80 mm^3^, the tumor-bearing mice were randomly given normal saline (NS) or Dox (2 mg/kg/d) intraperitoneal injection for 7 days, which were divided into four groups (NC + Nacl group, NC + Dox group, shWWP2+Nacl group and shWWP2+Dox group) (*n* = 5/group). Tumors were observed and diameters (x, y) were measured every 3 days, and tumor volumes (*V*) were calculated as *V* = 1/2 xy^2^(mm^3^). On the 20th day, the mice were killed and photographed, and then the tumors were excised, weighed, and photographed. There was no blinding in this experiment.

### Antibodies and reagents

Polyclonal rabbit anti-WWP2 (ab103527, Abcam, USA, WB: 1:1000; 12197-1-AP, Proteintech, China, WB: 1:1000, IF: 1:200), monoclonal rabbit anti-PARP1 (9532S, Cell Signaling Technology, USA, WB: 1:1000), monoclonal rabbit anti-Cleaved-caspase3 (9664 S, Cell Signaling Technology, USA, WB: 1:1000), monoclonal mouse anti-Bax (60267-1-Ig, Proteintech, China, WB: 1:1000), monoclonal rabbit anti-HA (3724S, Cell Signaling, USA, WB: 1:1000), polyclonal rabbit anti-β-tubulin (10094-1-AP, Proteintech, China, WB: 1:1000), Goat Anti-Rabbit IgG (A21020, Abbkine, China, WB: 1:10,000), Goat Anti-Mouse IgG (A21010, Abbkine, China, WB: 1:10,000) and Donkey anti-Rabbit IgG Secondary Antibody and Alexa Fluor 594 (A21207, Invitrogen, USA, IF: 1:500)were obtained commercially. MG132 (A2585; 20 μM) and cycloheximide (CHX) (A8244; 100 μM) were obtained from ApexBio (USA) and reconstituted in DMSO. Doxorubicin (Dox) (D8740; 25 mg) was obtained from Solarbio (China) and reconstituted in DMSO.

### Cell culture

Human acute lymphoblastic leukemia Jurkat cell line was procured from iCell Bioscience Inc (China), and recently authenticated by STR profiling and tested for mycoplasma contamination. Cells were cultured in RPMI-1640 medium (HyClone, USA) with 10% fetal bovine serum (HyClone, USA) in a humidified atmosphere of 5% CO_2_ at 37 °C.

### Plasmids construction and transfection

Plasmids encoding full-length human WWP2 (Genechem, China) and ubiquitin (Genechem, China) were cloned into HA-tagged destination vectors for immunoprecipitation or immunoblotting. INVI DNA RNA Transfection Reagent (Invigentech, USA) was used in plasmid transfection following the manufacturer’s instructions.

### Knock out WWP2 in Jurkat cells

Control and WWP2 shRNAs were obtained from GeneChem (China). WWP2 silencing was performed with lentivirus and puromycin was used for selecting shWWP2 Jurkat cells. To prevent off-target effects, three sequences were employed:

- WWP2 shRNA-1: GGAGAACAAAGGCAGCGTTGT
- WWP2 shRNA-2: GCCAACTGTTGATCTGGGAAA
- WWP2 shRNA-3: GTCAAGAACTCAGGCCACAGT

The efficiency of WWP2 knockdown by shRNA was confirmed by Western blot analysis.

### Cell viability assay

Cell Counting Kit-8 assay (Bimake, USA) was used for evaluating Jurkat cell viability. WWP2 overexpressed, WWP2 knockdown and control Jurkat cells stimulated with Dox in different concentration or not were seeded into 96-well plates at 5 × 10^3^ cells/well in 100 μl RPMI-1640 complete medium. CCK-8 reagents were added into wells at 10 μl/well and cells were further incubated for 2 h. Cell viability (optical density) was measured at 450 nm by a Bio-Rad microplate reader (Model 680; Bio-Rad Laboratories, Inc., Hercules, CA, USA).

### Flow cytometry analysis and annexin-FITC/PI staining

Annexin V, FITC Apoptosis Detection Kit (Dojindo, Japan) was used for detecting apoptosis rate of Jurkat cells in different treatment through flow cytometry. Cells were incubated in 800 μl binding buffer with 5 μl annexin V for 30 min and 2 μl PI solution for 5 min at room temperature (RT) in the dark. Finally, apoptotic cells were identified and quantified with flow cytometer (BD LSRFortessa, USA).

### Co-immunoprecipitation and immunoblotting

Cells were washed with PBS three times and lysed with cell lysis buffer (50 mmol/L Tris, 137 mmol/L NaCl, 1 mmol/L EDTA, 10 mmol/L NaF, 0.1 mmol/L Na_3_VO_4_, 1% NP-40, 1 mmol/L DTT, 10% glycerol, pH 7.8 and 100× protease inhibitor (Roche, Switzerland). After centrifugation (4 °C, 13,300 rpm, 15 min), the cell lysates were incubated with specific antibodies and 30 μl of magnetic beads (Bimake, USA) at 4 °C for 12 h. Then, the bound complexes were washed with cell lysis buffer and subjected to SDS-PAGE. Protein samples were separated by 8% or 12% SDS-polyacrylamide gels and transferred to PVDF membranes (Millipore USA). After blocking with 5% bovine serum albumin (BSA) in Tris-buffered saline containing Tween (TBST) at RT for 1 h, the membranes were incubated with corresponding primary antibody diluted in 1% BSA at 4 °C overnight. Membranes were washed in TBST before and after incubation in secondary antibodies for 1 h at RT the next day. Image J 1.52v (National Institutes of Health, USA) was used to quantify the immunoreactive bands.

### Immunofluorescence staining

Paraffin‐sectioned slides from xenograft tumors excised from nude mice were deparaffinized and rehydrated by dimethylbenzene and ethanol. Antigen retrieval was performed by incubating slides in 0.01 mol/L citrate buffer (pH 6.0) at 95 °C for 20 min. The samples were then blocked with 1% BSA in PBS with 0.3% Triton X-100 for 1 h and incubated with primary antibody at 4 °C overnight. The next day, slides were rinsed with PBS before and after the incubation in fluorescent secondary antibody for 1 h at RT in the dark, which were used for fluorescently labeling in tumor tissues. Cell nuclei were counter‐stained with DAPI. Digital images were observed and captured with a fluorescence microscope (BX61, Olympus, Japan).

### Statistical analysis

Data are presented as the mean ± standard deviation (SD). *F*-test was performed to evaluate the homogeneity of variance and Shapiro-Wilk test was used for evaluating data normality. Unpaired Student’s *t* test, one-way ANOVA and two-way ANOVA followed by Bonferroni’s post-hoc test were performed to assess differences in multiple groups, which involved one and two factors, respectively. Experiments in this study were replicated three times for statistical analysis. *P*-values were adjusted for multiple comparisons when applicable. All data were analyzed by SPSS 21.0 software (IBM SPSS, USA), *P* < 0.05 was considered statistically significant.

## Supplementary information

Supplemental material-Original western blot

## Data Availability

All data generated or analyzed during this study are included in this published article and its supplementary information files.
