# Supplementary material for: The role of E3 ubiquitin ligase WWP2 and the regulation of PARP1 by ubiquitinated degradation in acute lymphoblastic leukemia
Source: Cell Death Discov. 2022 Oct 18;8:421. doi: 10.1038/s41420-022-01209-9 (PMC9579143; doi:10.1038/s41420-022-01209-9)
Supplement: Supplementary file 1 — Supplemental material-Original western blot [file 41420_2022_1209_MOESM1_ESM.pptx]

## Slide 1
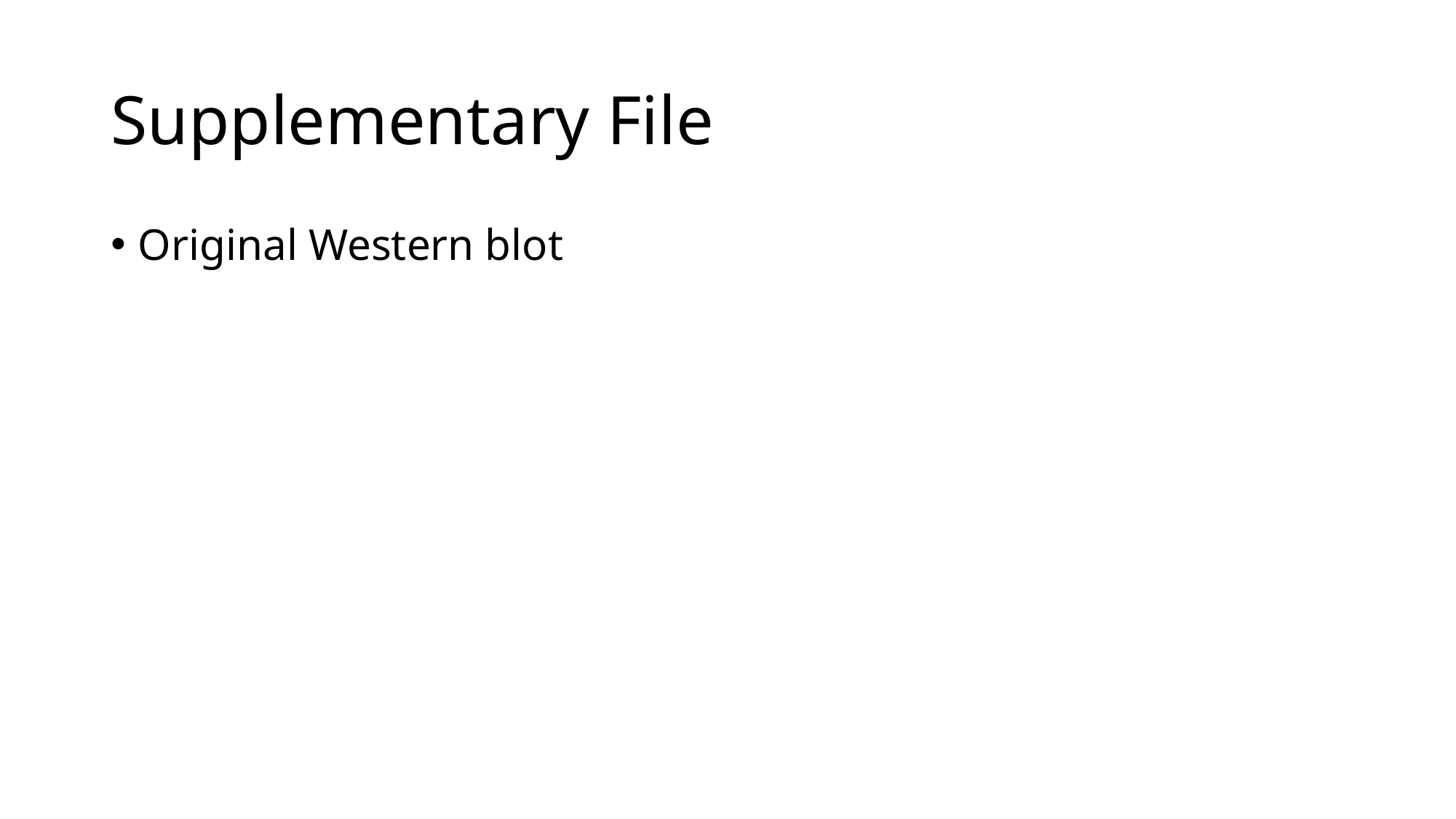

# Supplementary File
Original Western blot

## Slide 2
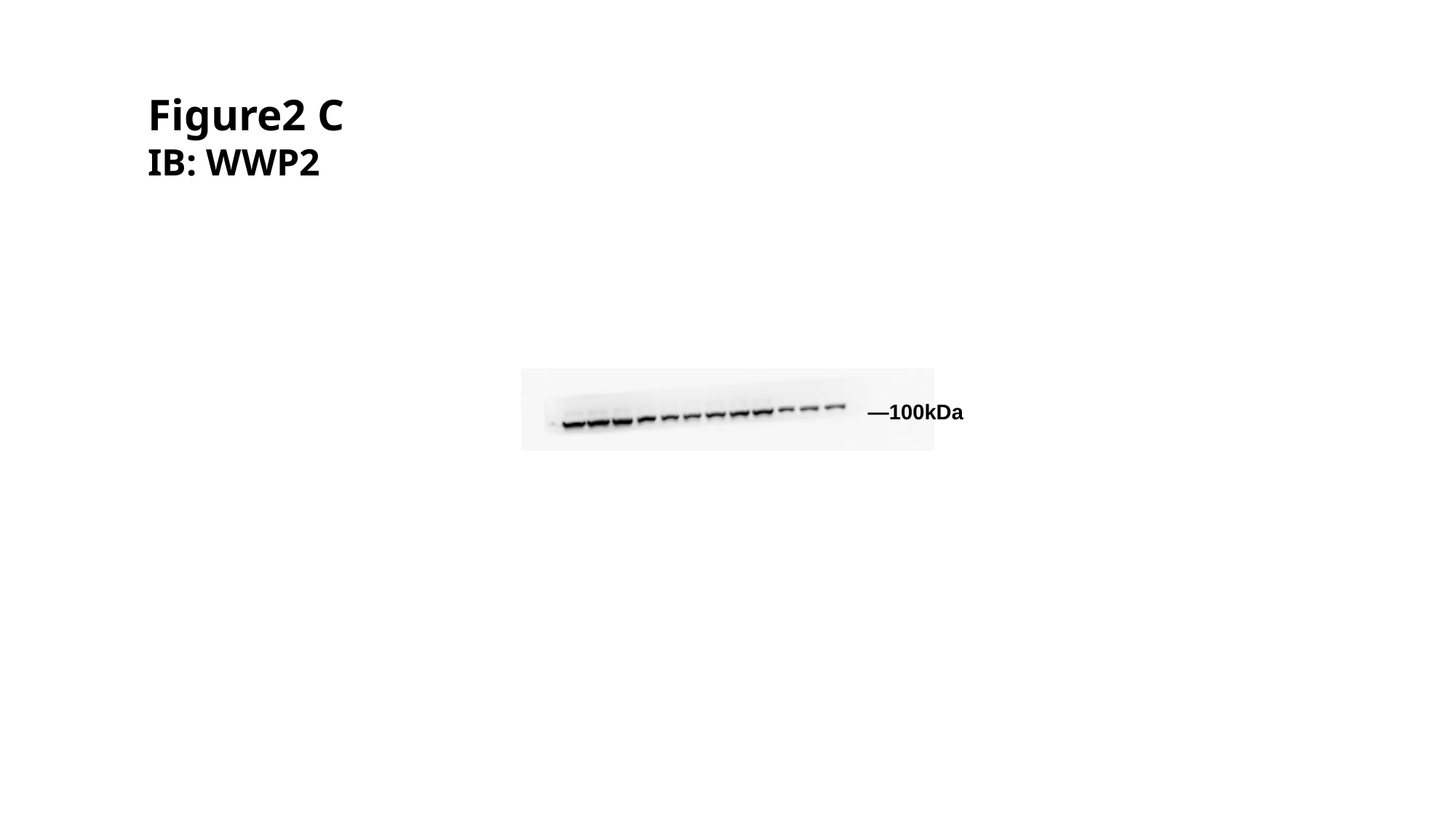

Figure2 C
IB: WWP2
—100kDa

## Slide 3
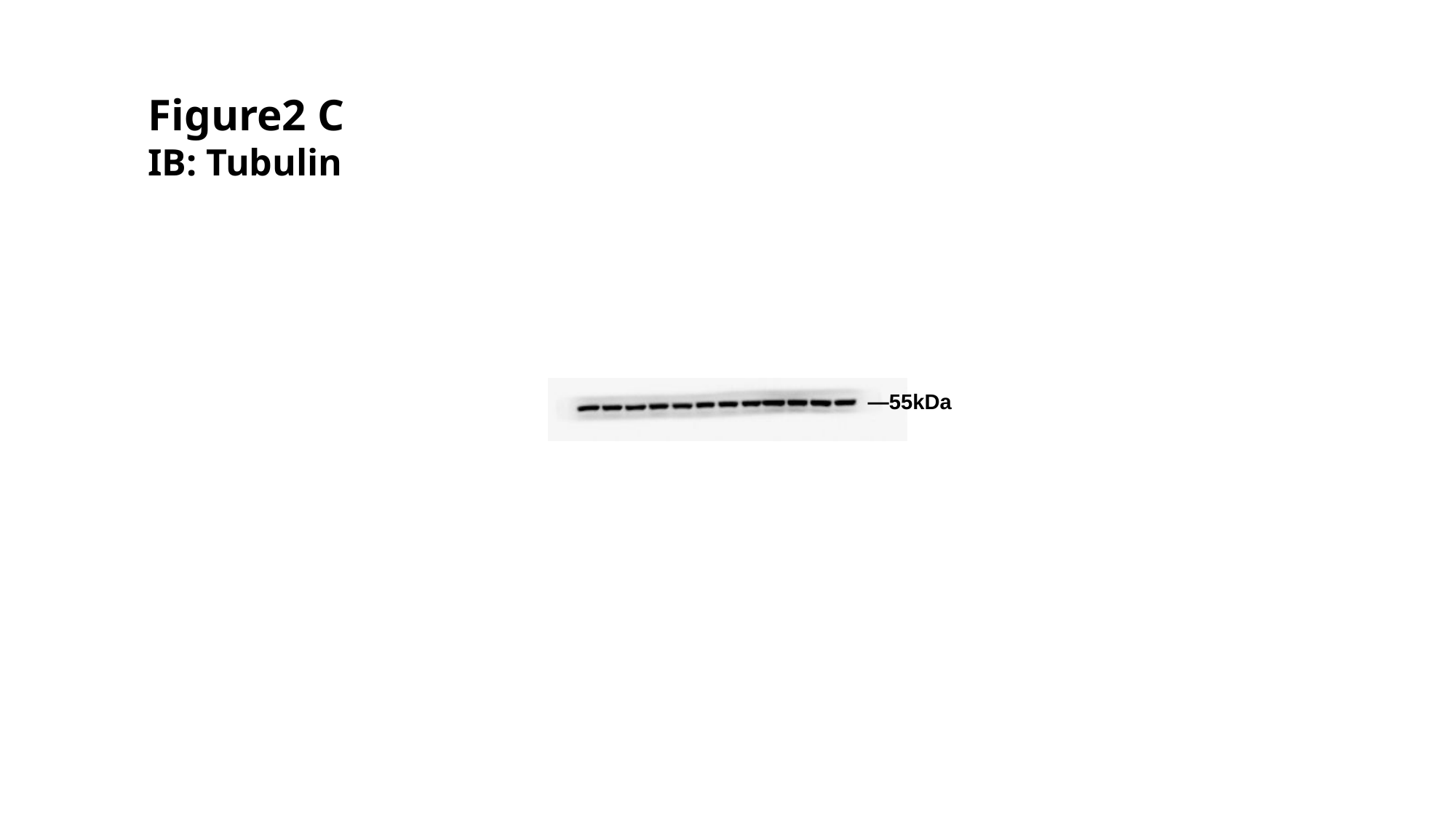

Figure2 C
IB: Tubulin
—55kDa

## Slide 4
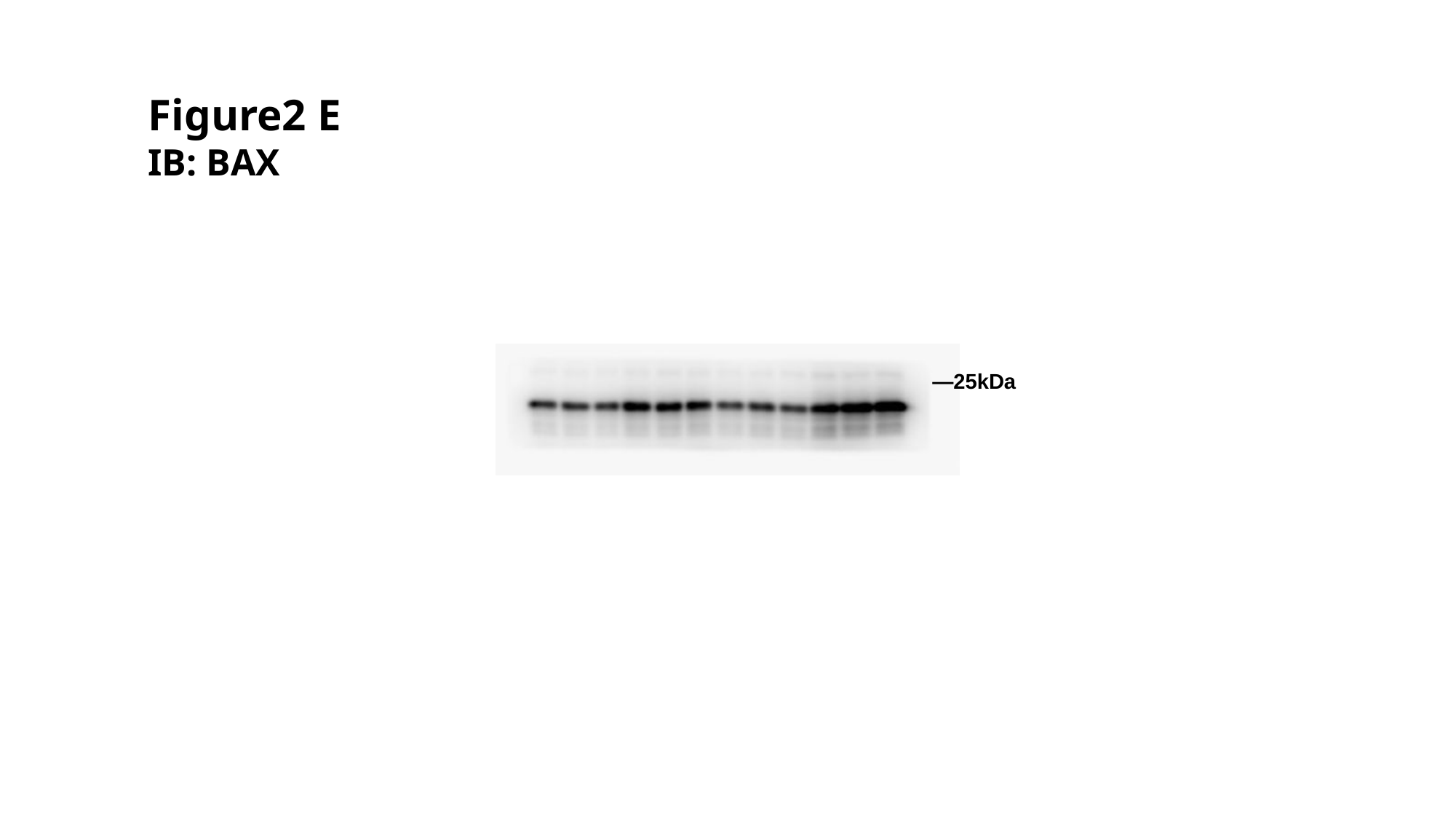

Figure2 E
IB: BAX
—25kDa

## Slide 5
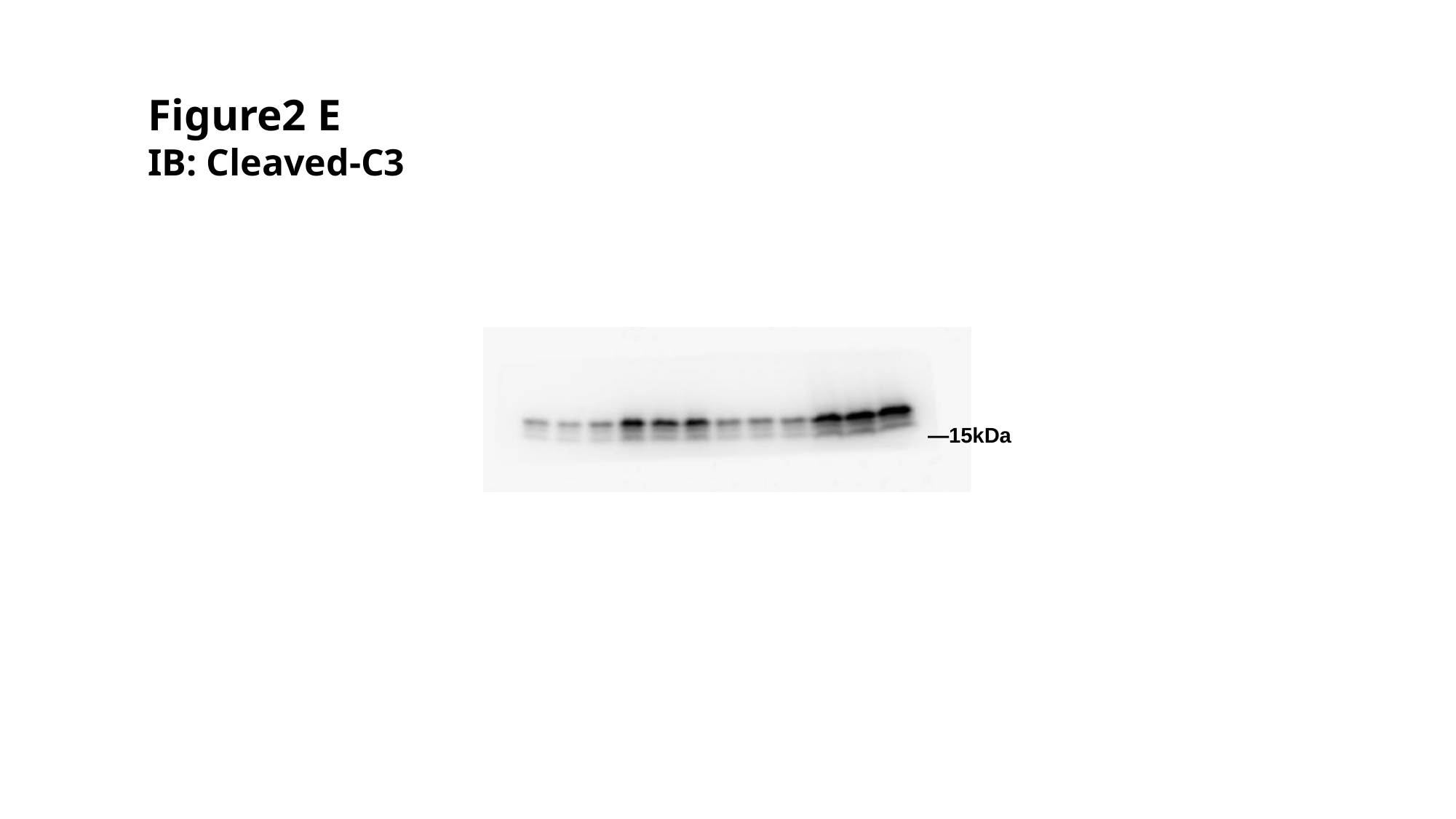

Figure2 E
IB: Cleaved-C3
—15kDa

## Slide 6
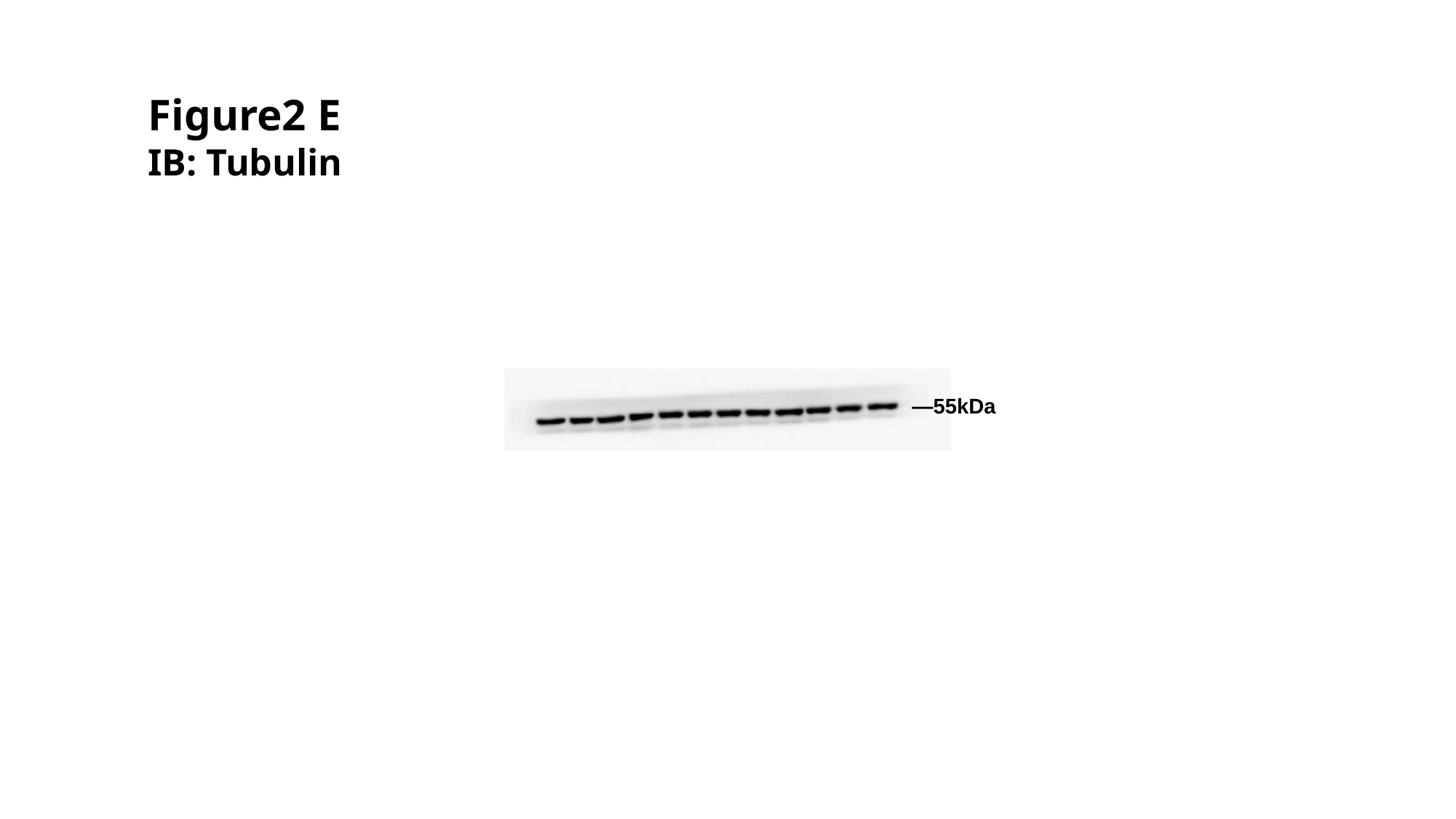

Figure2 E
IB: Tubulin
—55kDa

## Slide 7
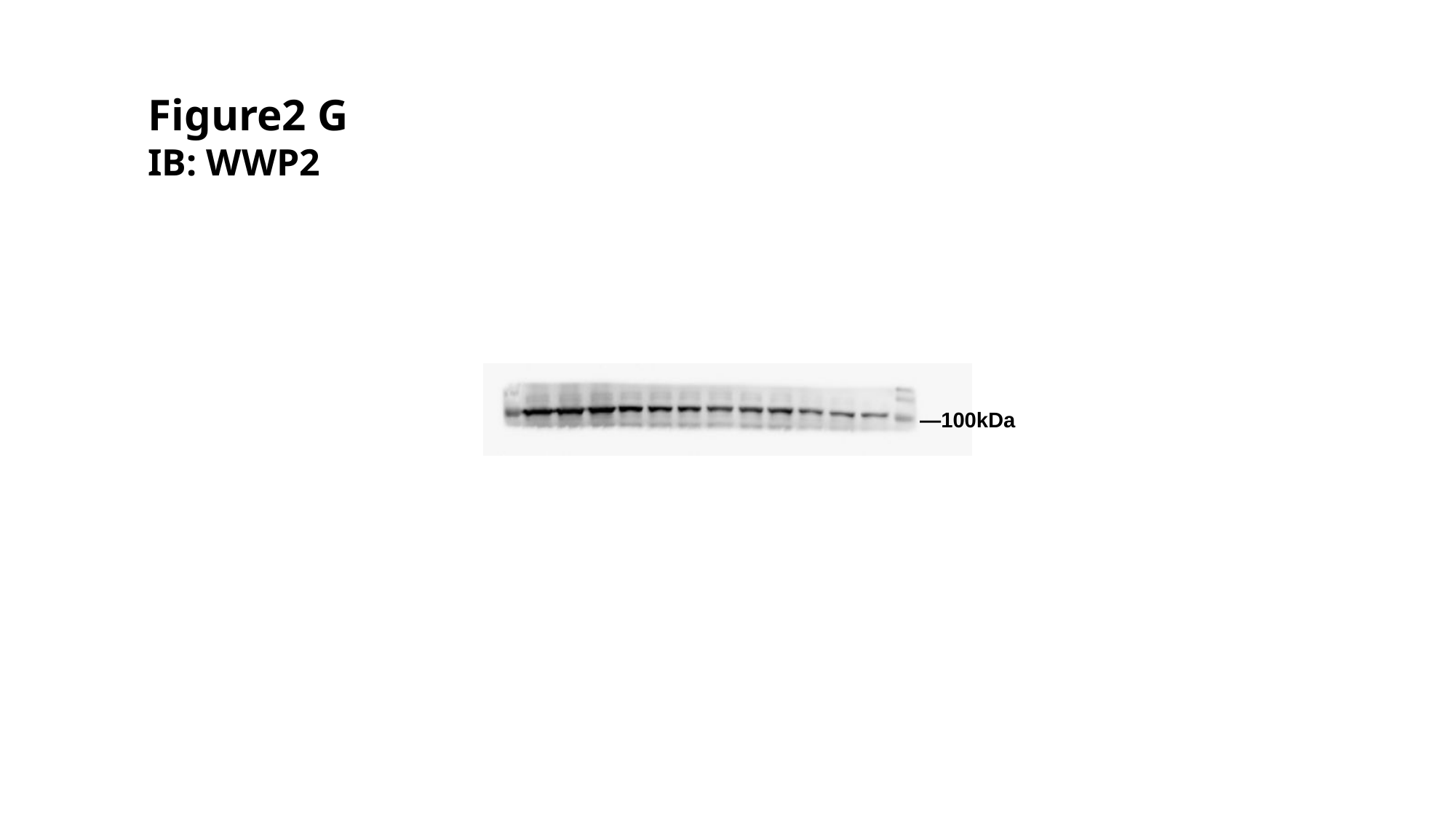

Figure2 G
IB: WWP2
—100kDa

## Slide 8
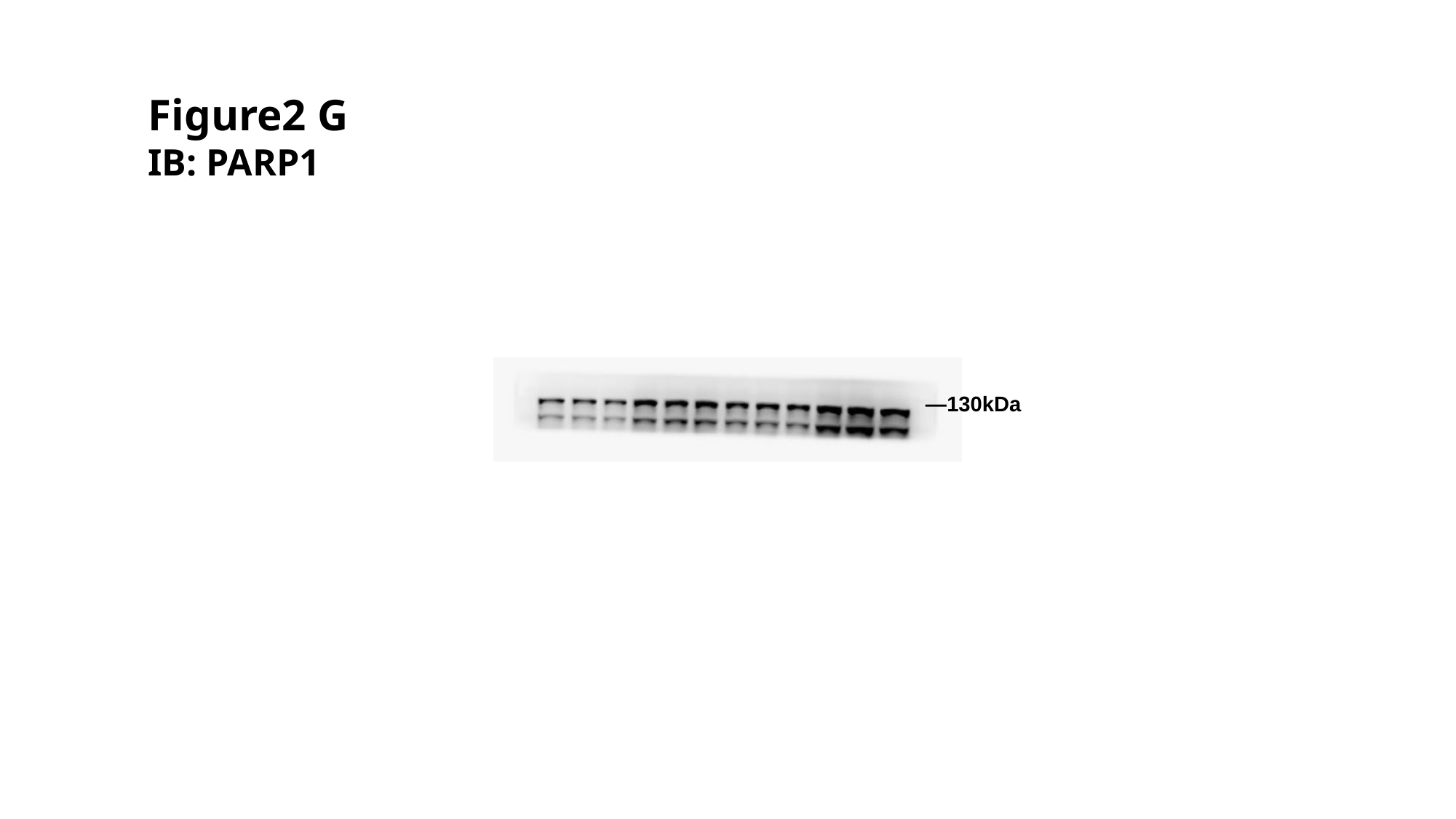

Figure2 G
IB: PARP1
—130kDa

## Slide 9
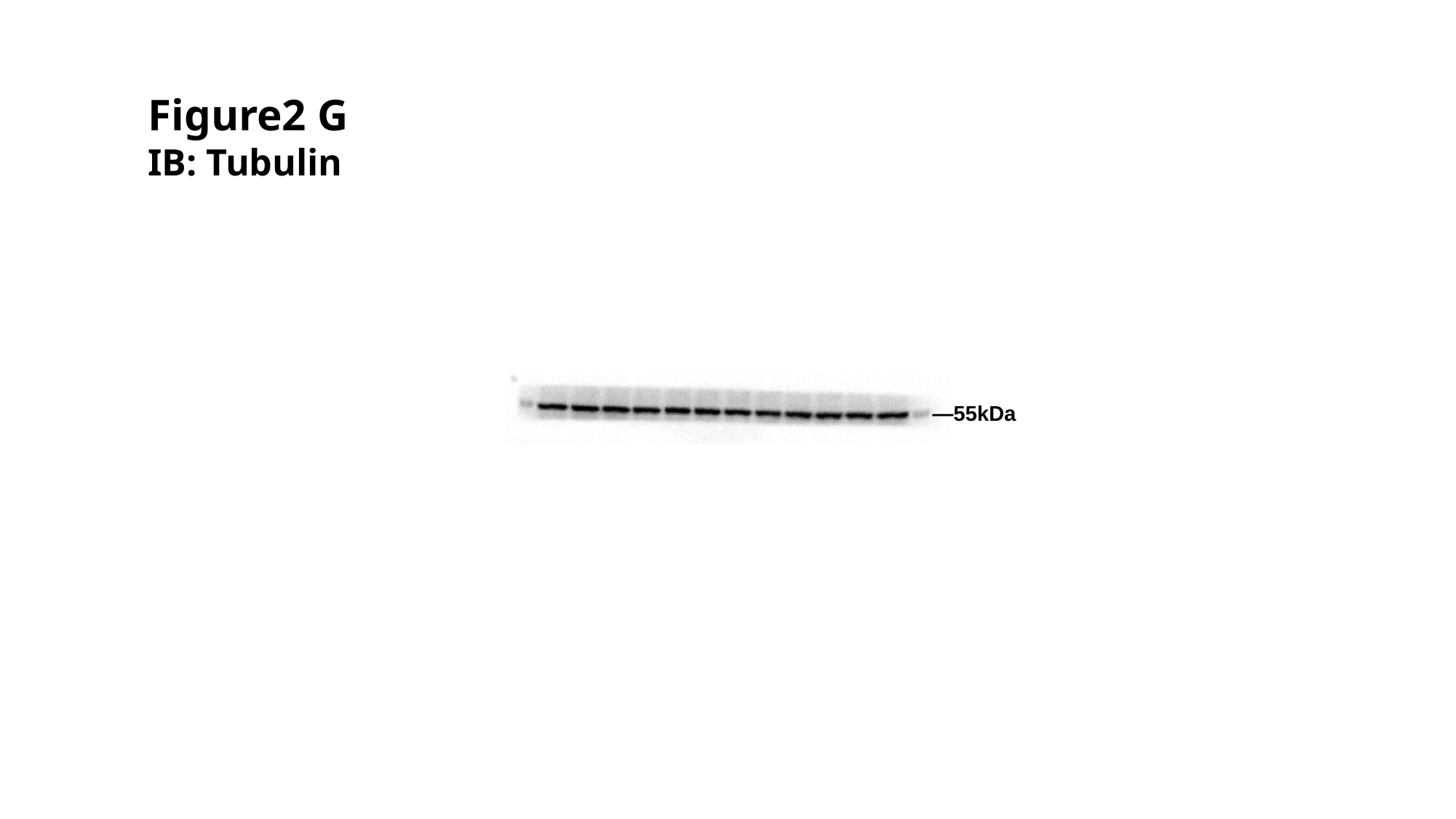

Figure2 G
IB: Tubulin
—55kDa

## Slide 10
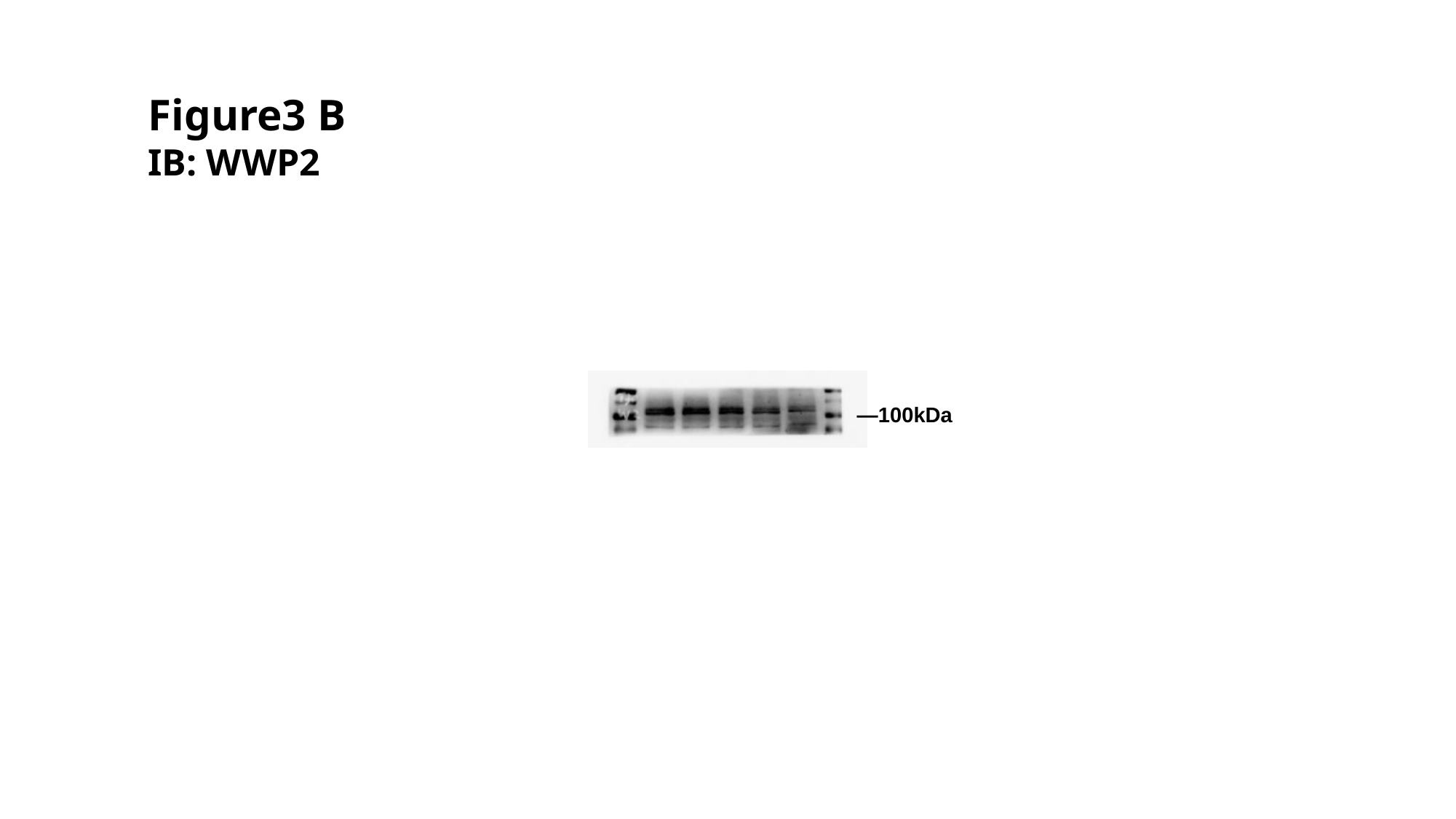

Figure3 B
IB: WWP2
—100kDa

## Slide 11
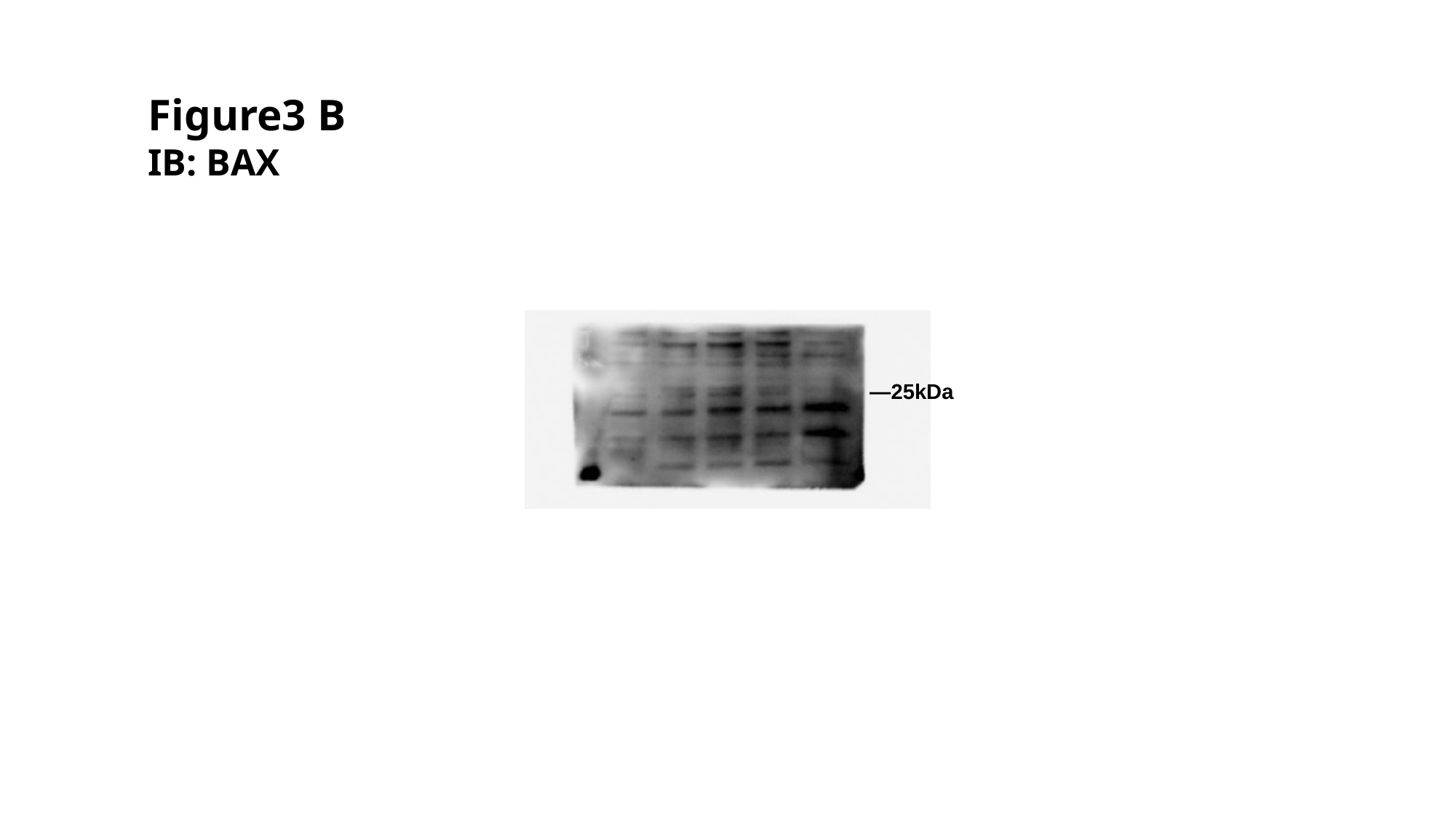

Figure3 B
IB: BAX
—25kDa

## Slide 12
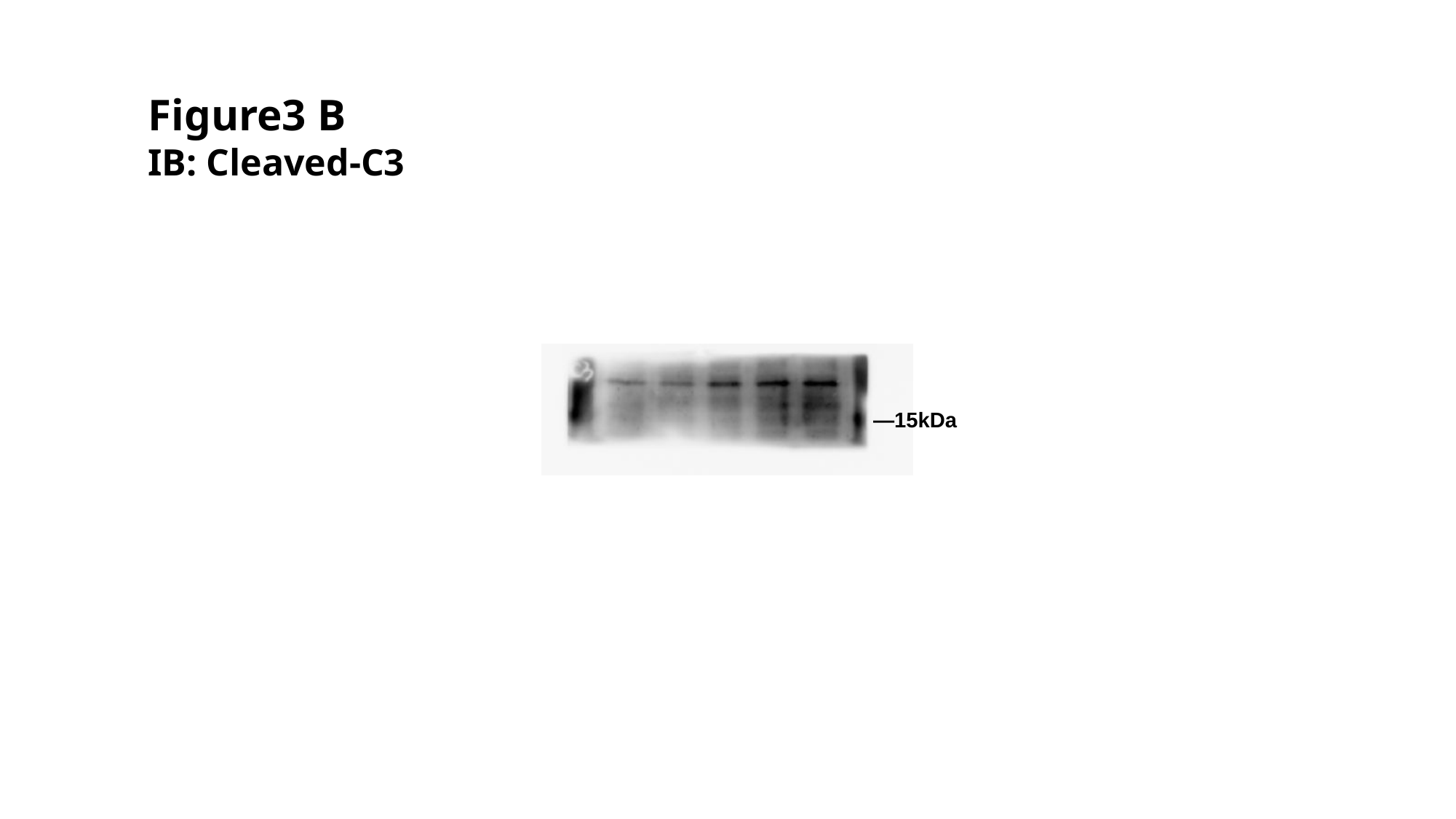

Figure3 B
IB: Cleaved-C3
—15kDa

## Slide 13
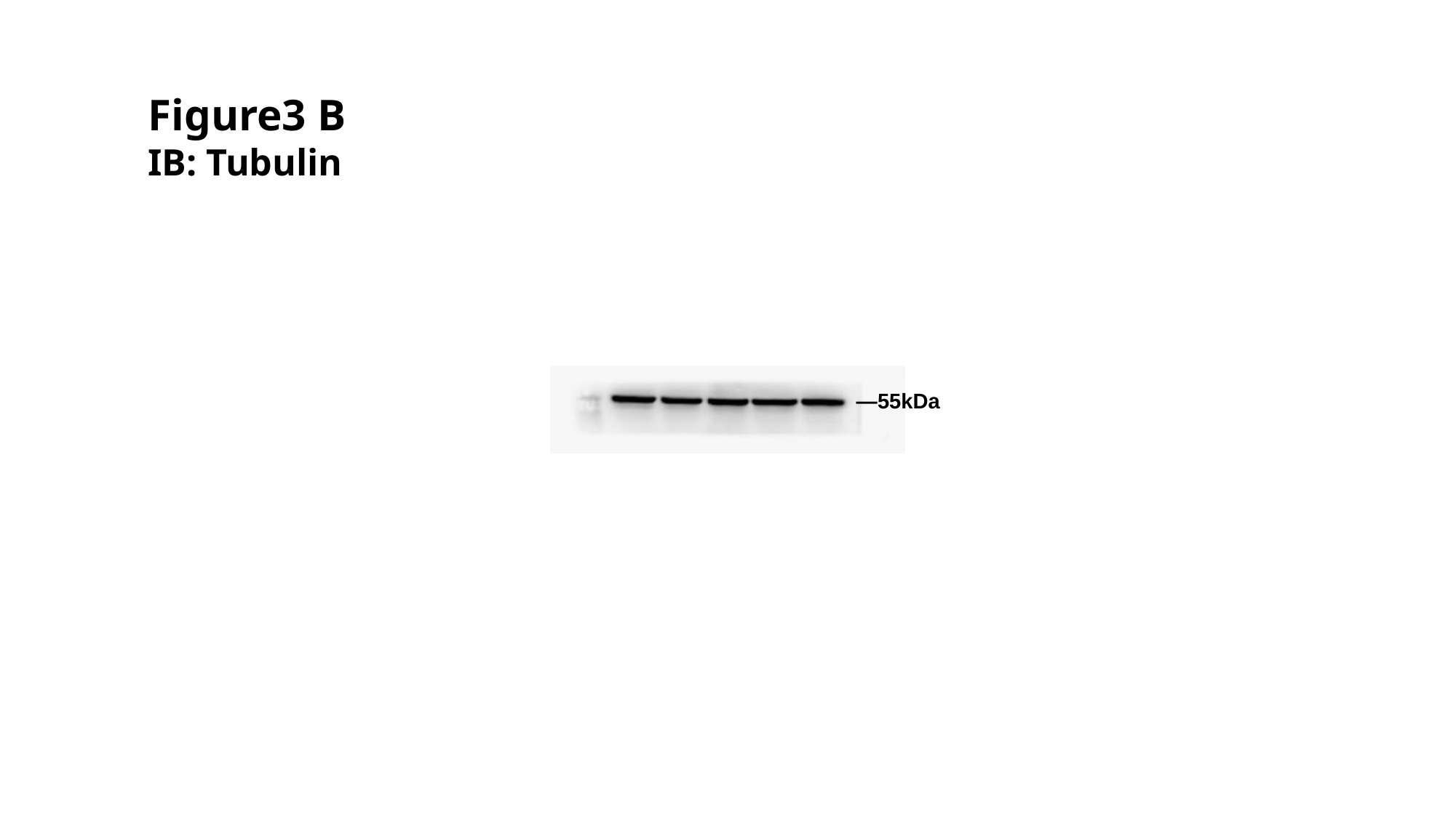

Figure3 B
IB: Tubulin
—55kDa

## Slide 14
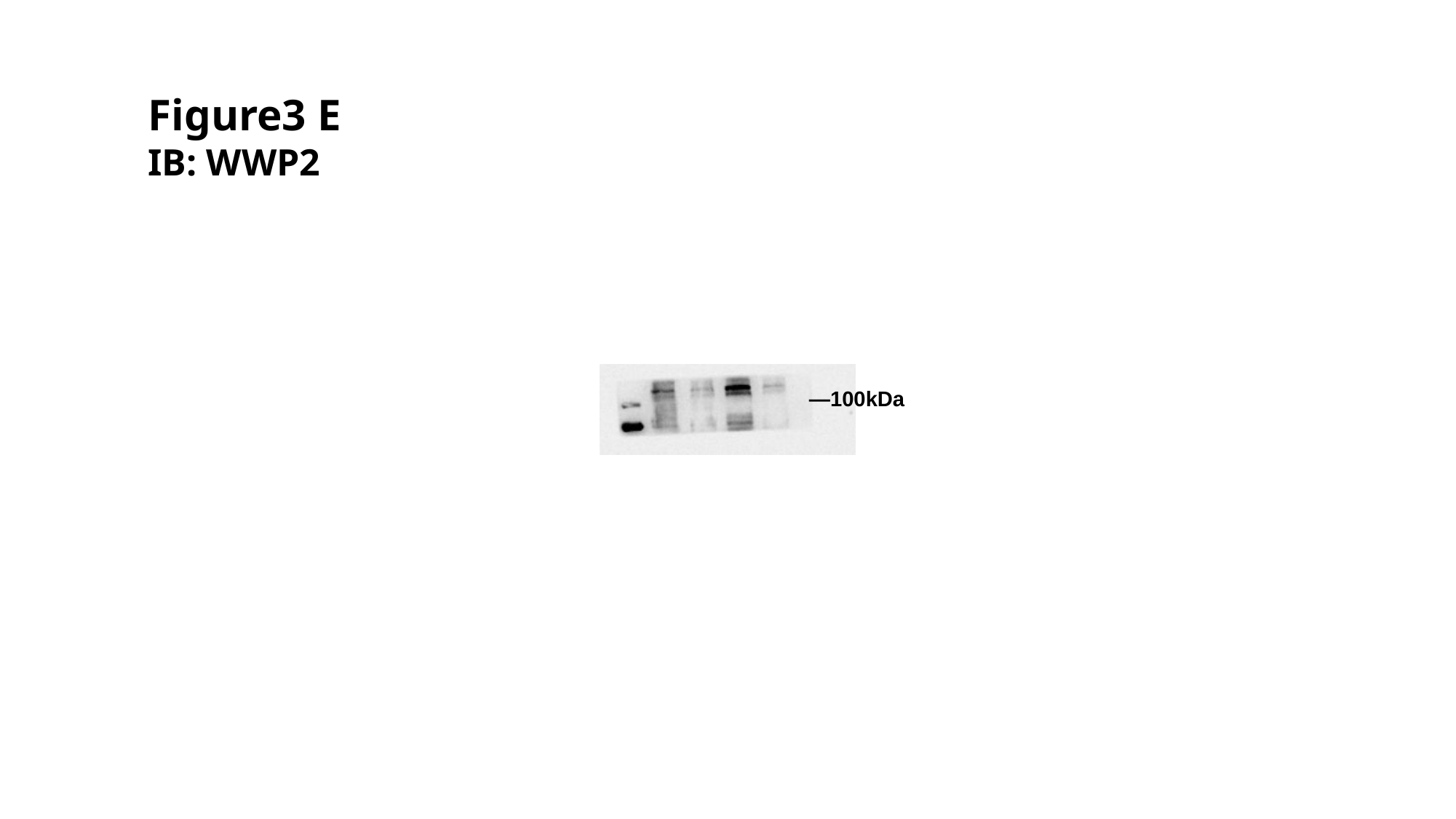

Figure3 E
IB: WWP2
—100kDa

## Slide 15
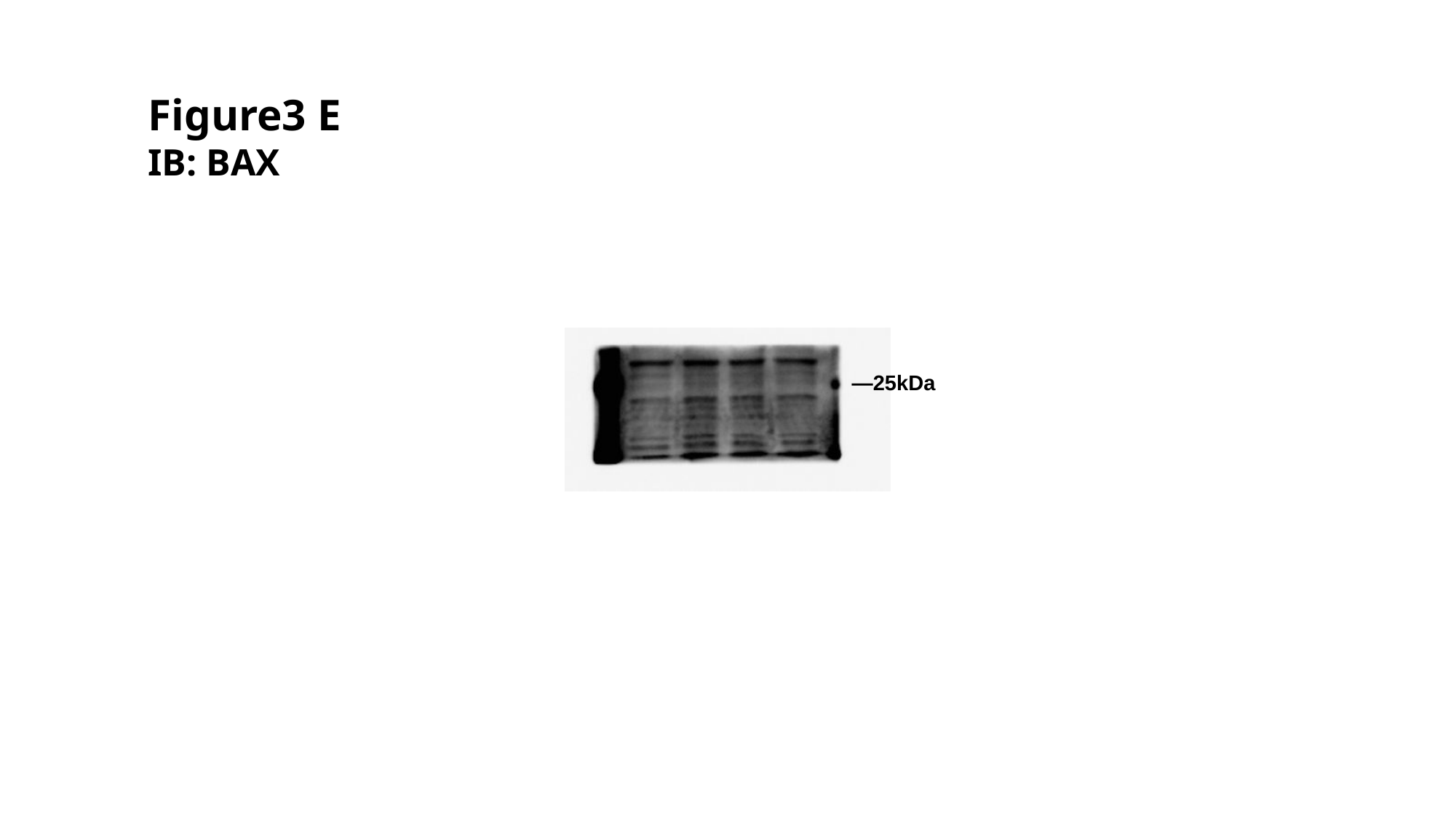

Figure3 E
IB: BAX
—25kDa

## Slide 16
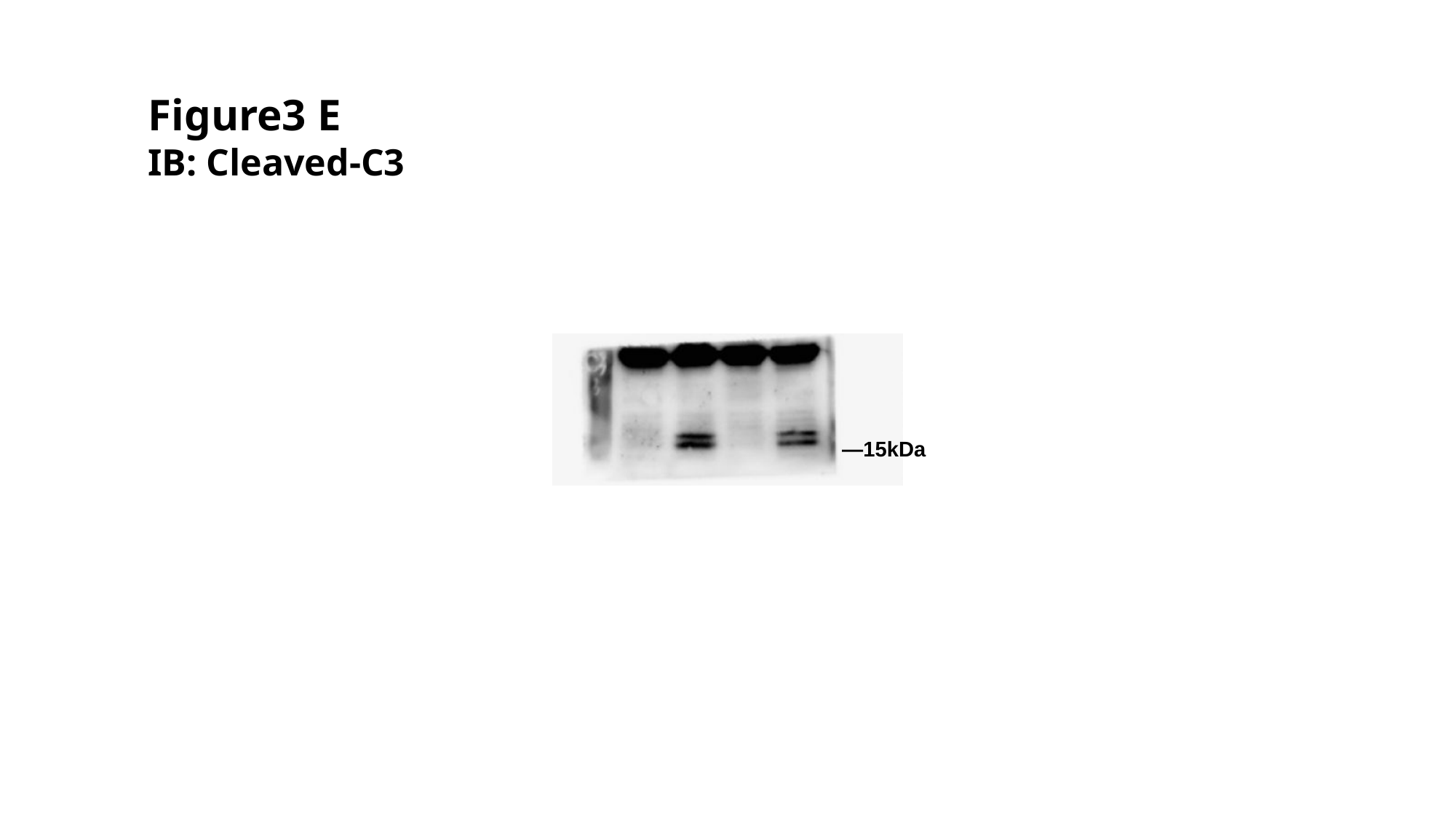

Figure3 E
IB: Cleaved-C3
—15kDa

## Slide 17
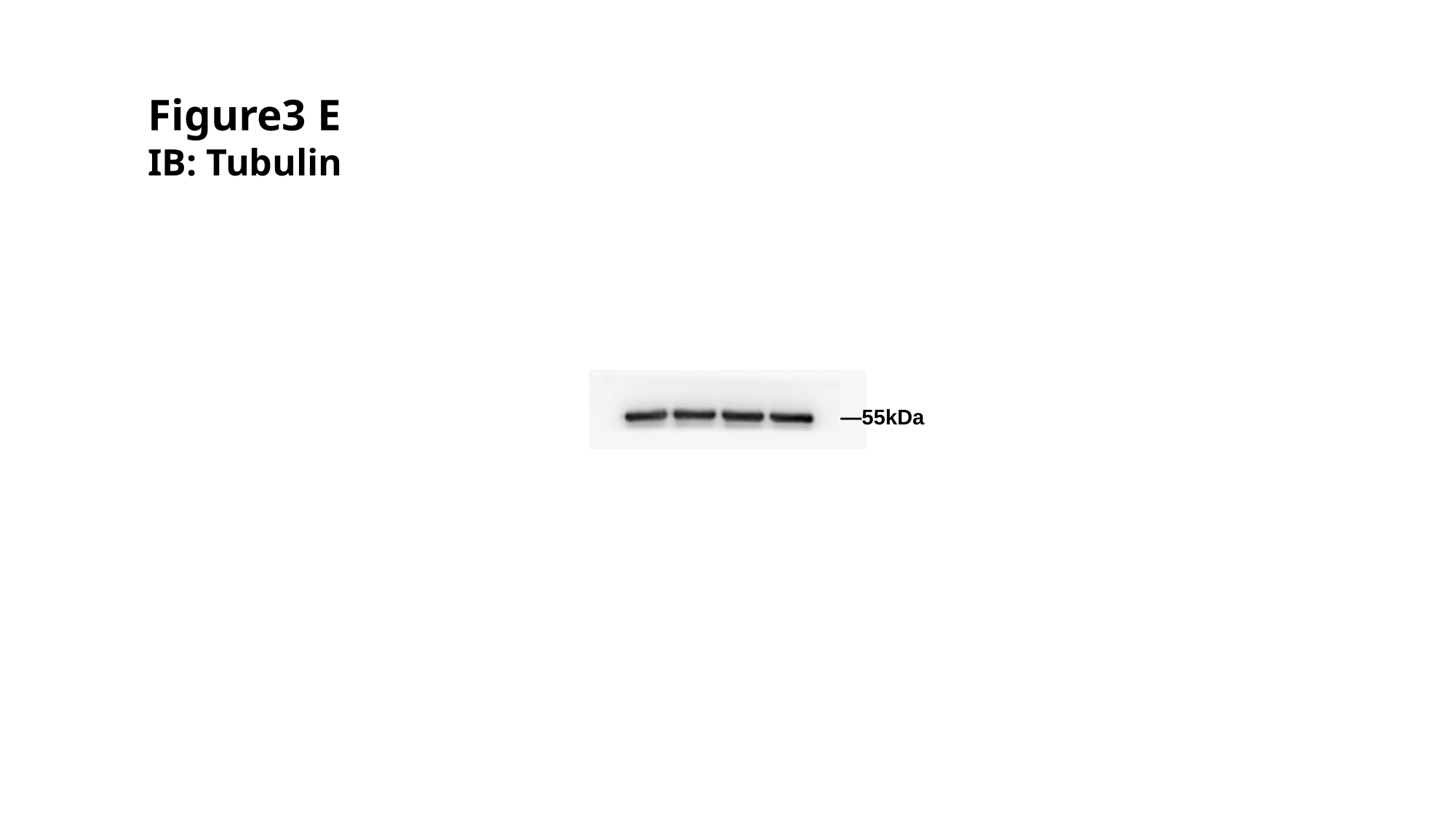

Figure3 E
IB: Tubulin
—55kDa

## Slide 18
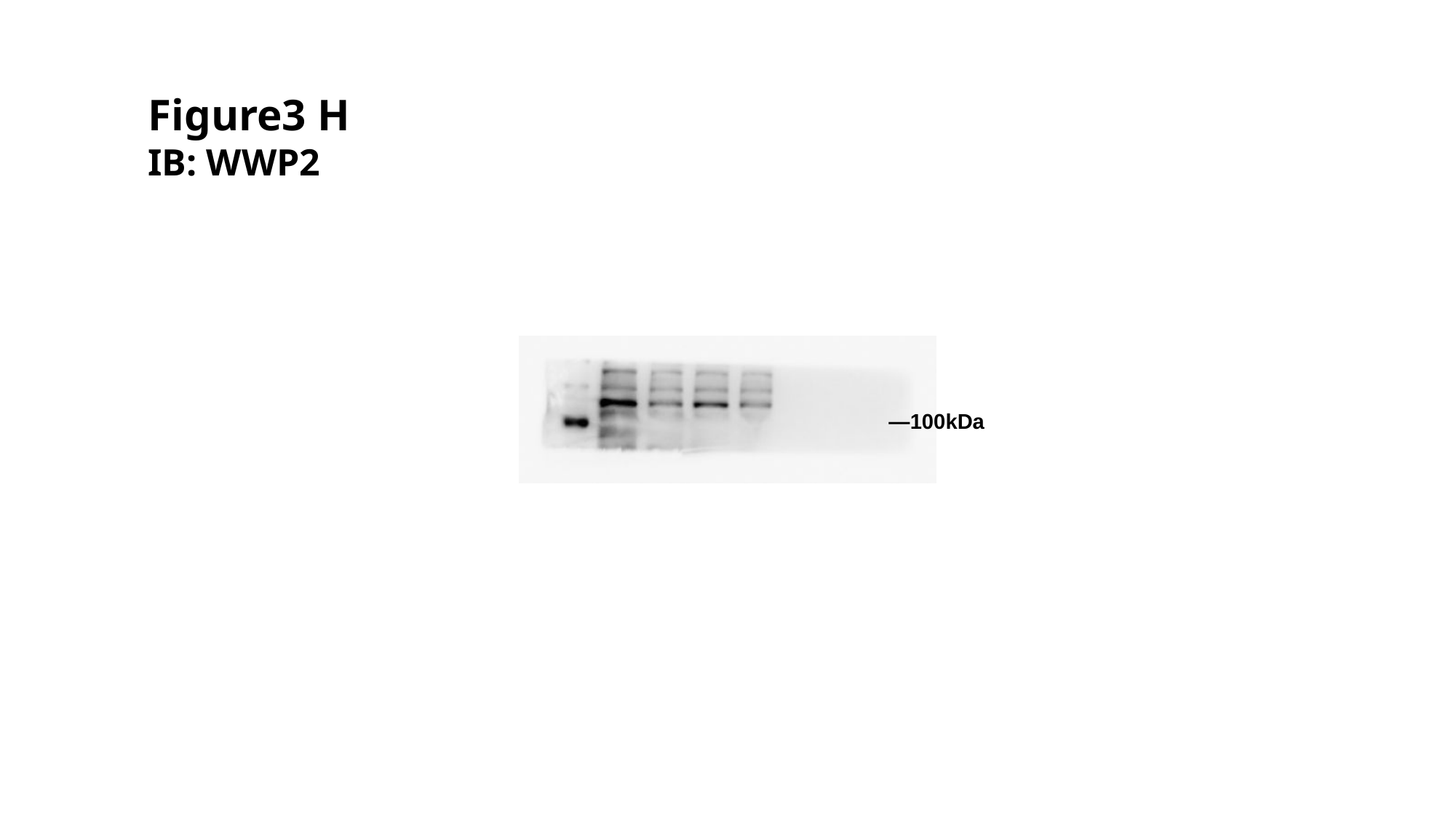

Figure3 H
IB: WWP2
—100kDa

## Slide 19
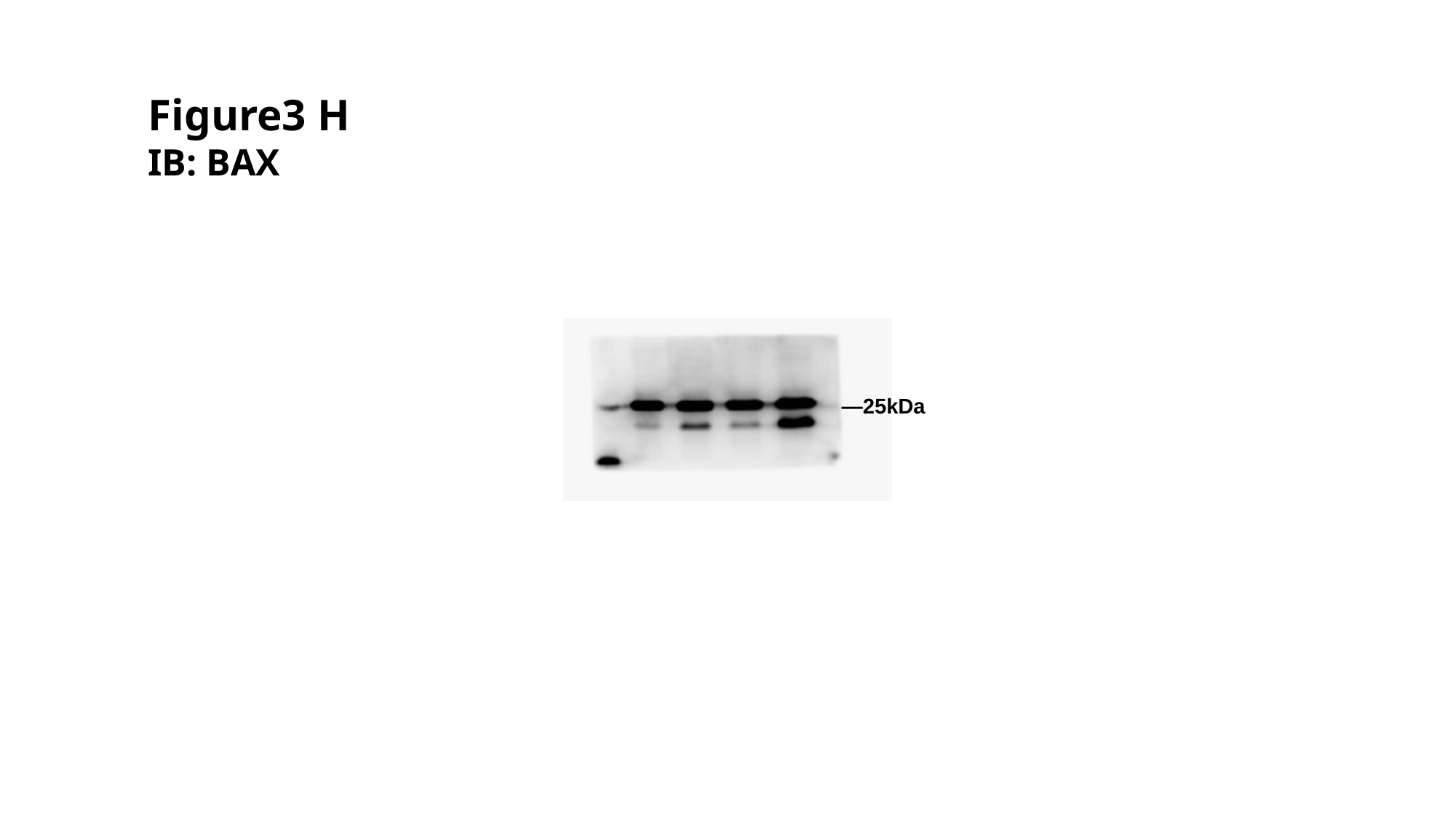

Figure3 H
IB: BAX
—25kDa

## Slide 20
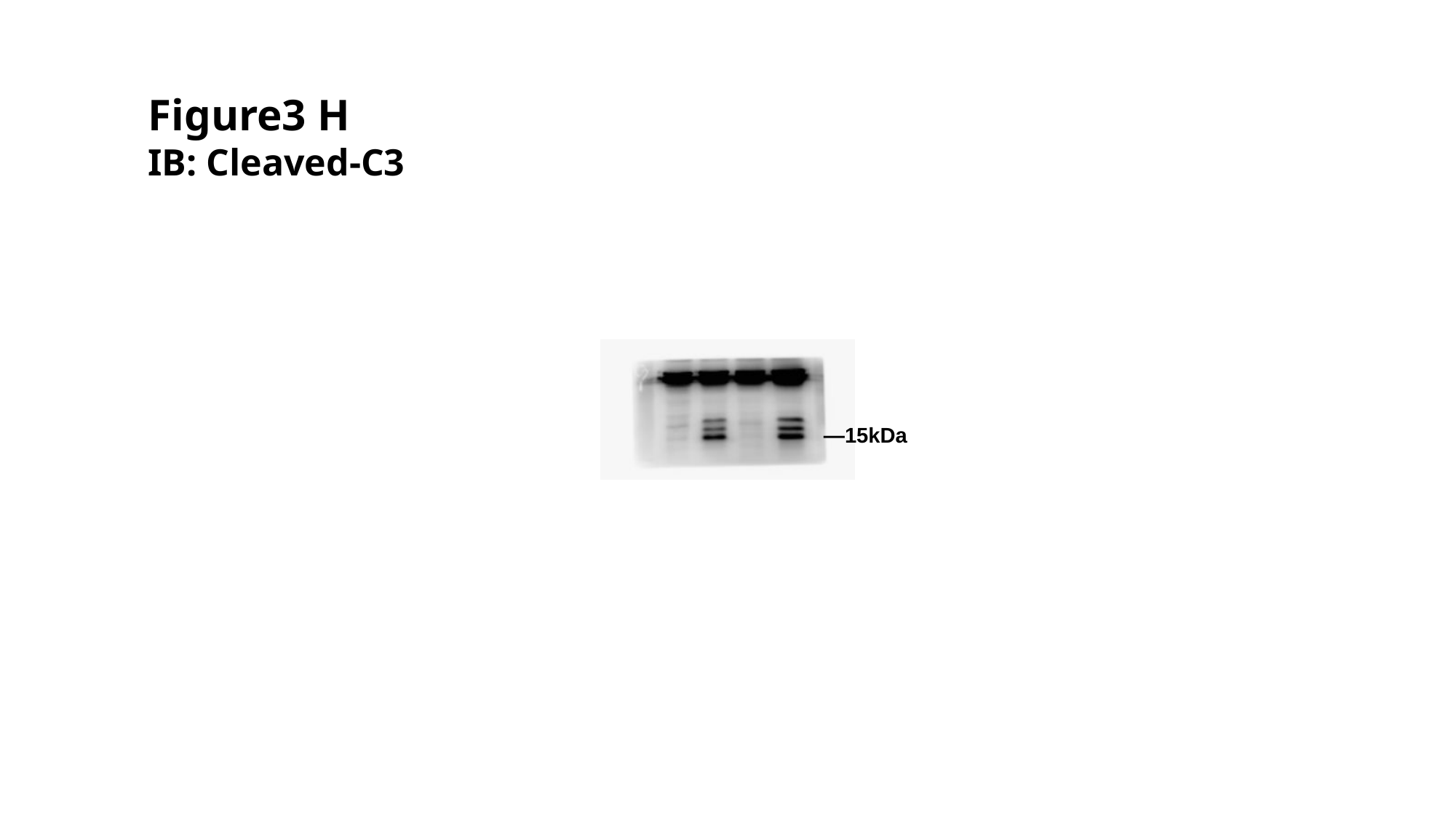

Figure3 H
IB: Cleaved-C3
—15kDa

## Slide 21
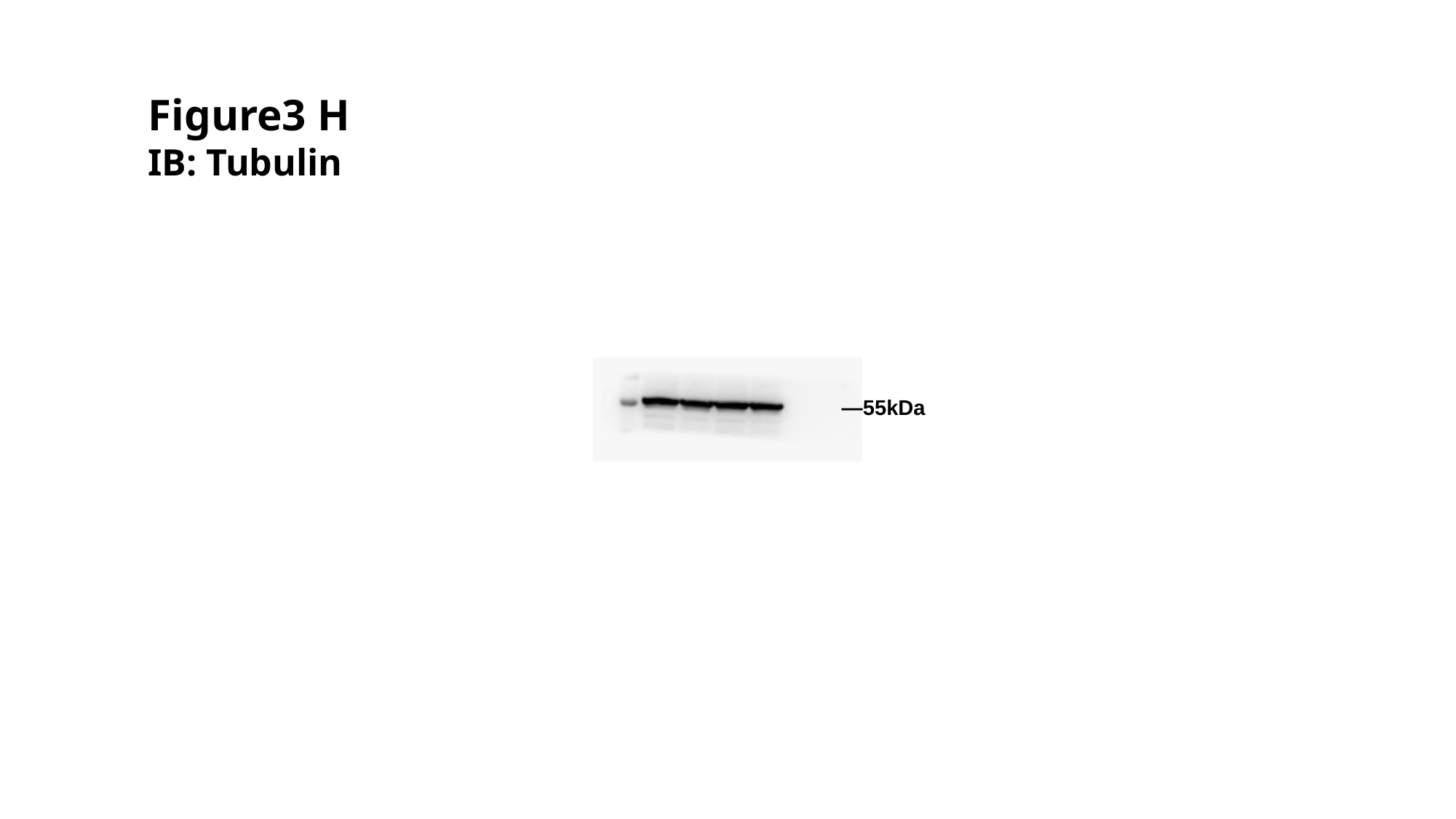

Figure3 H
IB: Tubulin
—55kDa

## Slide 22
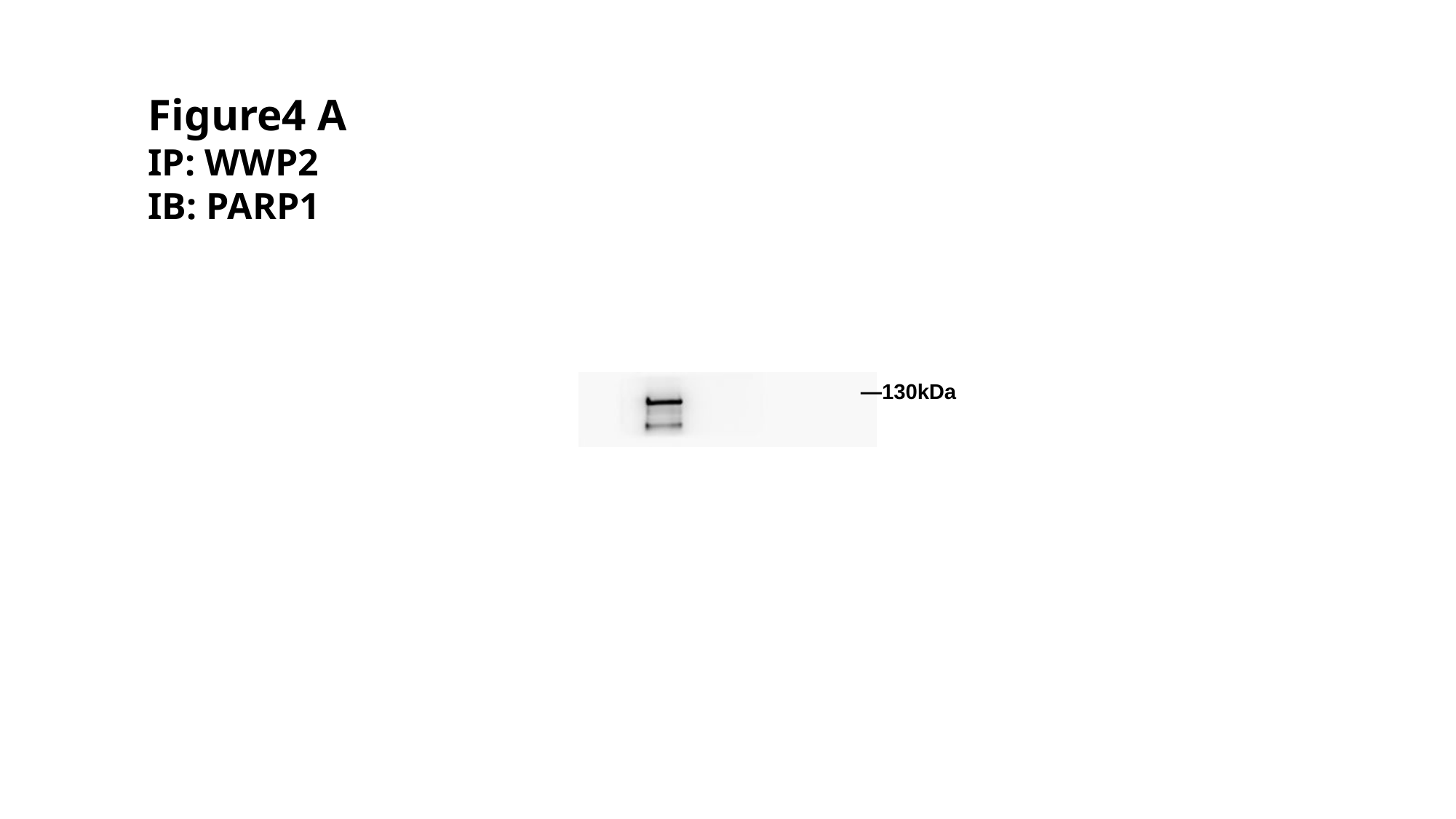

Figure4 A
IP: WWP2
IB: PARP1
—130kDa

## Slide 23
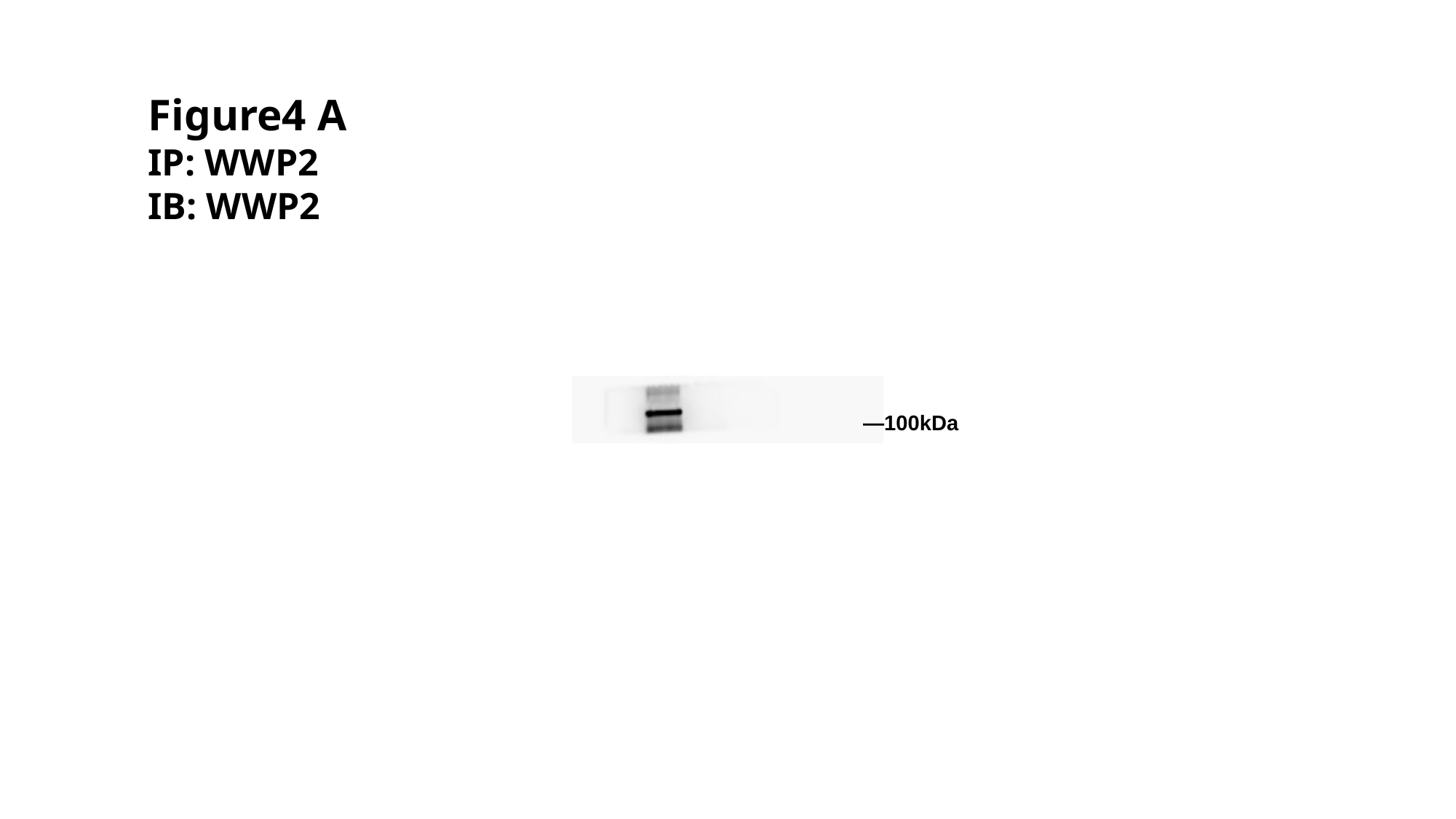

Figure4 A
IP: WWP2
IB: WWP2
—100kDa

## Slide 24
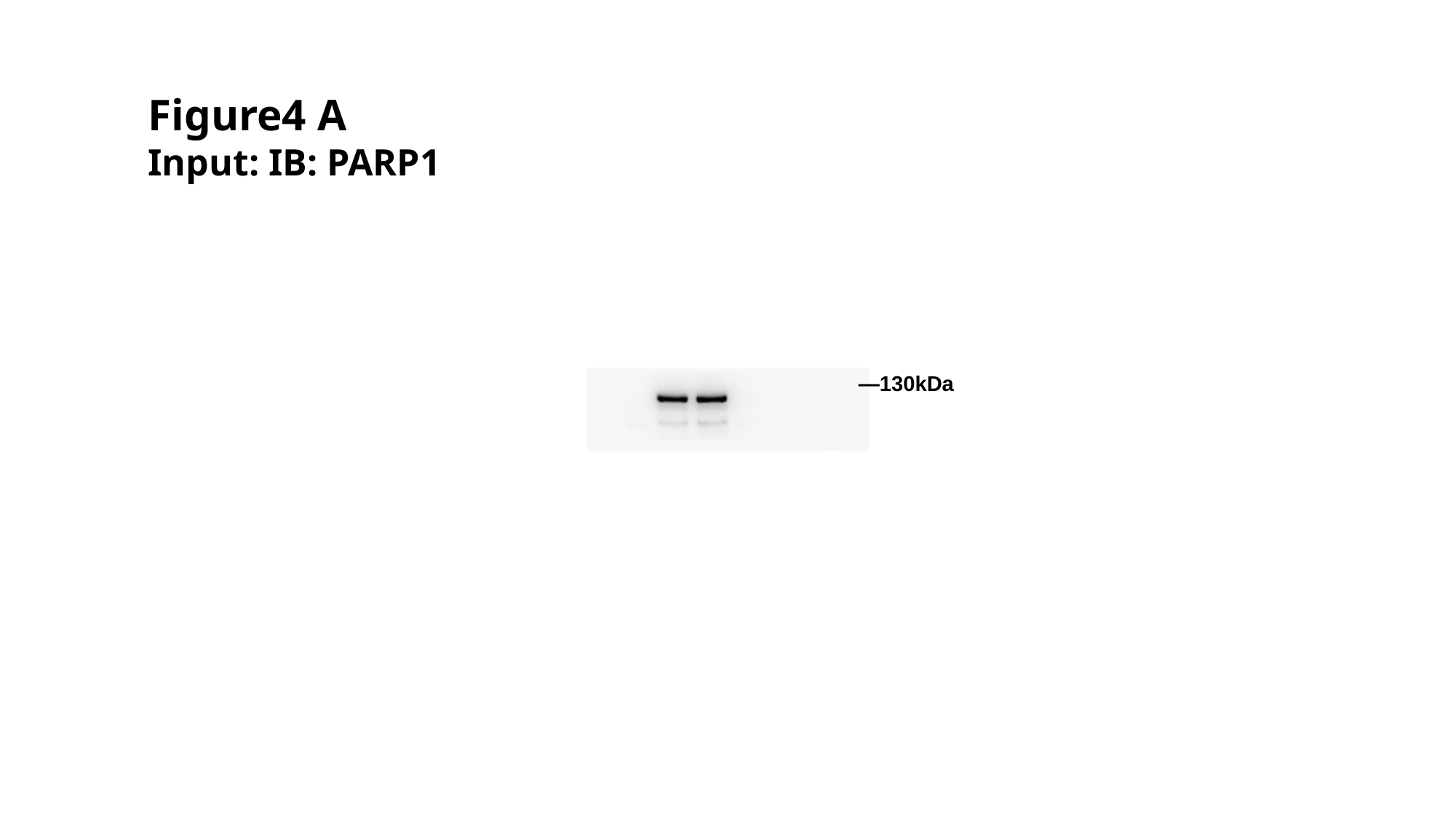

Figure4 A
Input: IB: PARP1
—130kDa

## Slide 25
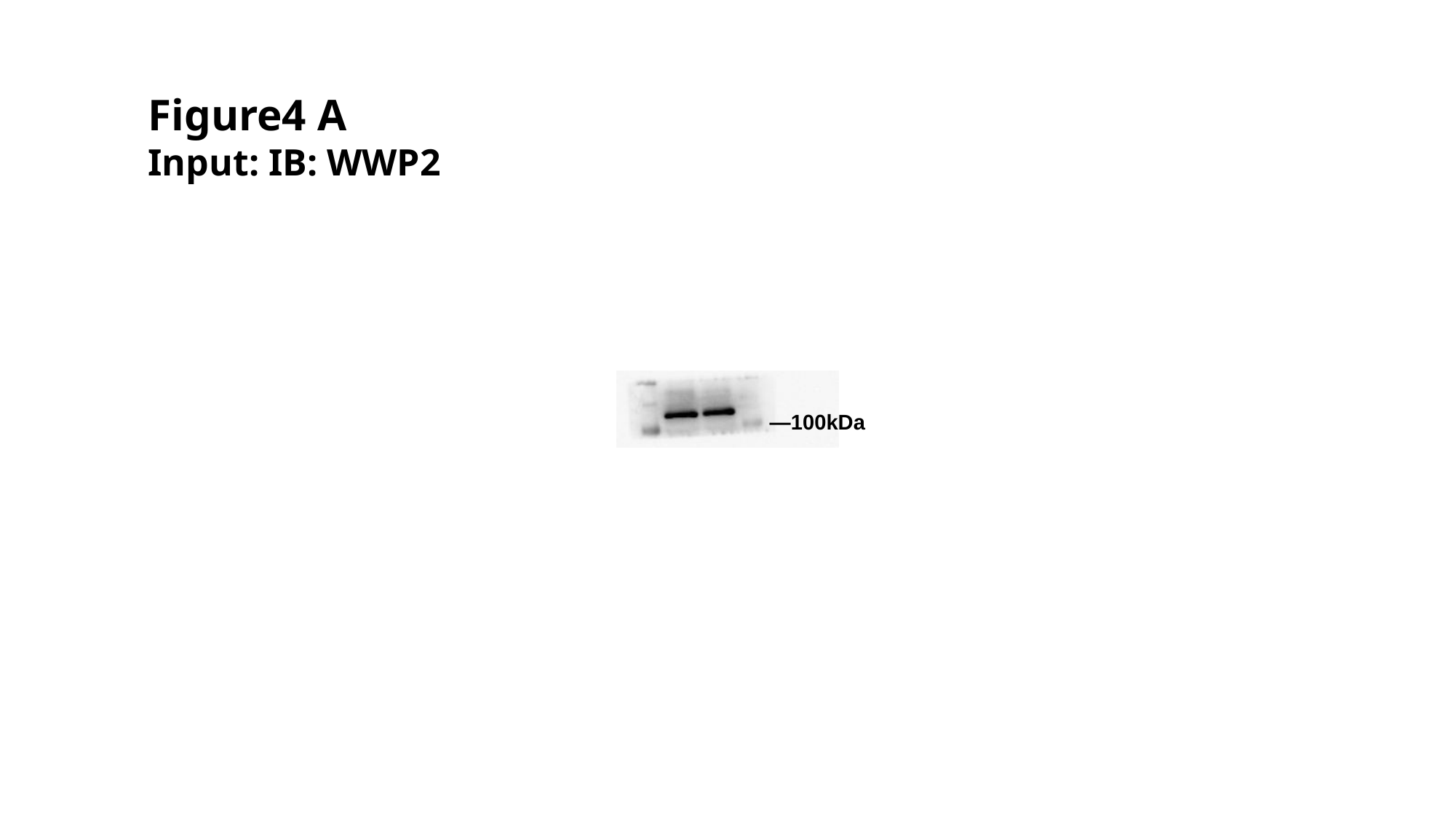

Figure4 A
Input: IB: WWP2
—100kDa

## Slide 26
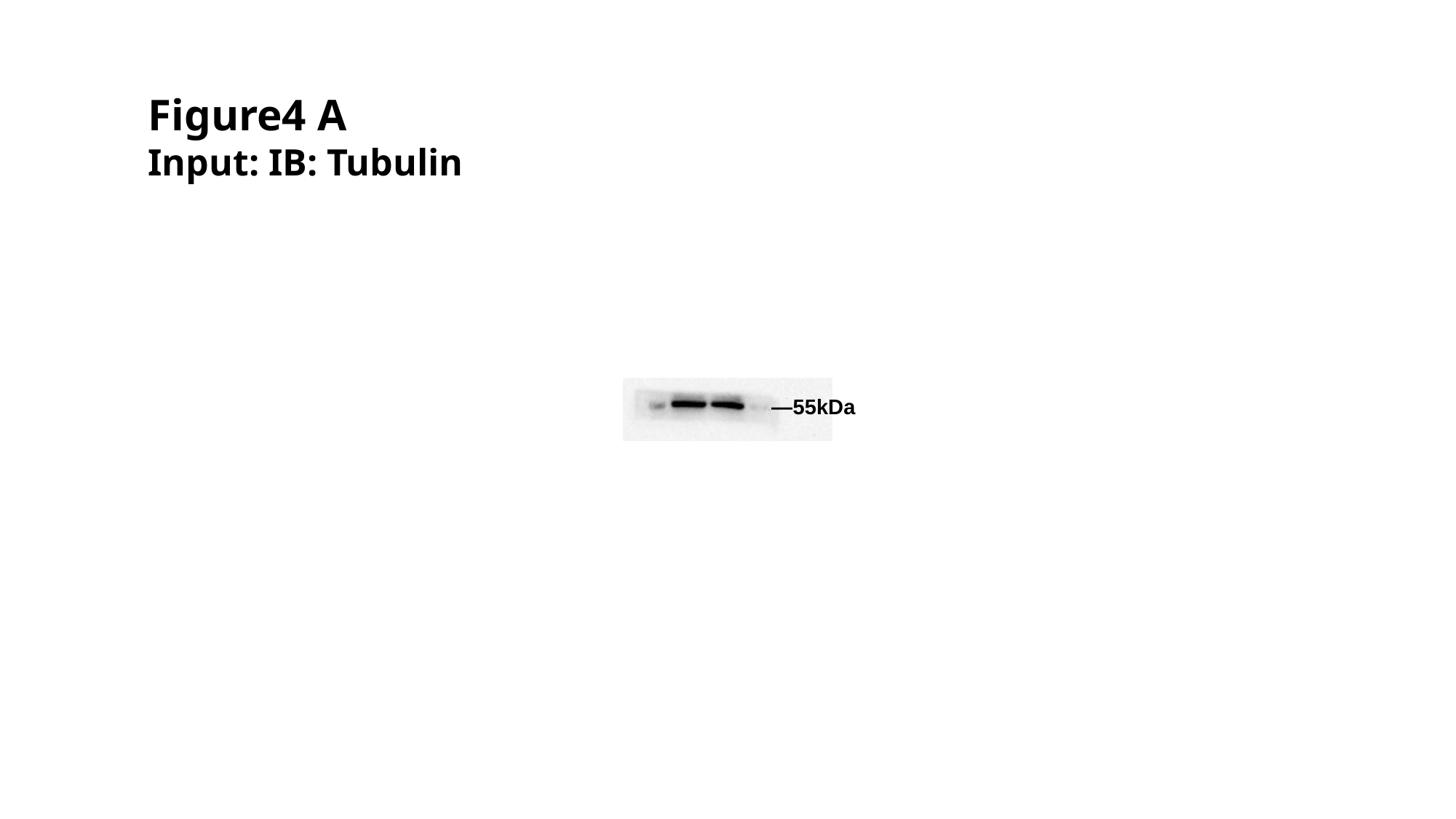

Figure4 A
Input: IB: Tubulin
—55kDa

## Slide 27
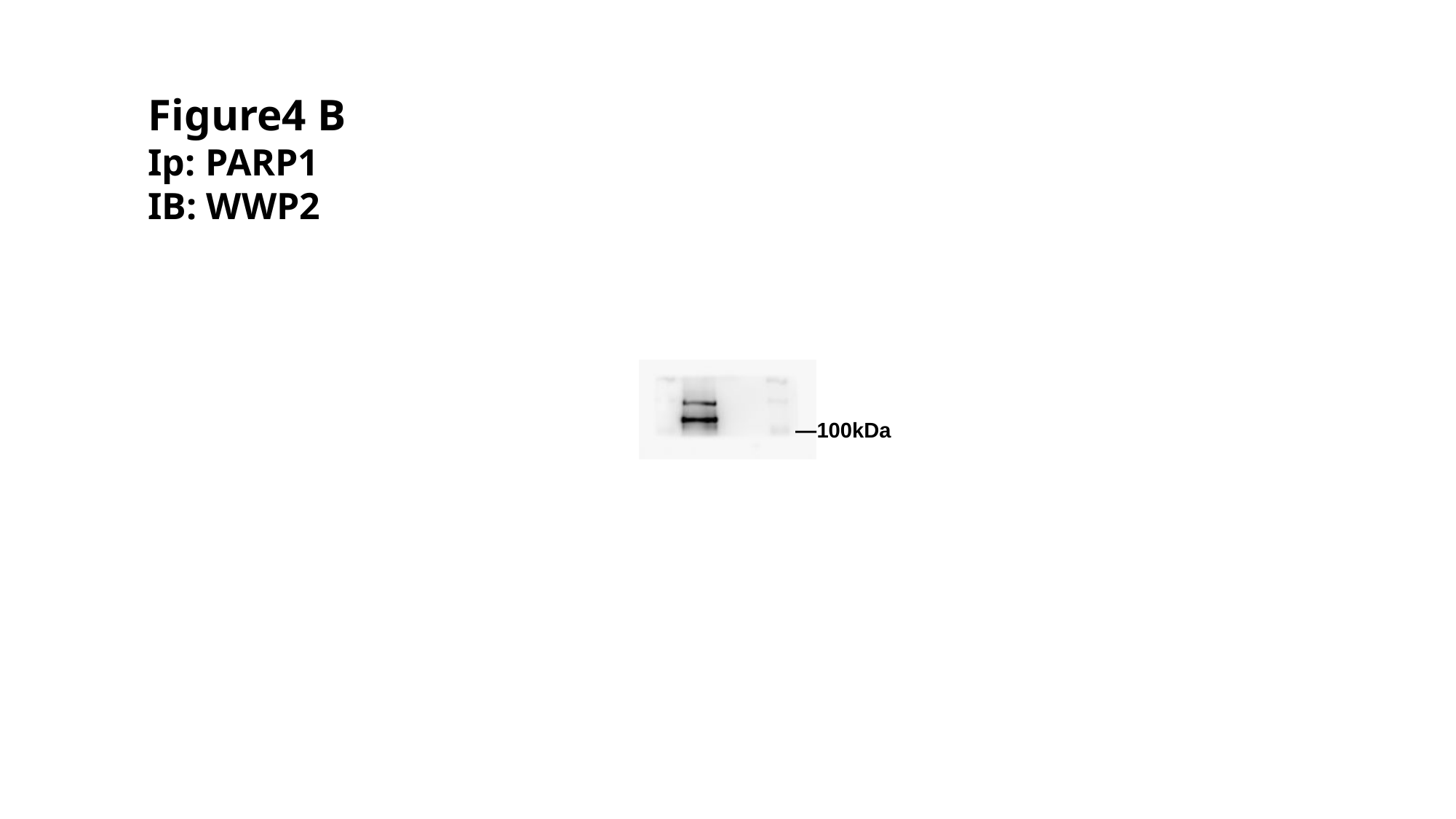

Figure4 B
Ip: PARP1
IB: WWP2
—100kDa

## Slide 28
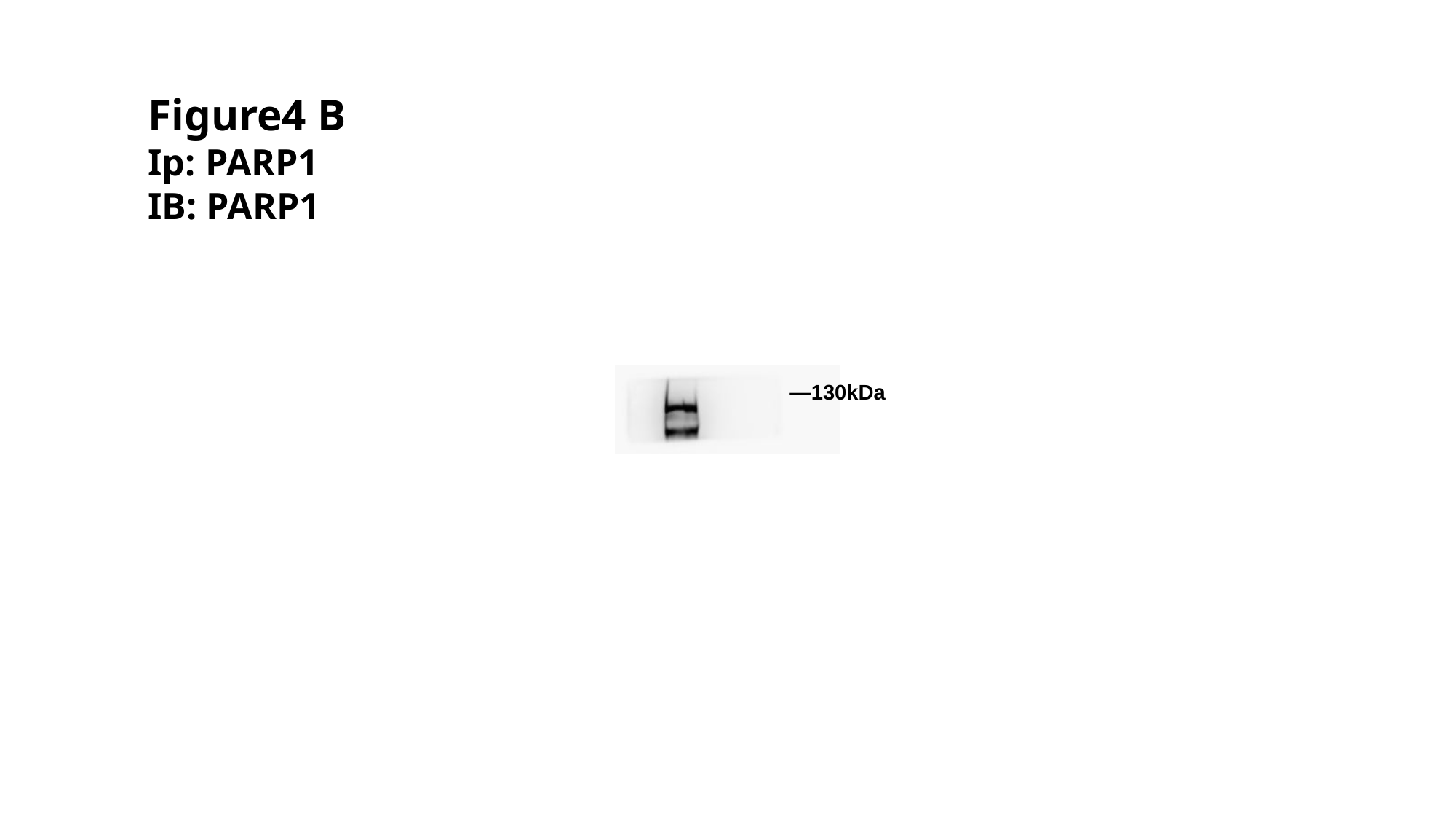

Figure4 B
Ip: PARP1
IB: PARP1
—130kDa

## Slide 29
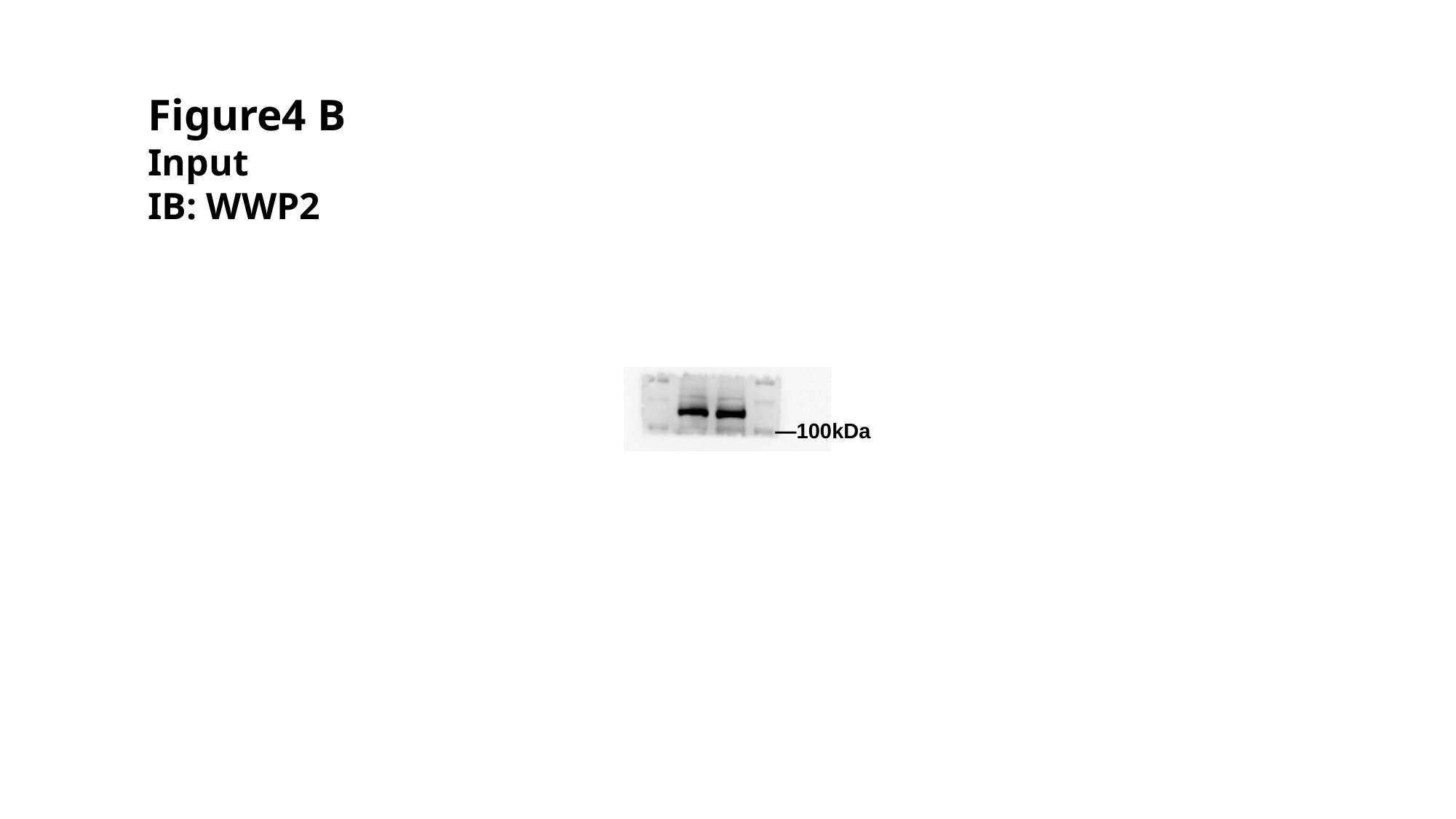

Figure4 B
Input
IB: WWP2
—100kDa

## Slide 30
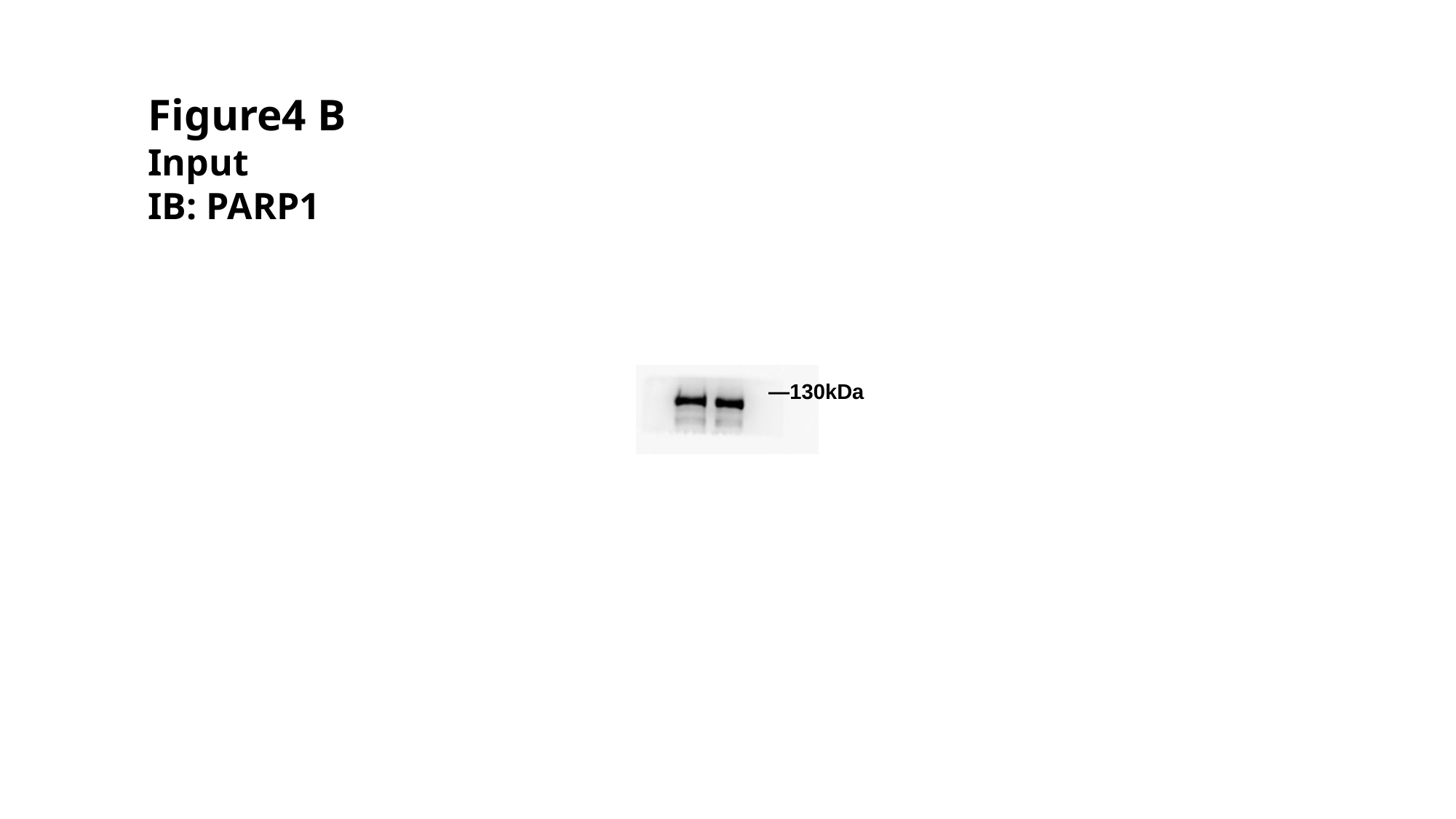

Figure4 B
Input
IB: PARP1
—130kDa

## Slide 31
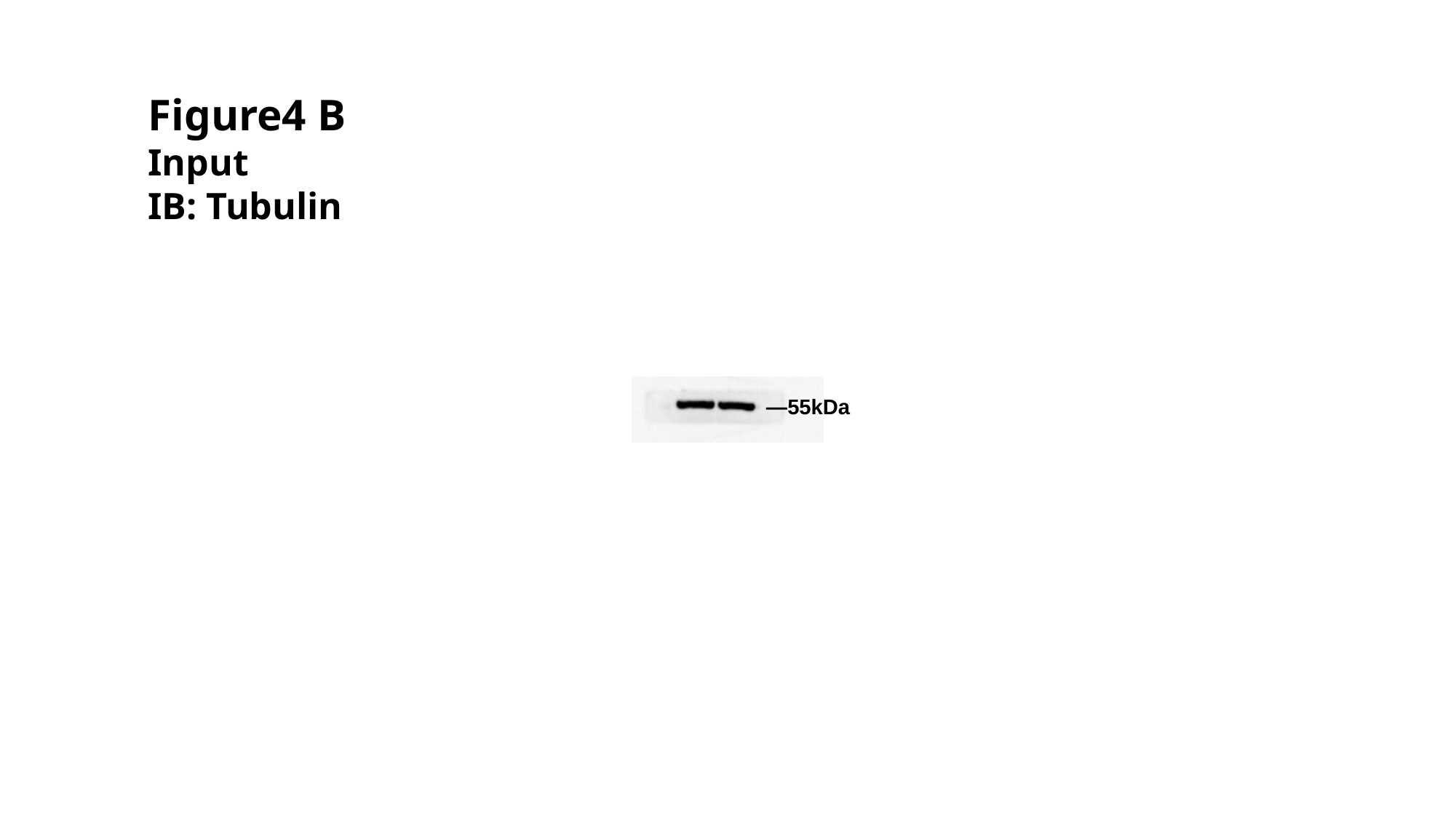

Figure4 B
Input
IB: Tubulin
—55kDa

## Slide 32
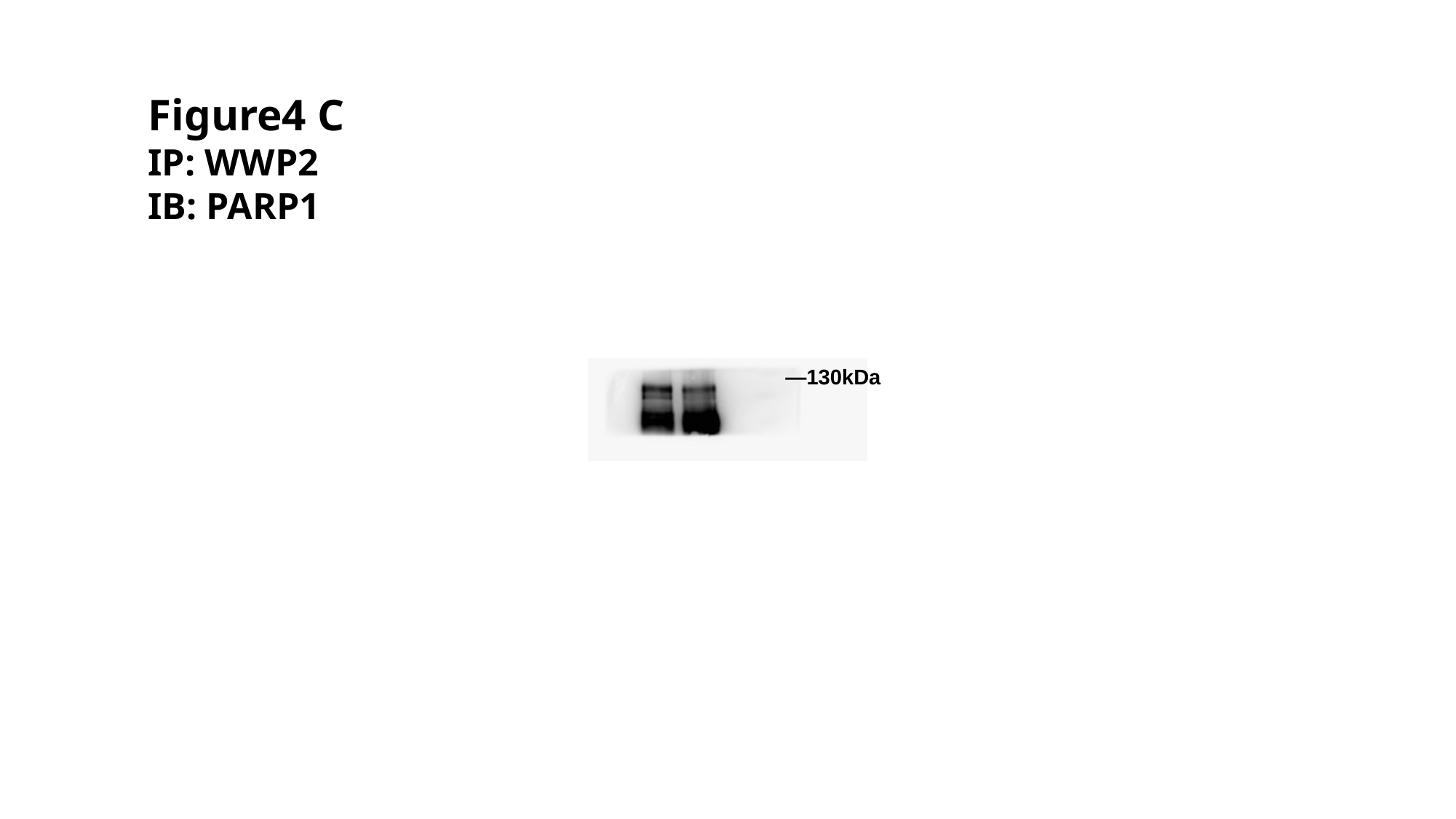

Figure4 C
IP: WWP2
IB: PARP1
—130kDa

## Slide 33
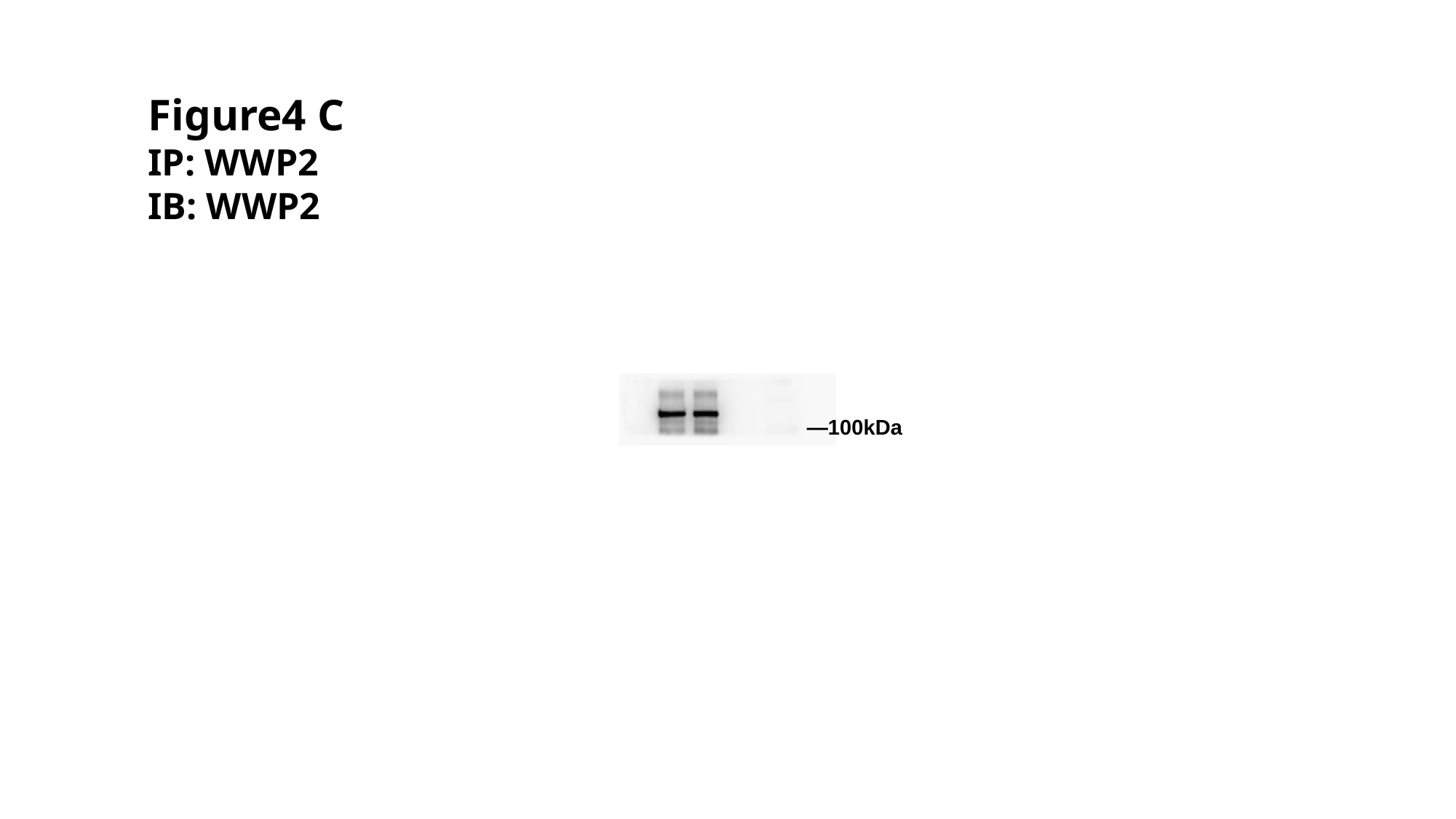

Figure4 C
IP: WWP2
IB: WWP2
—100kDa

## Slide 34
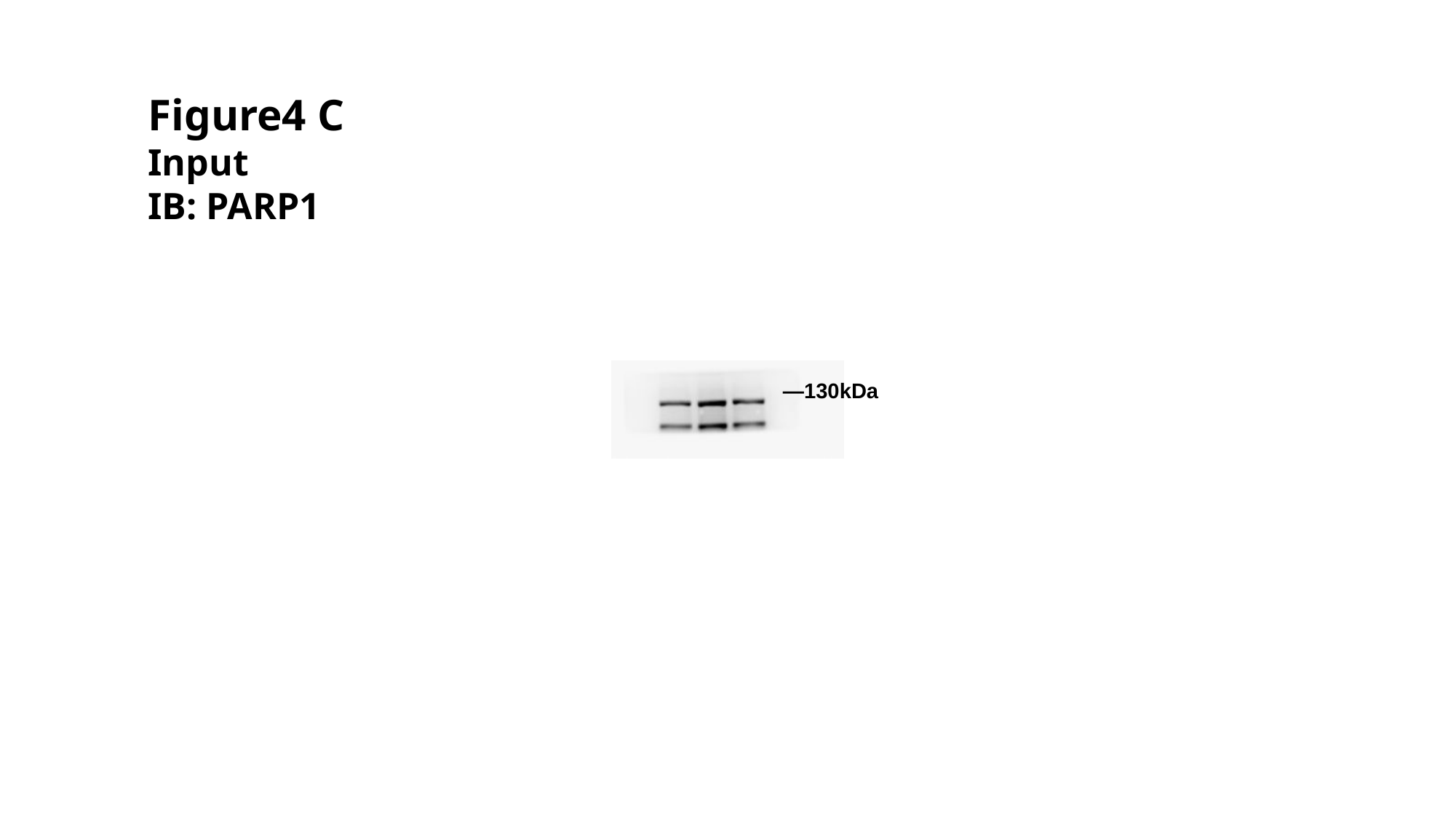

Figure4 C
Input
IB: PARP1
—130kDa

## Slide 35
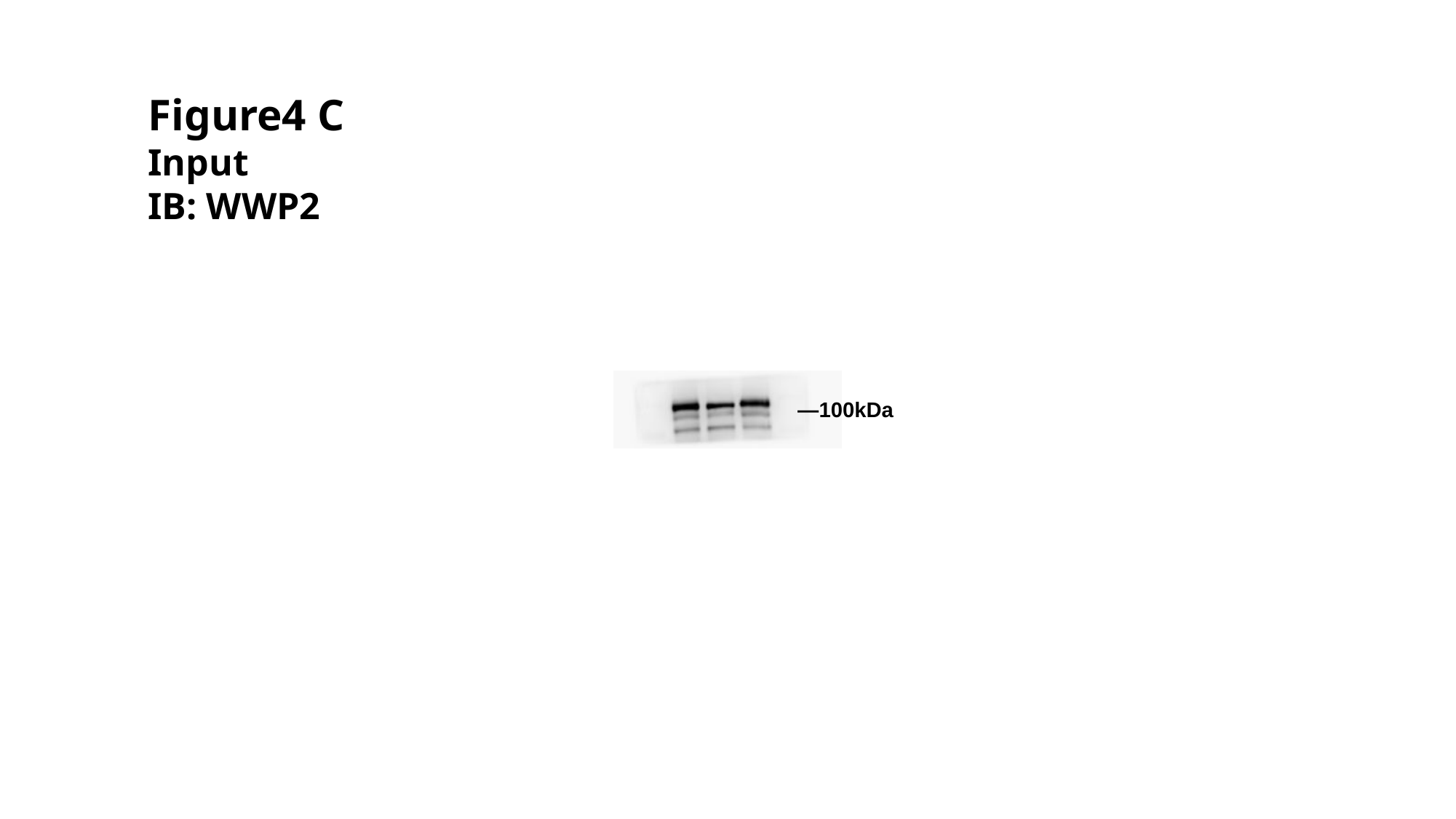

Figure4 C
Input
IB: WWP2
—100kDa

## Slide 36
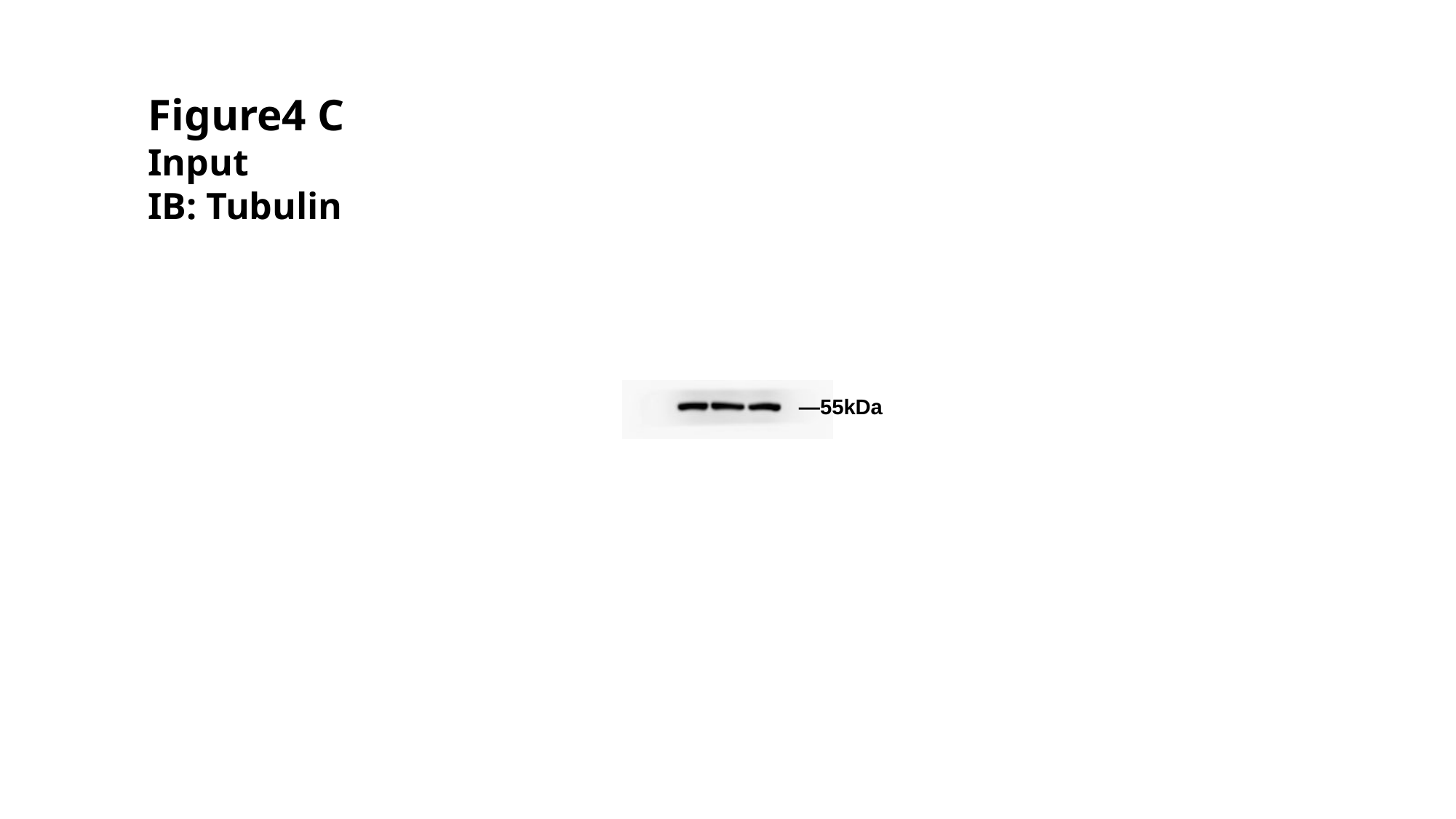

Figure4 C
Input
IB: Tubulin
—55kDa

## Slide 37
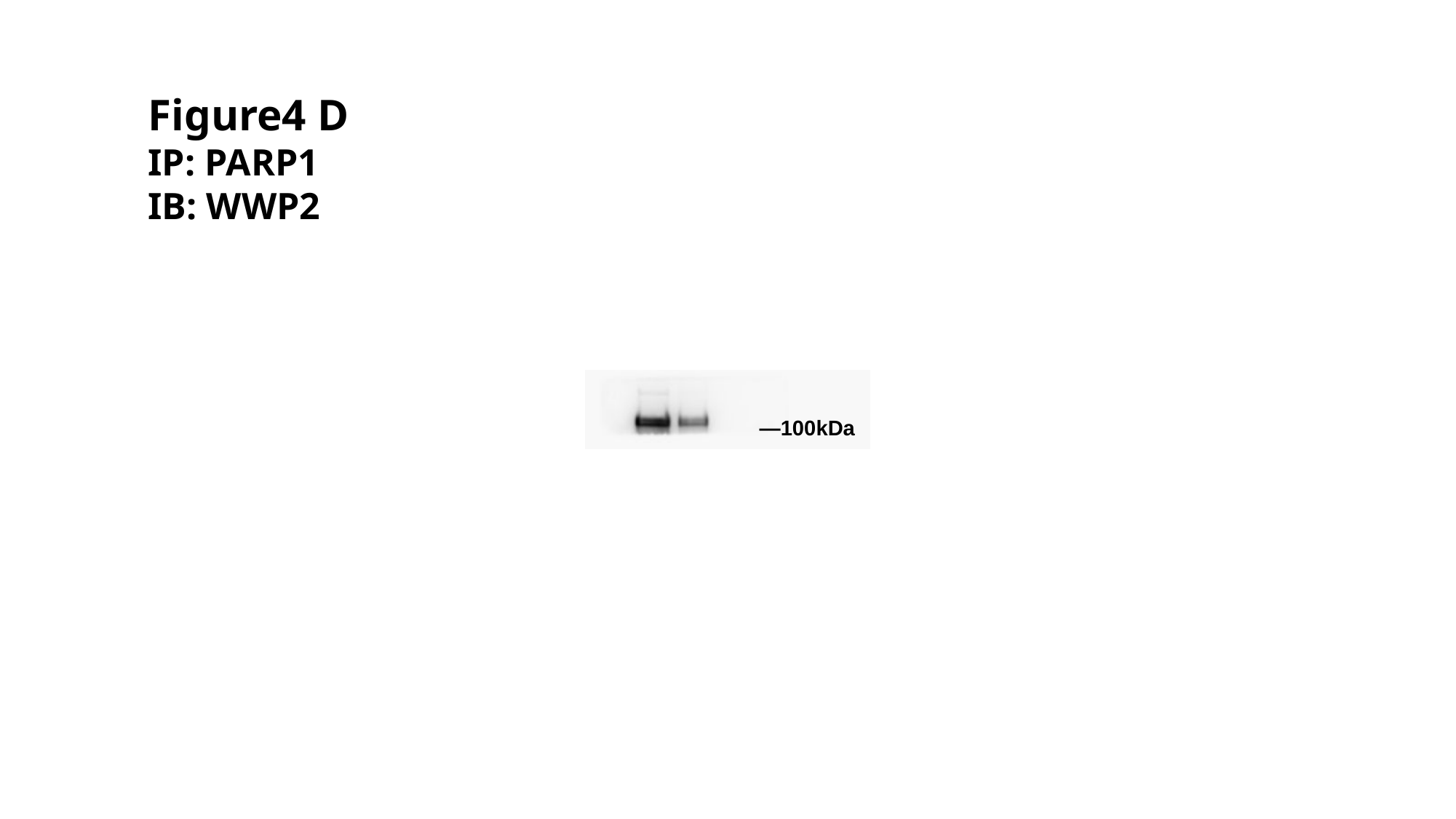

Figure4 D
IP: PARP1
IB: WWP2
—100kDa

## Slide 38
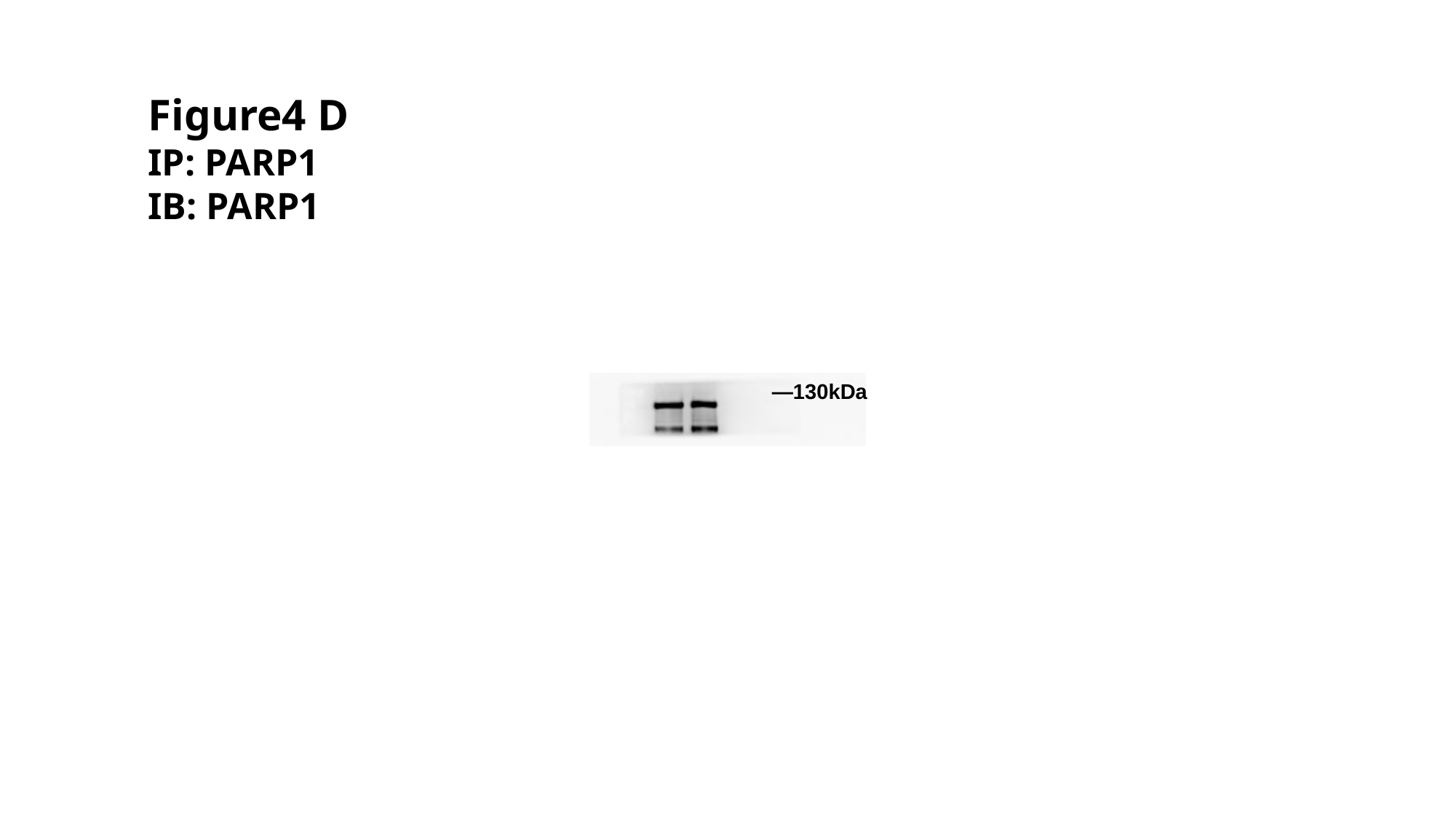

Figure4 D
IP: PARP1
IB: PARP1
—130kDa

## Slide 39
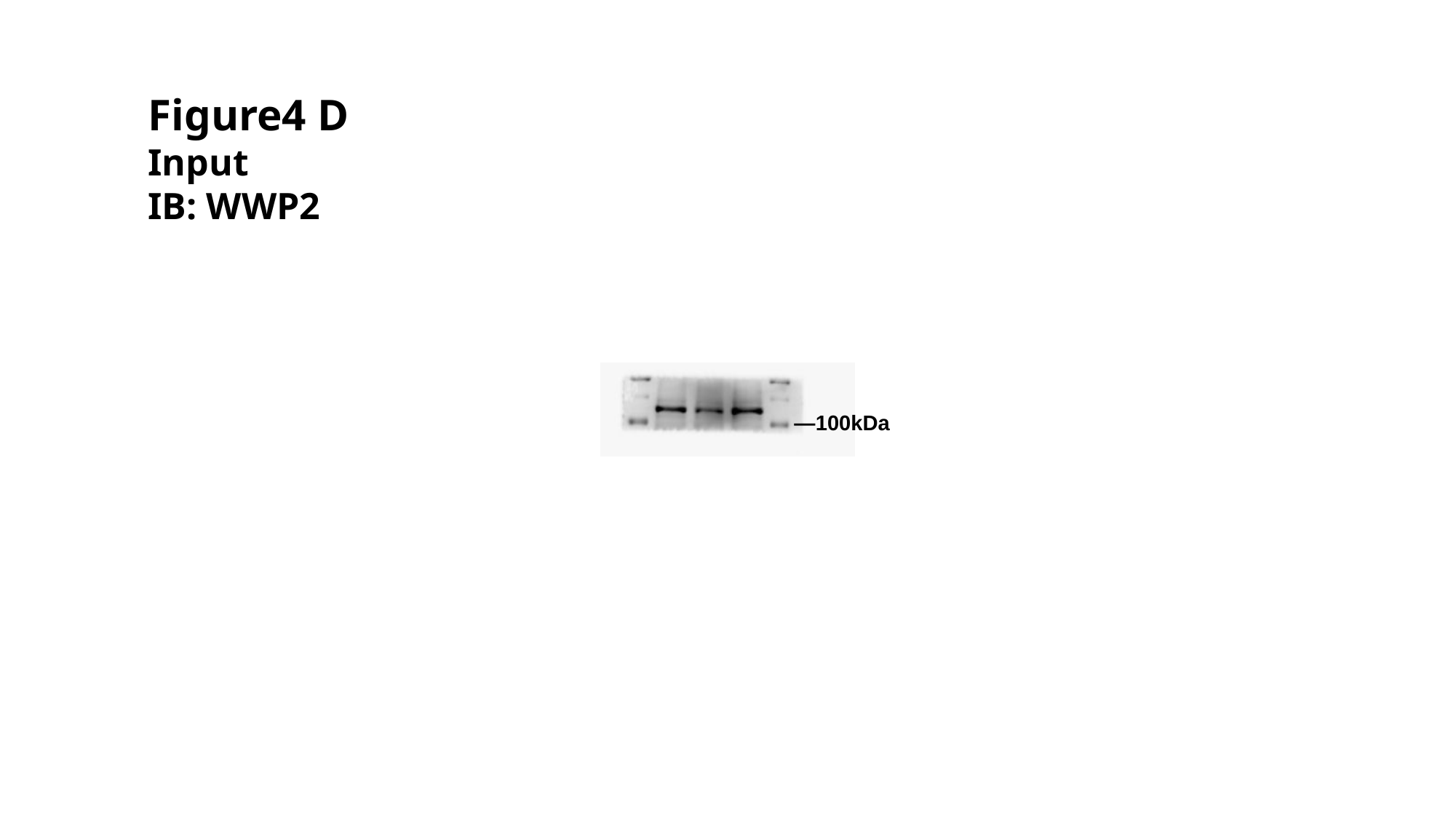

Figure4 D
Input
IB: WWP2
—100kDa

## Slide 40
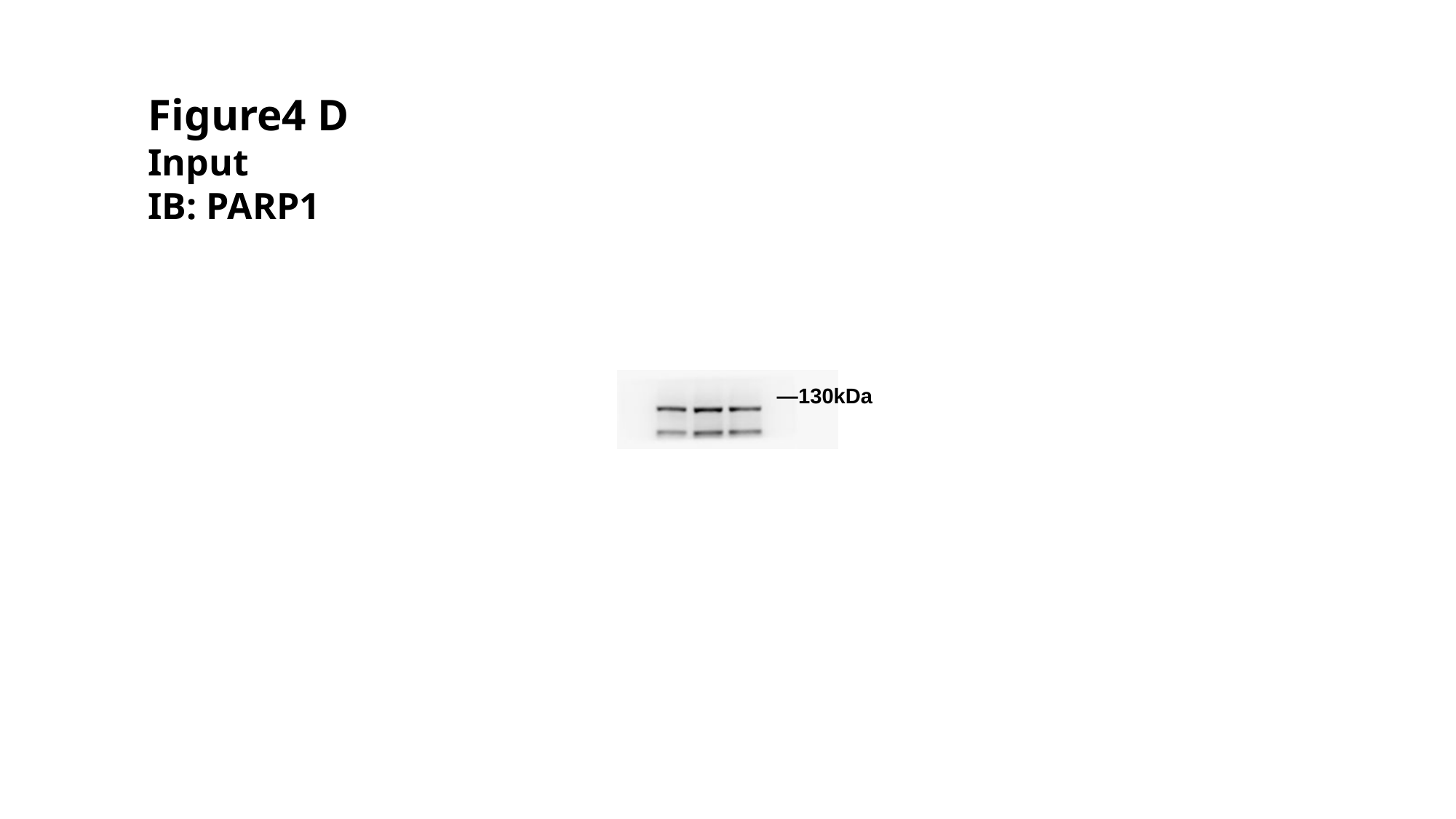

Figure4 D
Input
IB: PARP1
—130kDa

## Slide 41
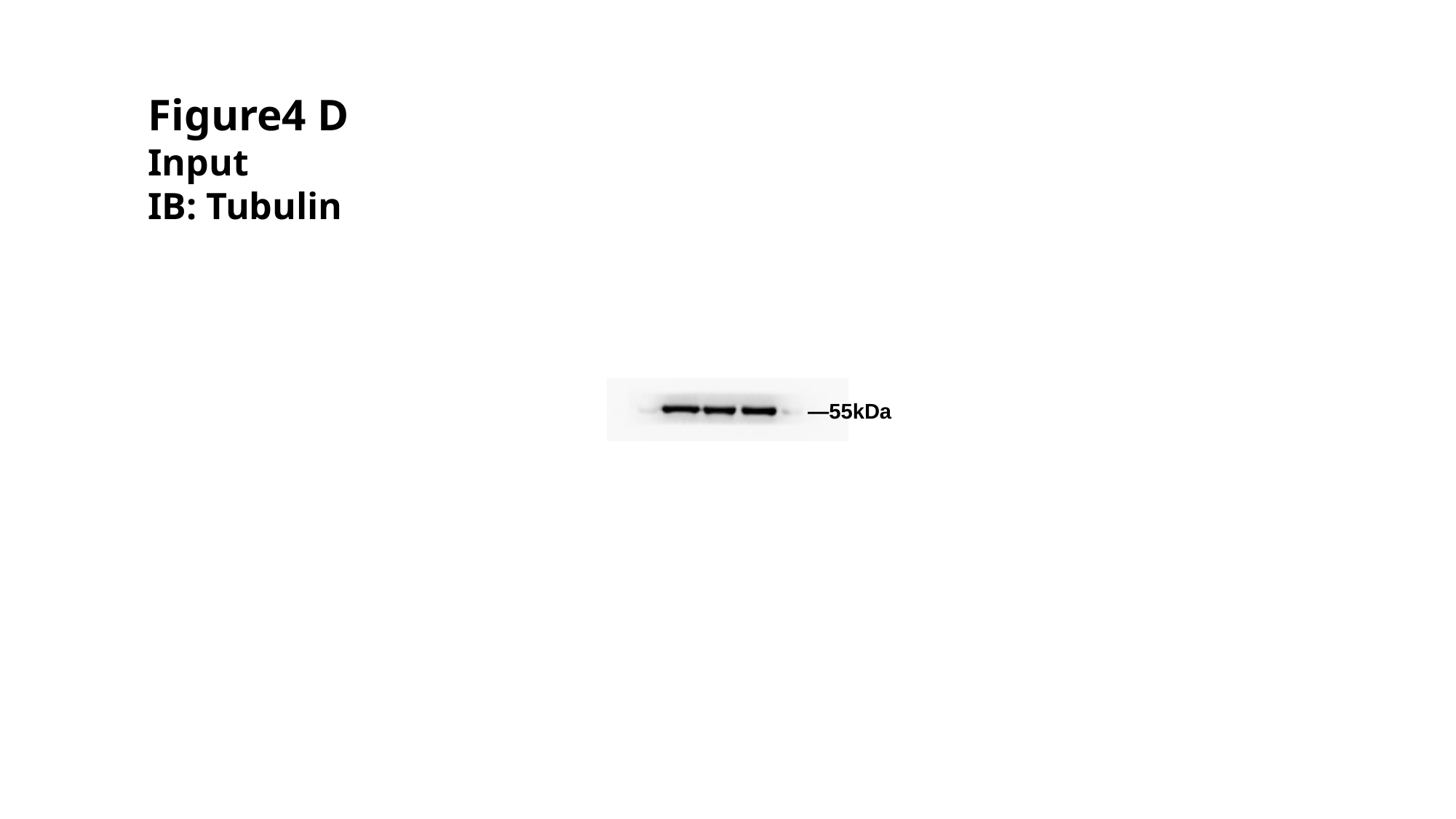

Figure4 D
Input
IB: Tubulin
—55kDa

## Slide 42
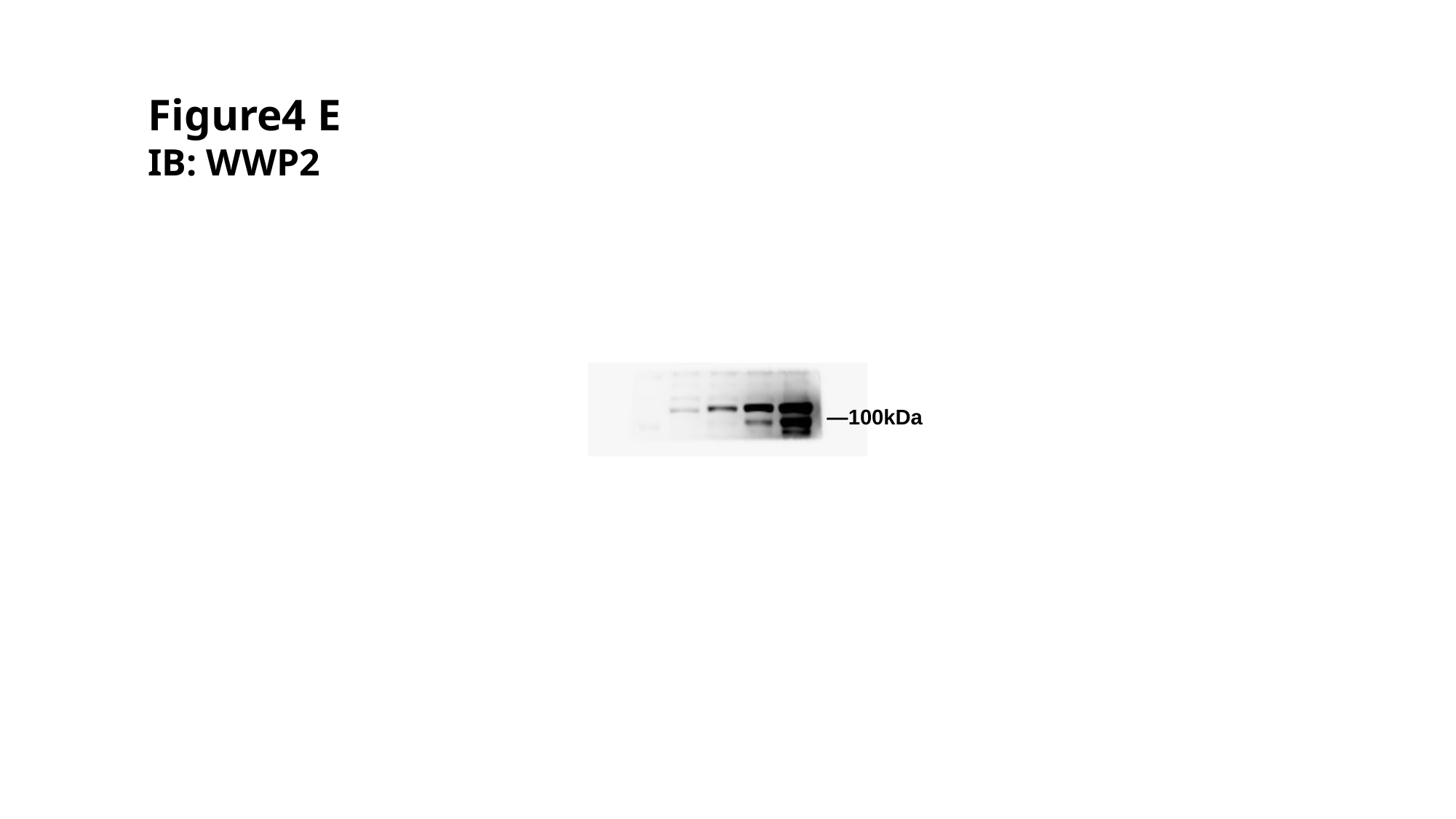

Figure4 E
IB: WWP2
—100kDa

## Slide 43
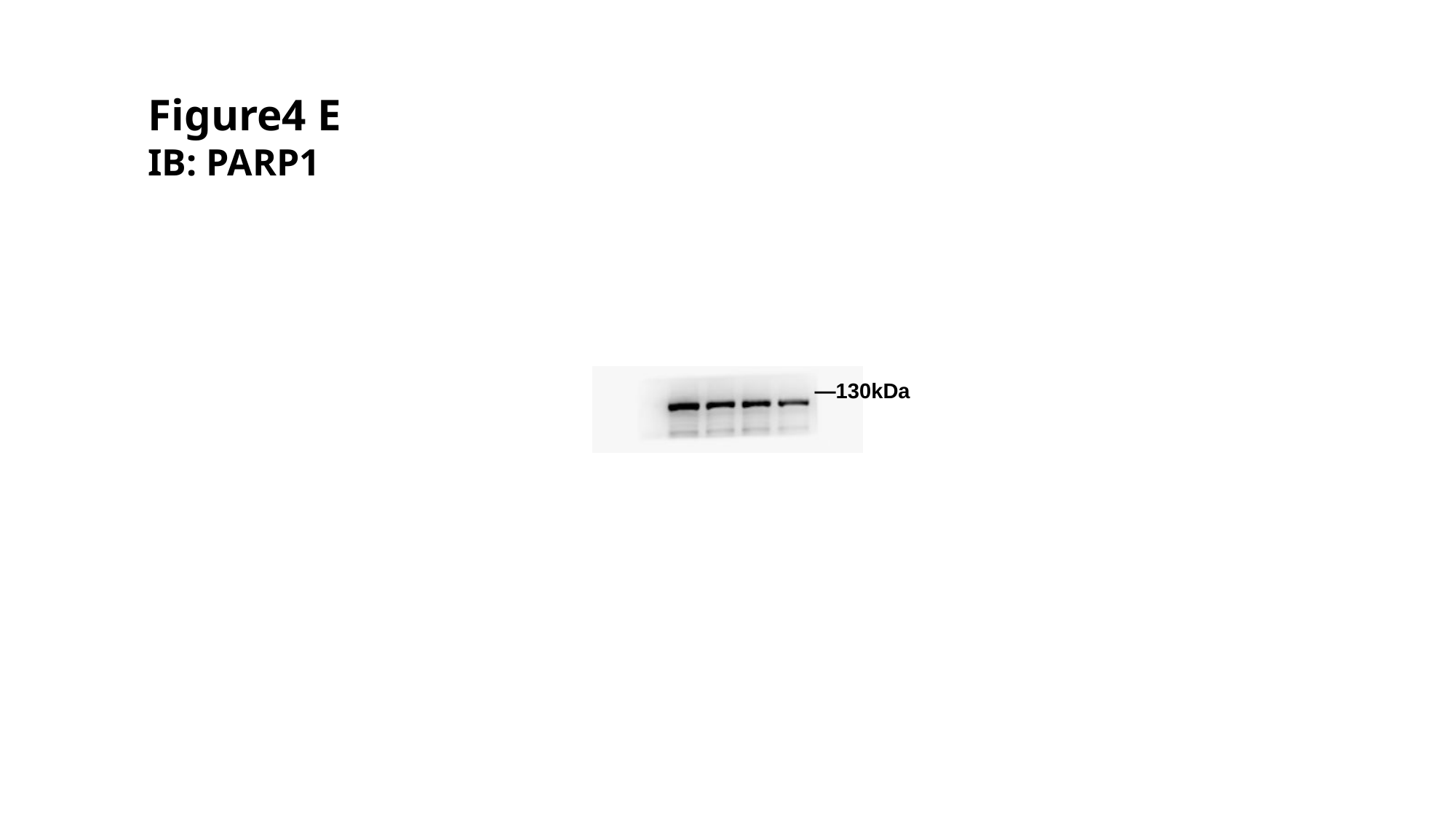

Figure4 E
IB: PARP1
—130kDa

## Slide 44
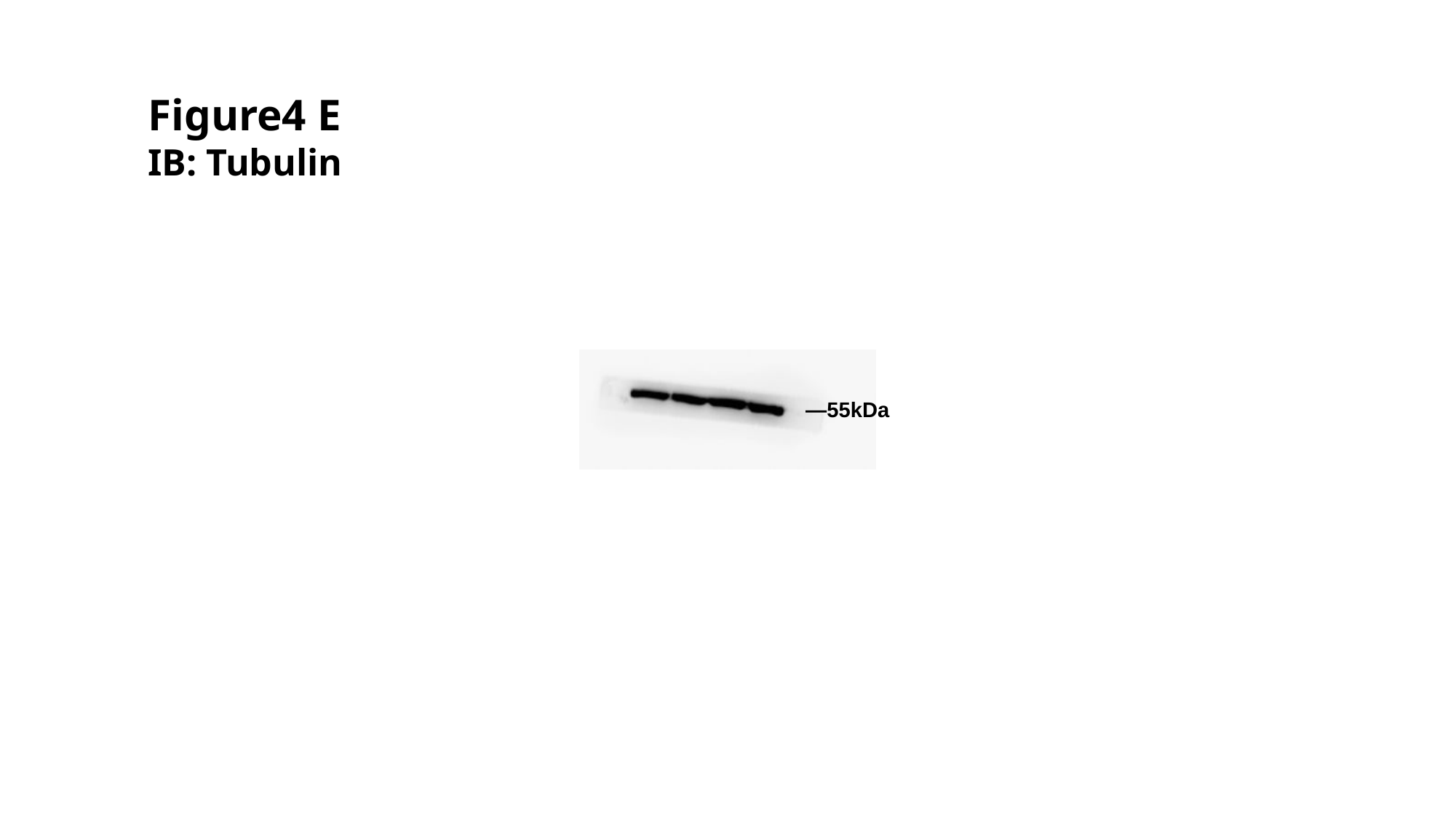

Figure4 E
IB: Tubulin
—55kDa

## Slide 45
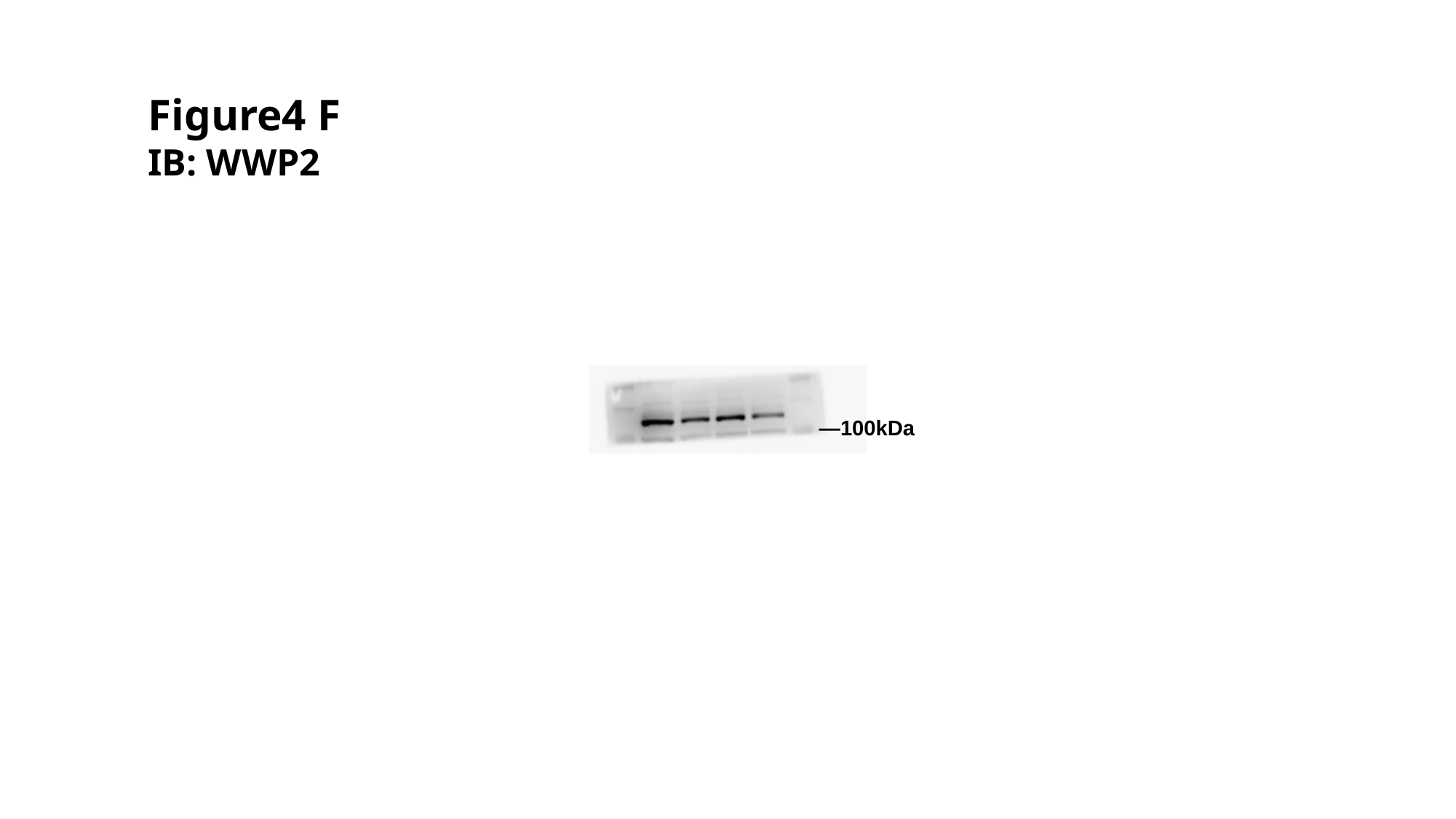

Figure4 F
IB: WWP2
—100kDa

## Slide 46
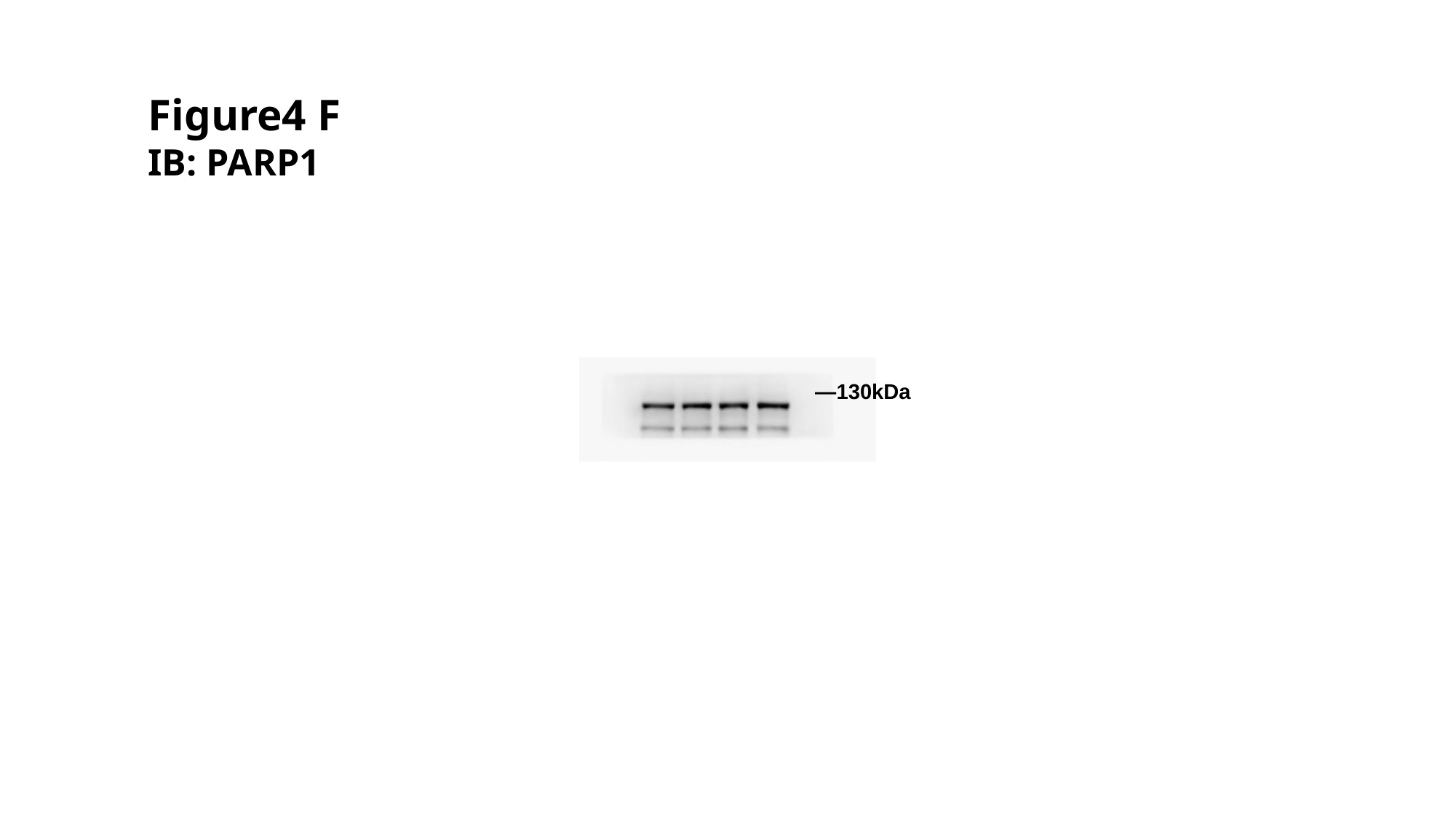

Figure4 F
IB: PARP1
—130kDa

## Slide 47
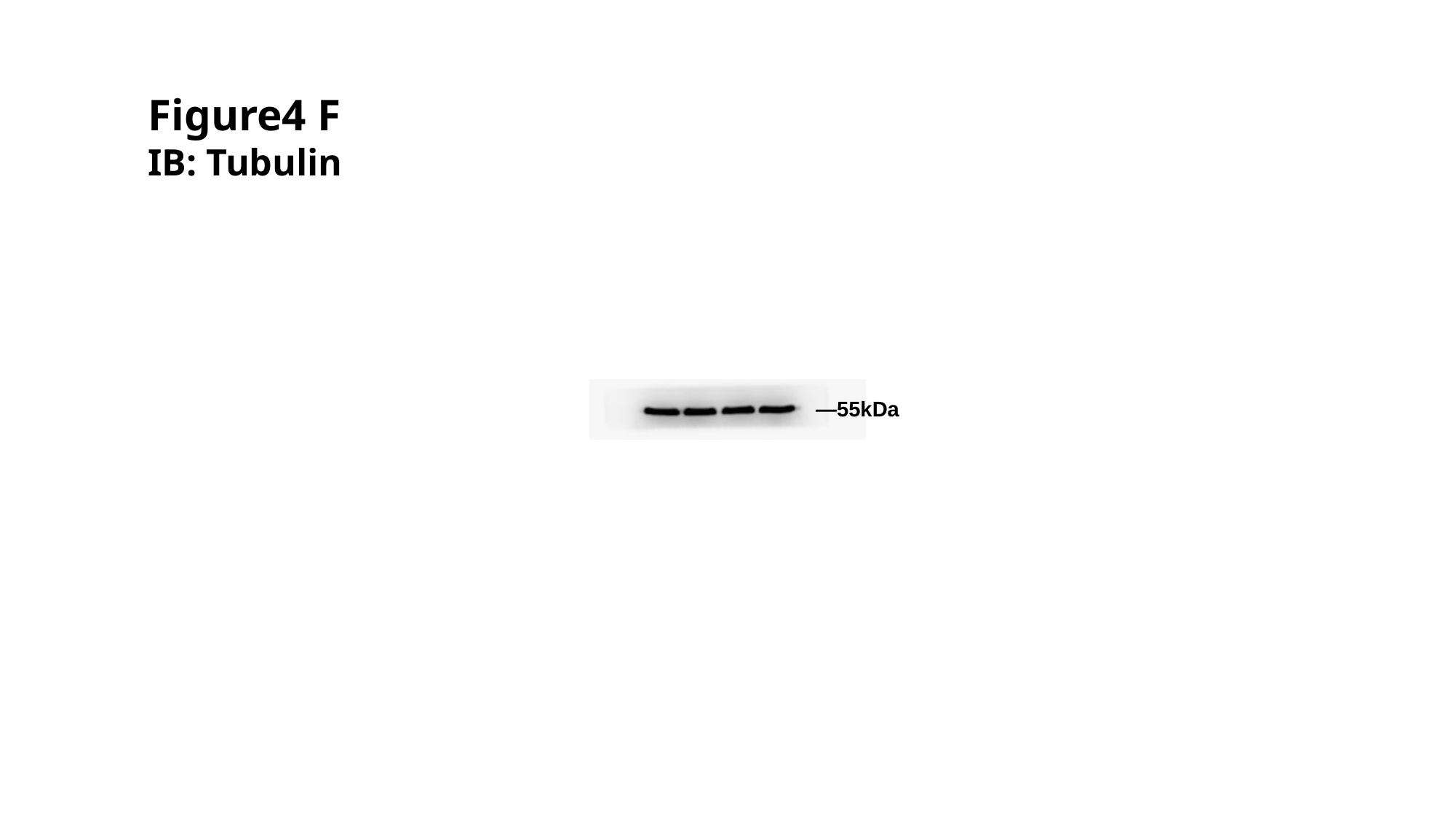

Figure4 F
IB: Tubulin
—55kDa

## Slide 48
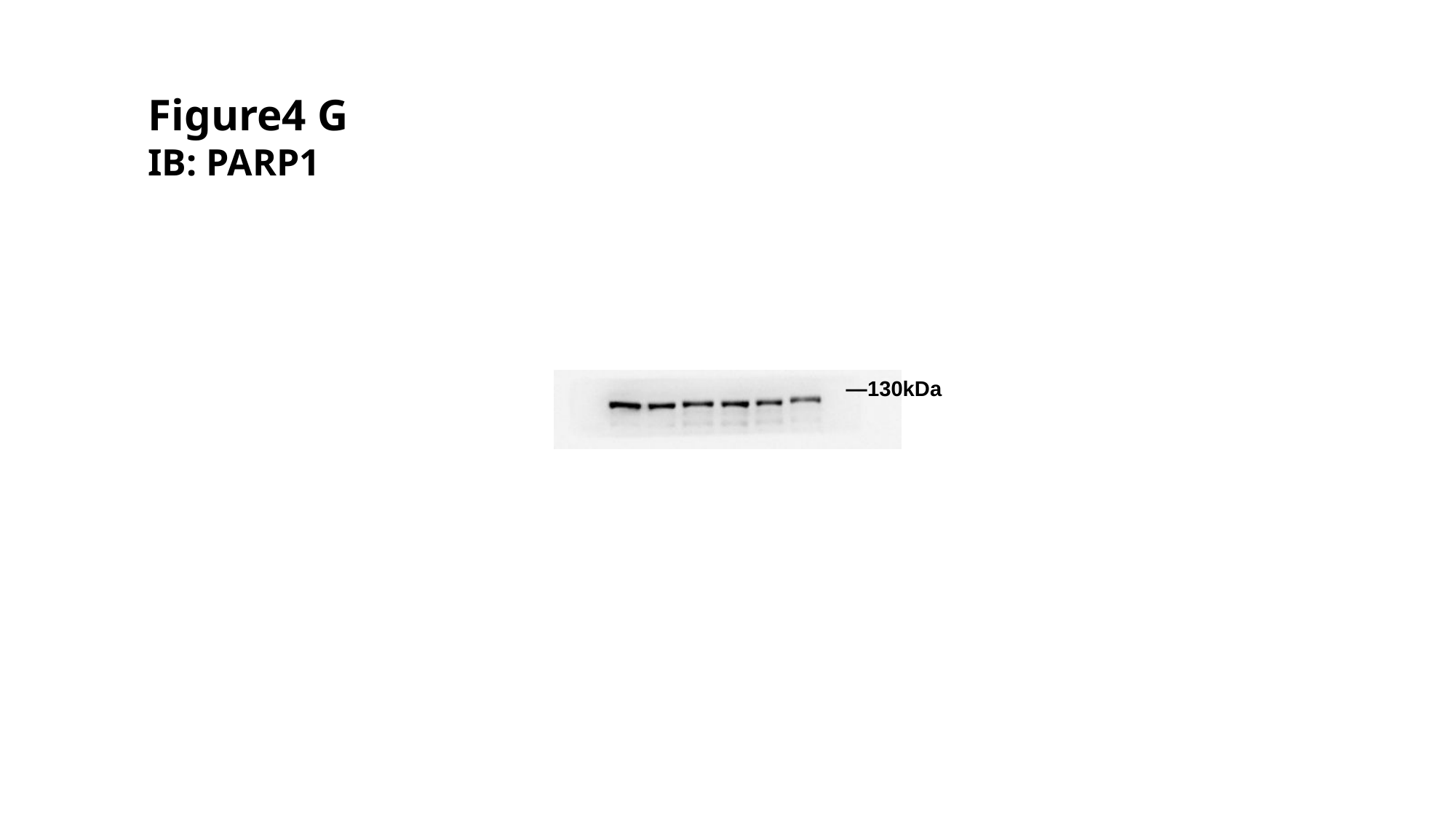

Figure4 G
IB: PARP1
—130kDa

## Slide 49
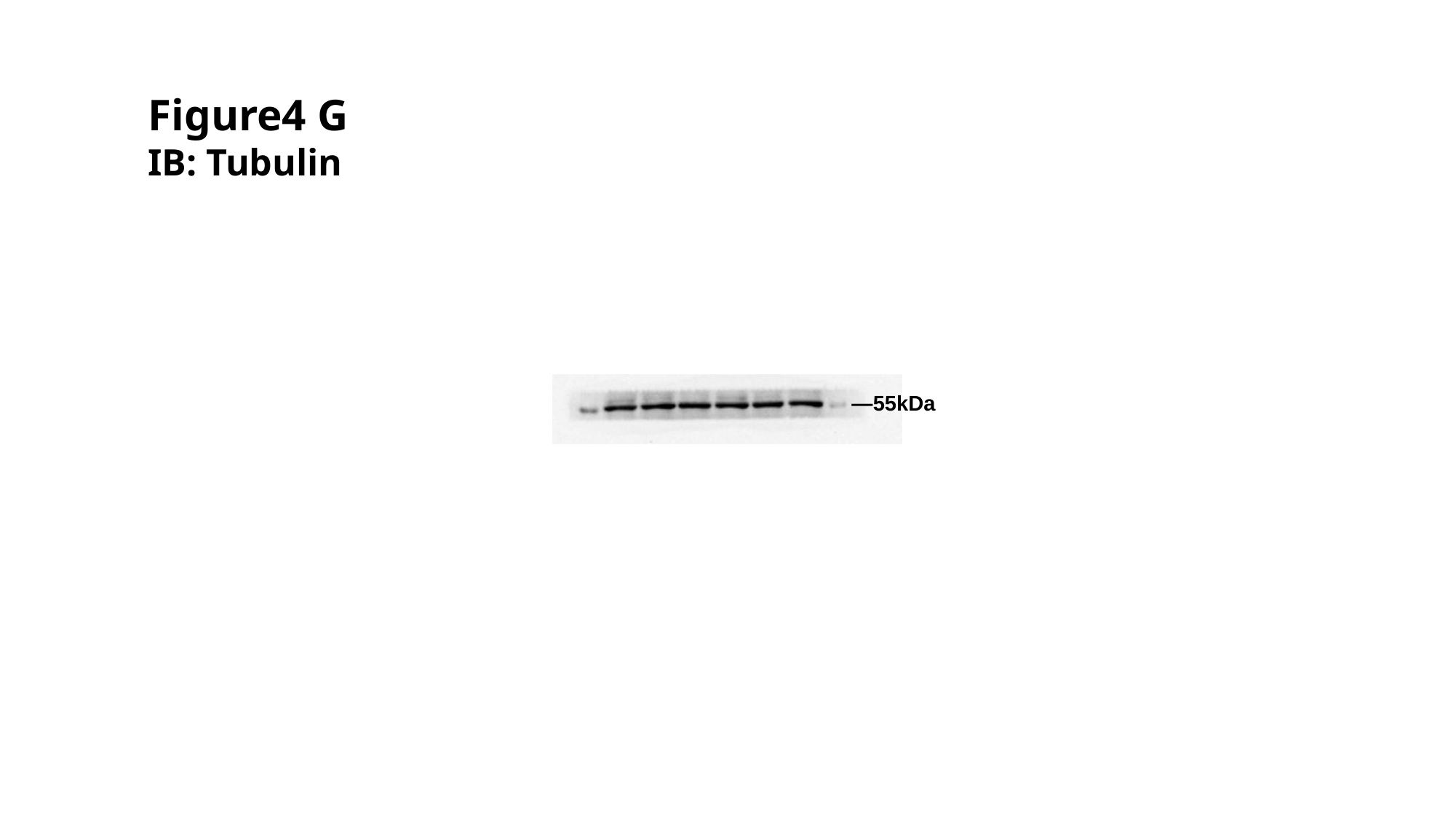

Figure4 G
IB: Tubulin
—55kDa

## Slide 50
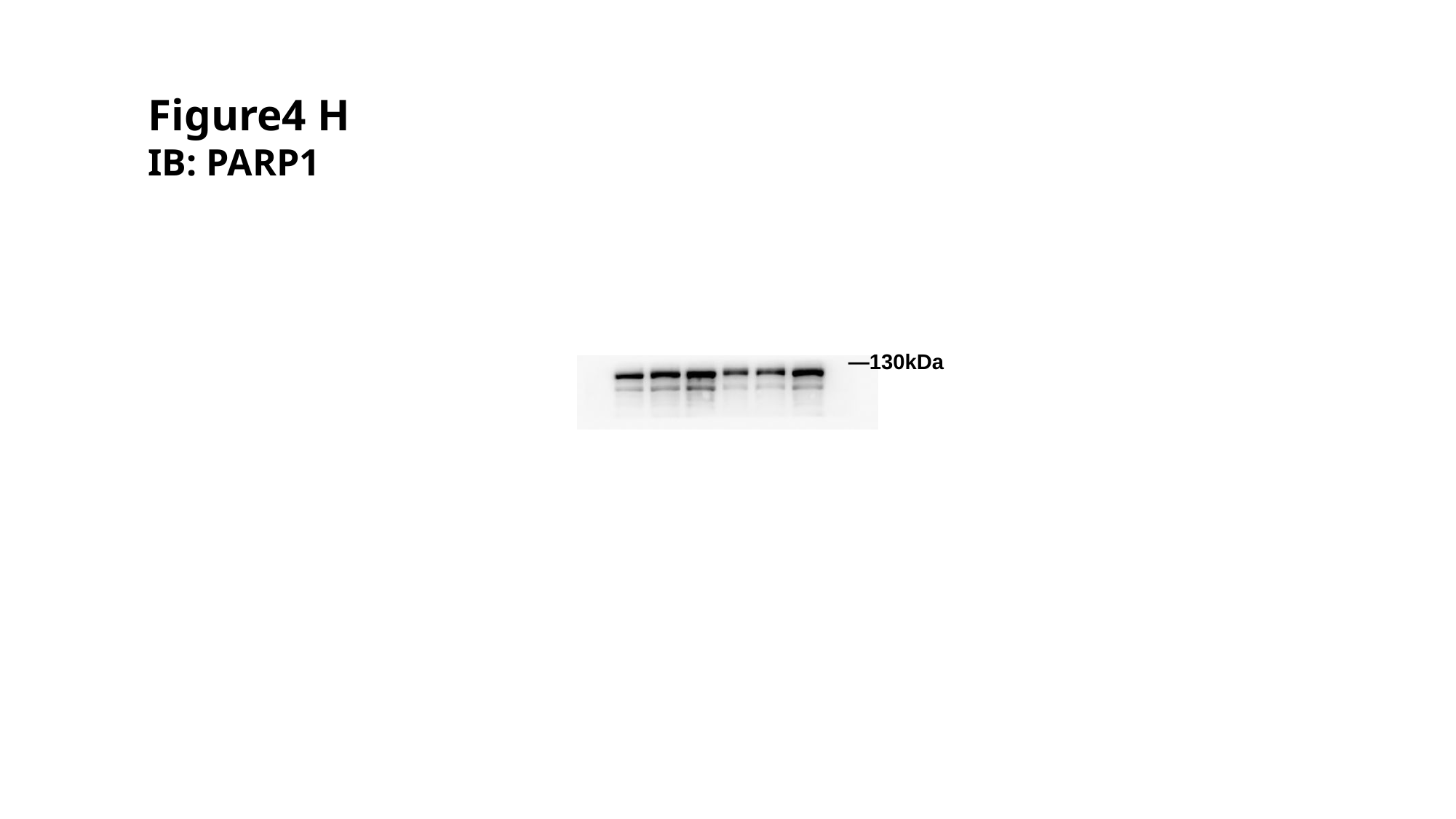

Figure4 H
IB: PARP1
—130kDa

## Slide 51
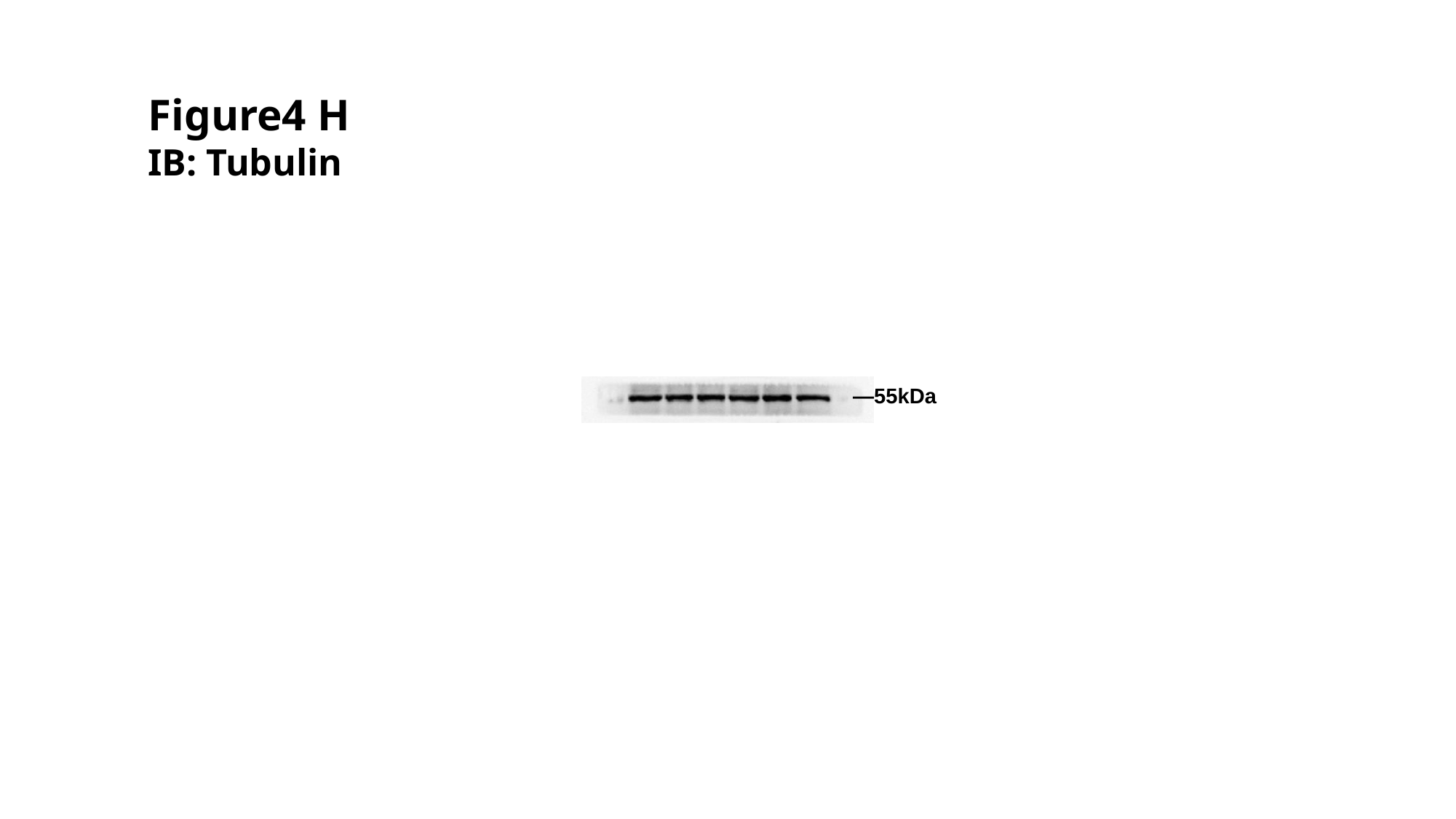

Figure4 H
IB: Tubulin
—55kDa

## Slide 52
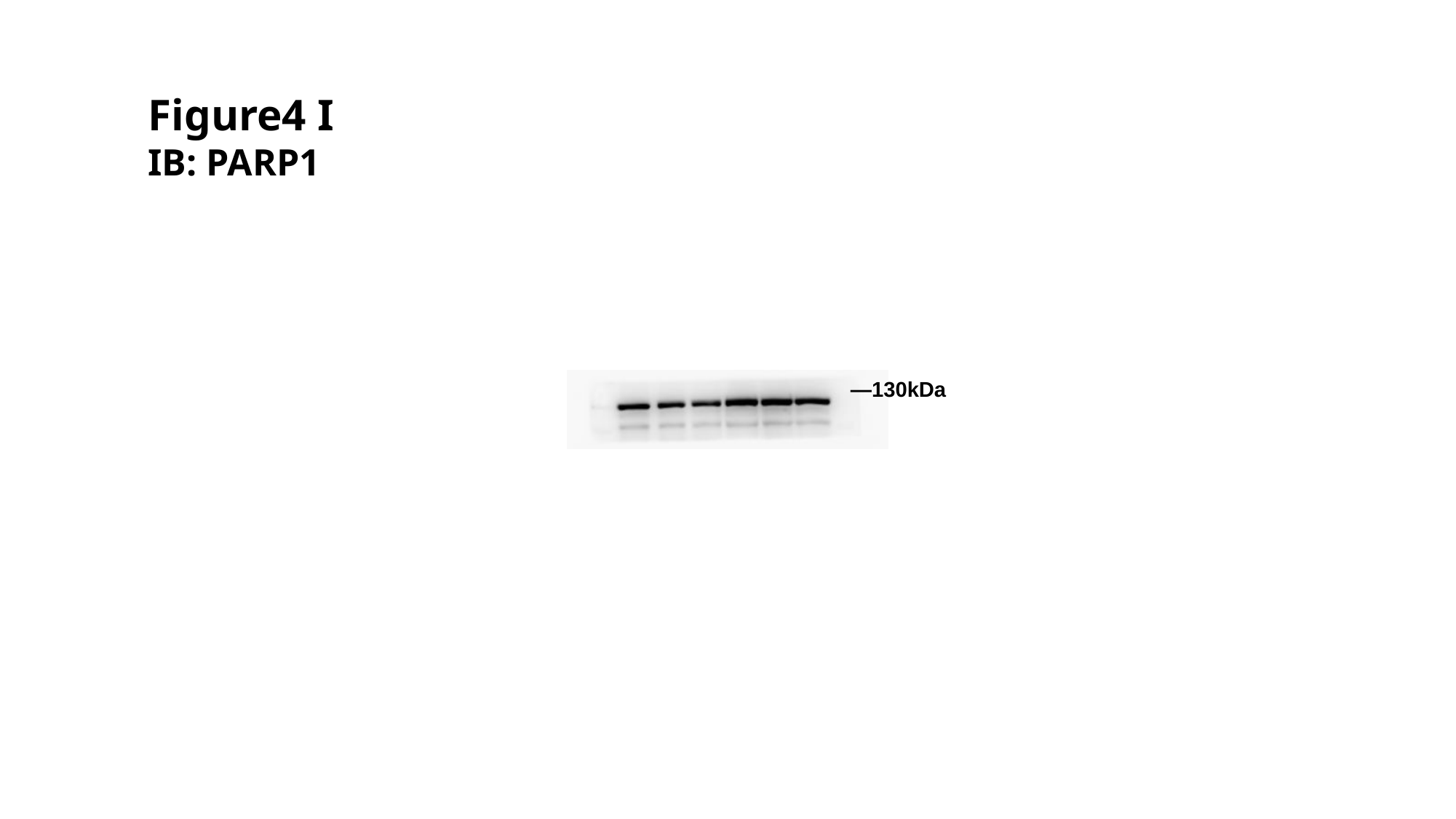

Figure4 I
IB: PARP1
—130kDa

## Slide 53
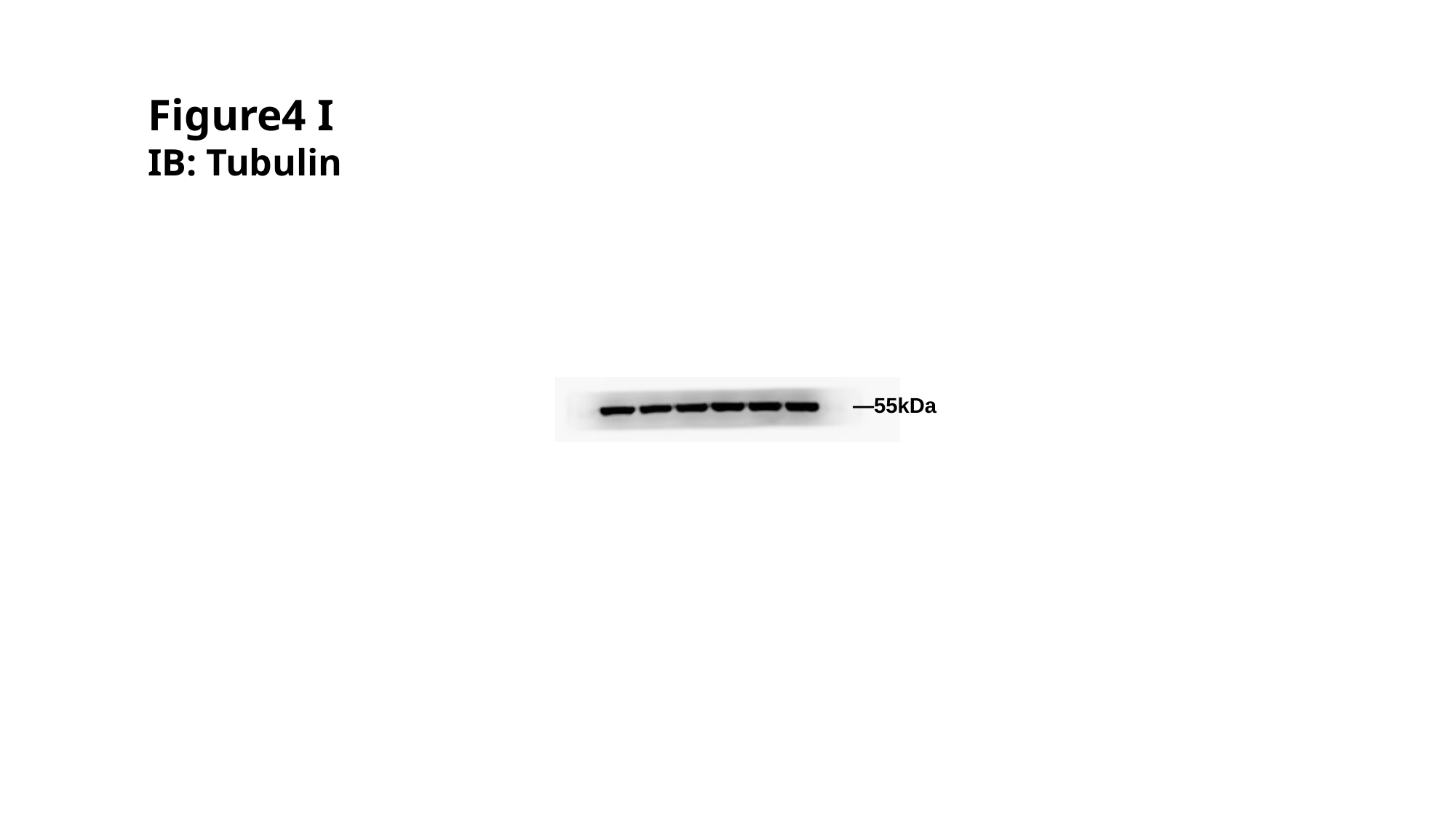

Figure4 I
IB: Tubulin
—55kDa

## Slide 54
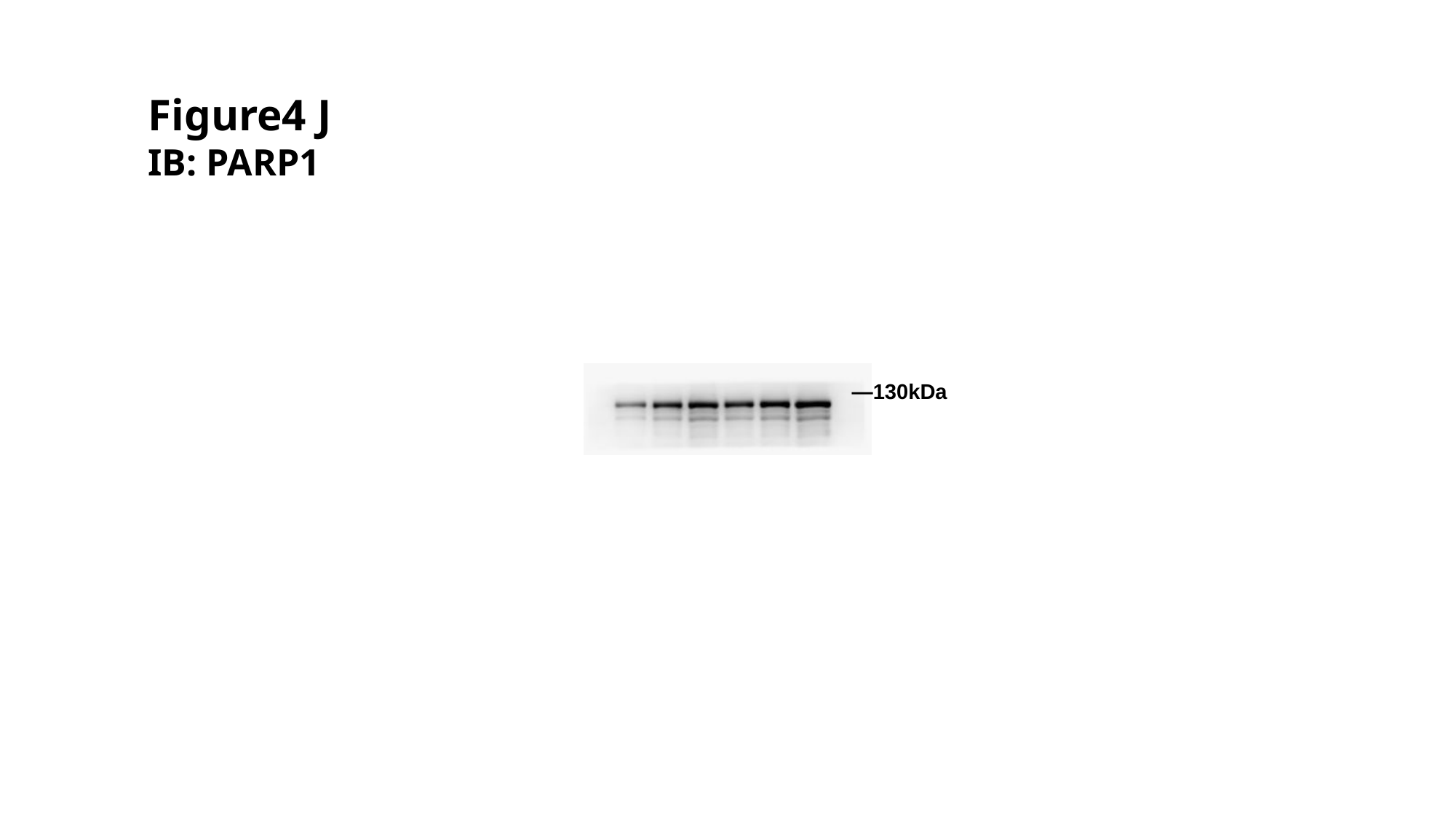

Figure4 J
IB: PARP1
—130kDa

## Slide 55
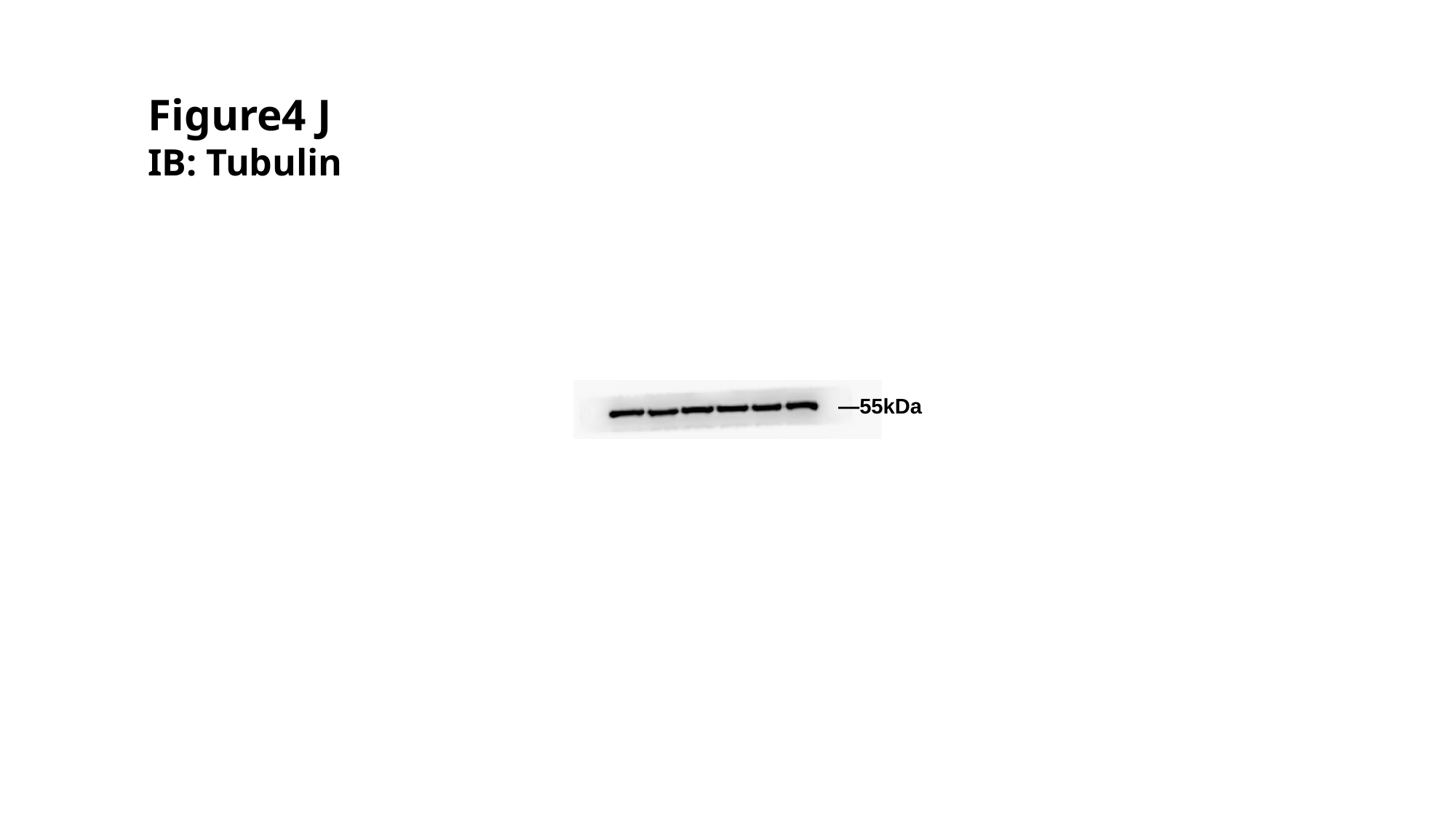

Figure4 J
IB: Tubulin
—55kDa

## Slide 56
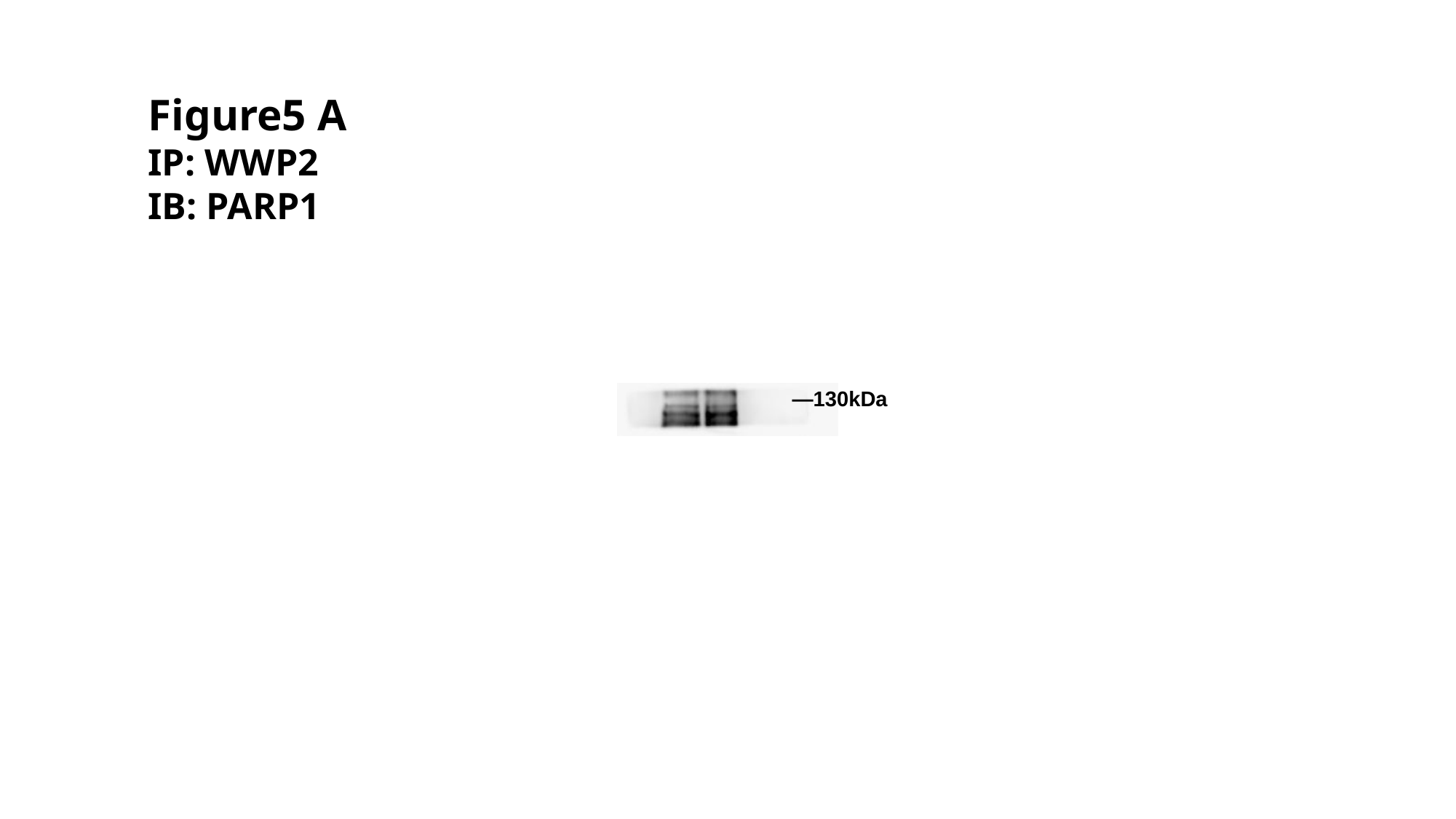

Figure5 A
IP: WWP2
IB: PARP1
—130kDa

## Slide 57
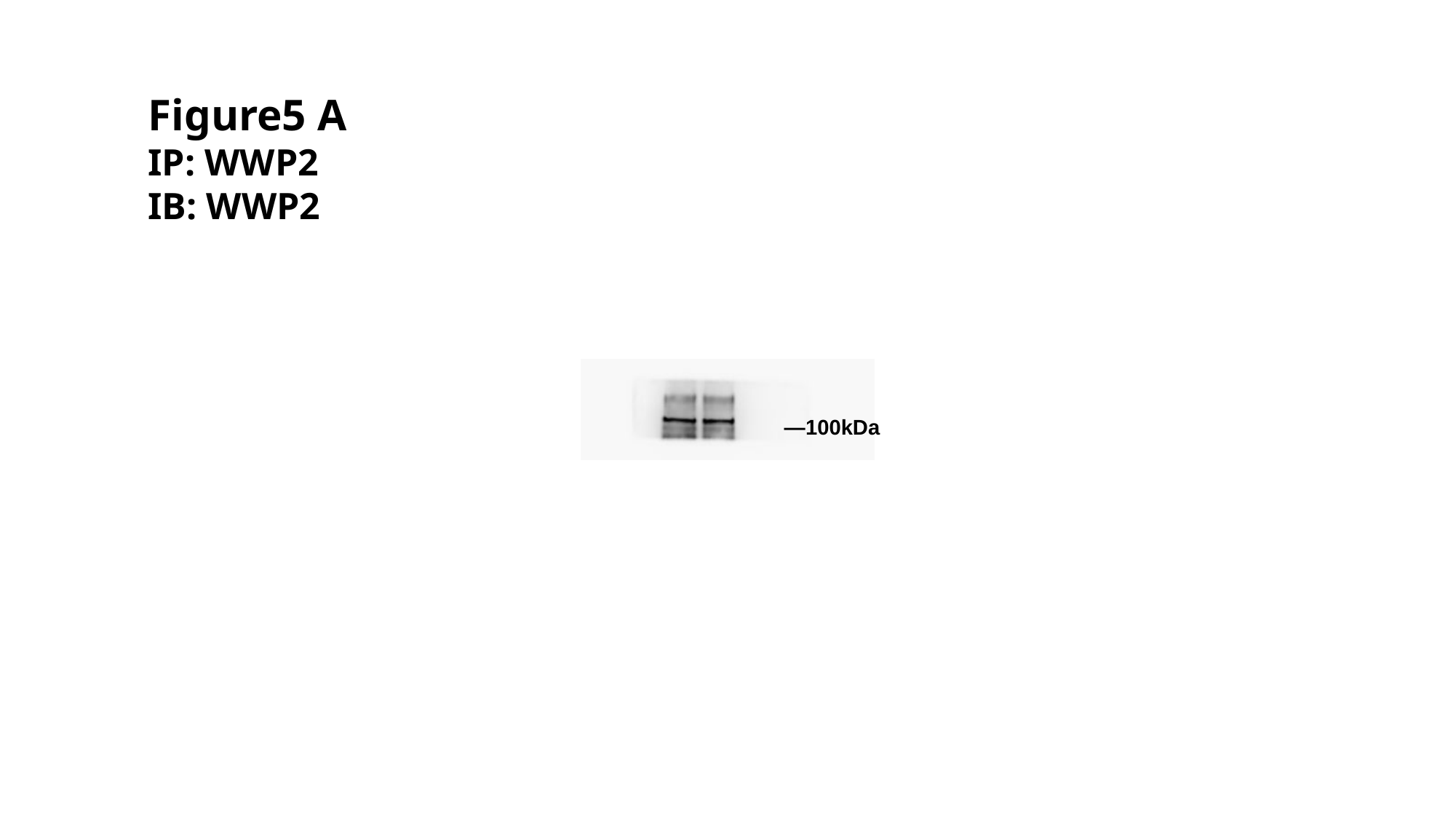

Figure5 A
IP: WWP2
IB: WWP2
—100kDa

## Slide 58
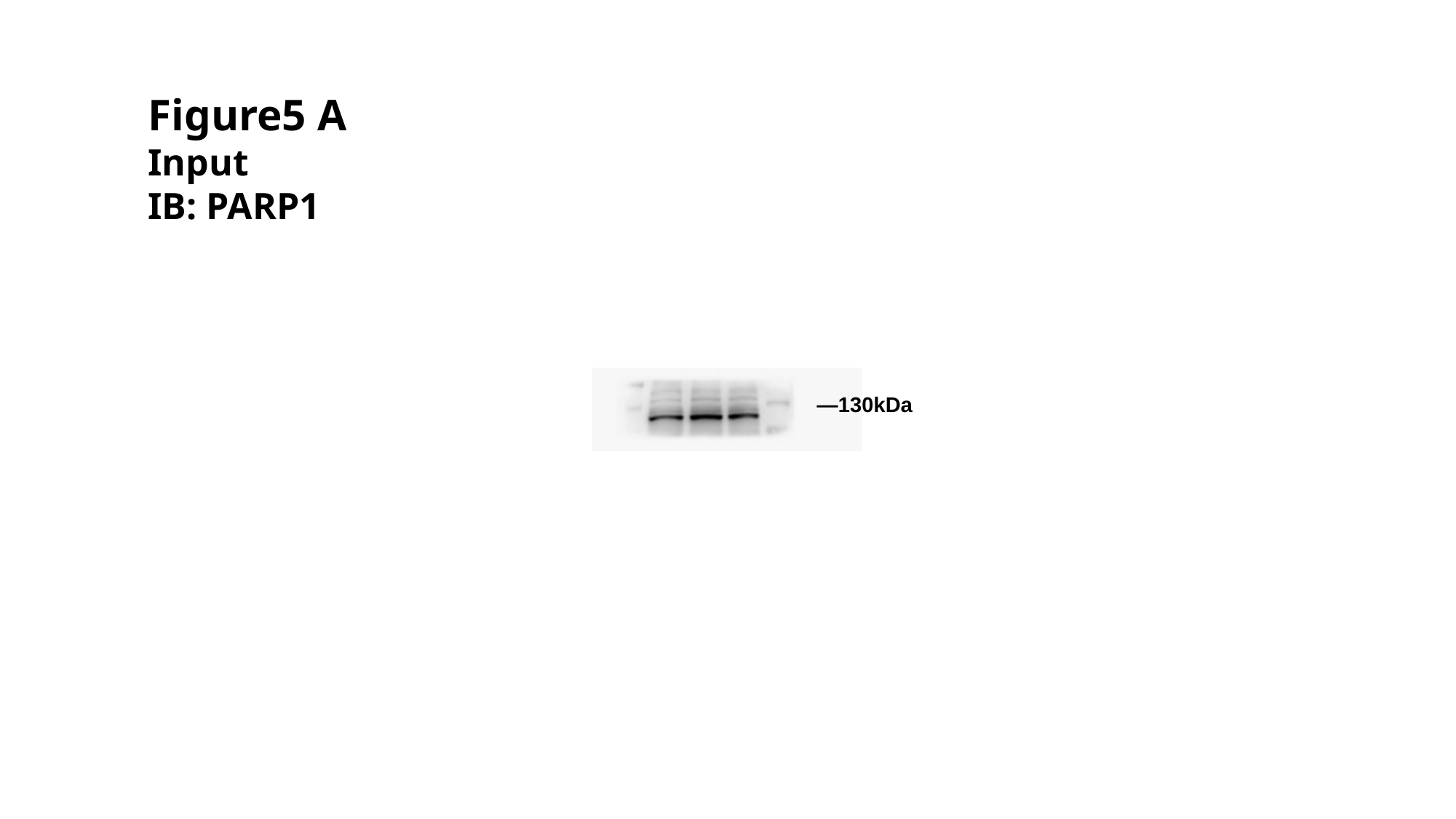

Figure5 A
Input
IB: PARP1
—130kDa

## Slide 59
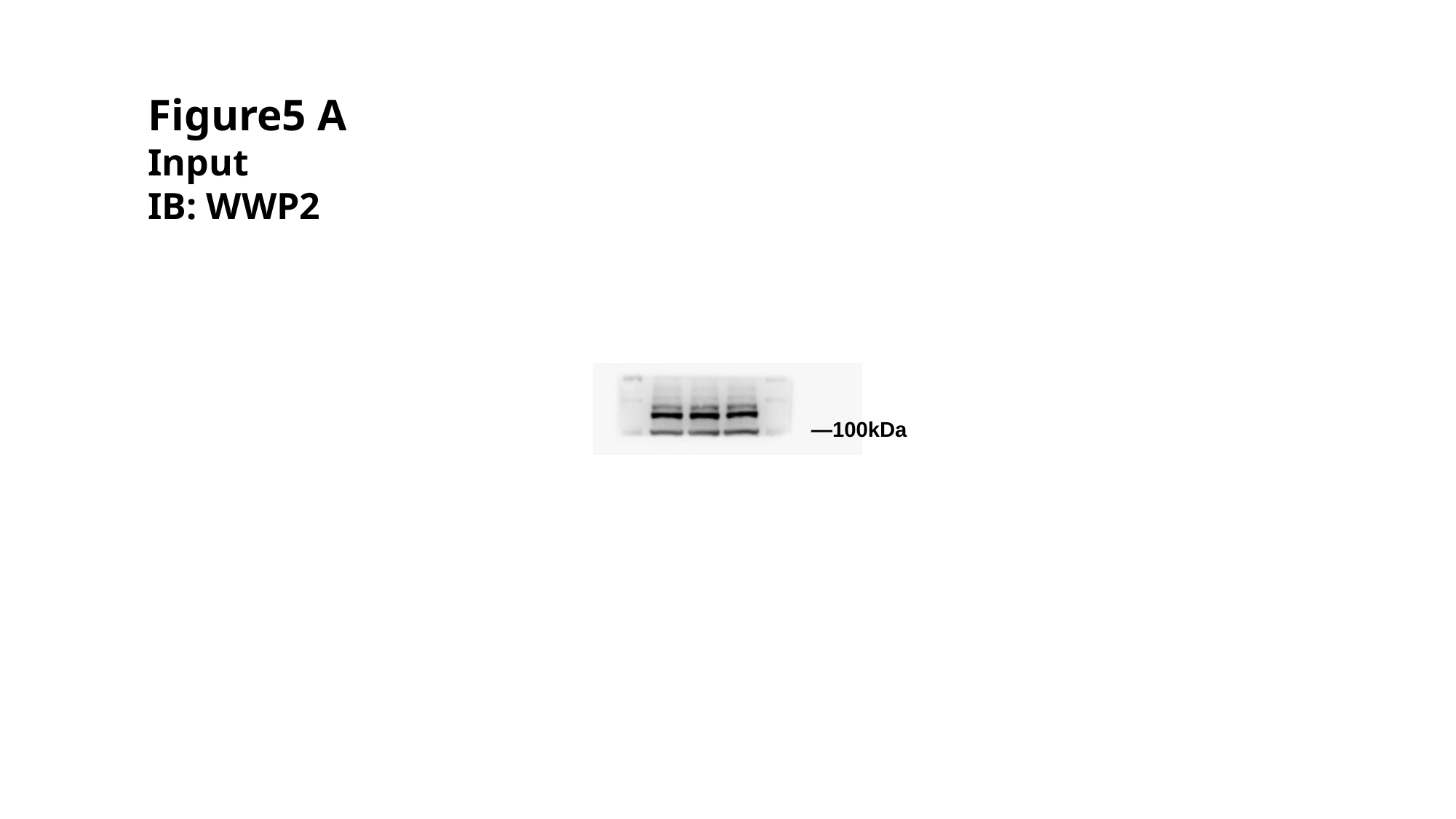

Figure5 A
Input
IB: WWP2
—100kDa

## Slide 60
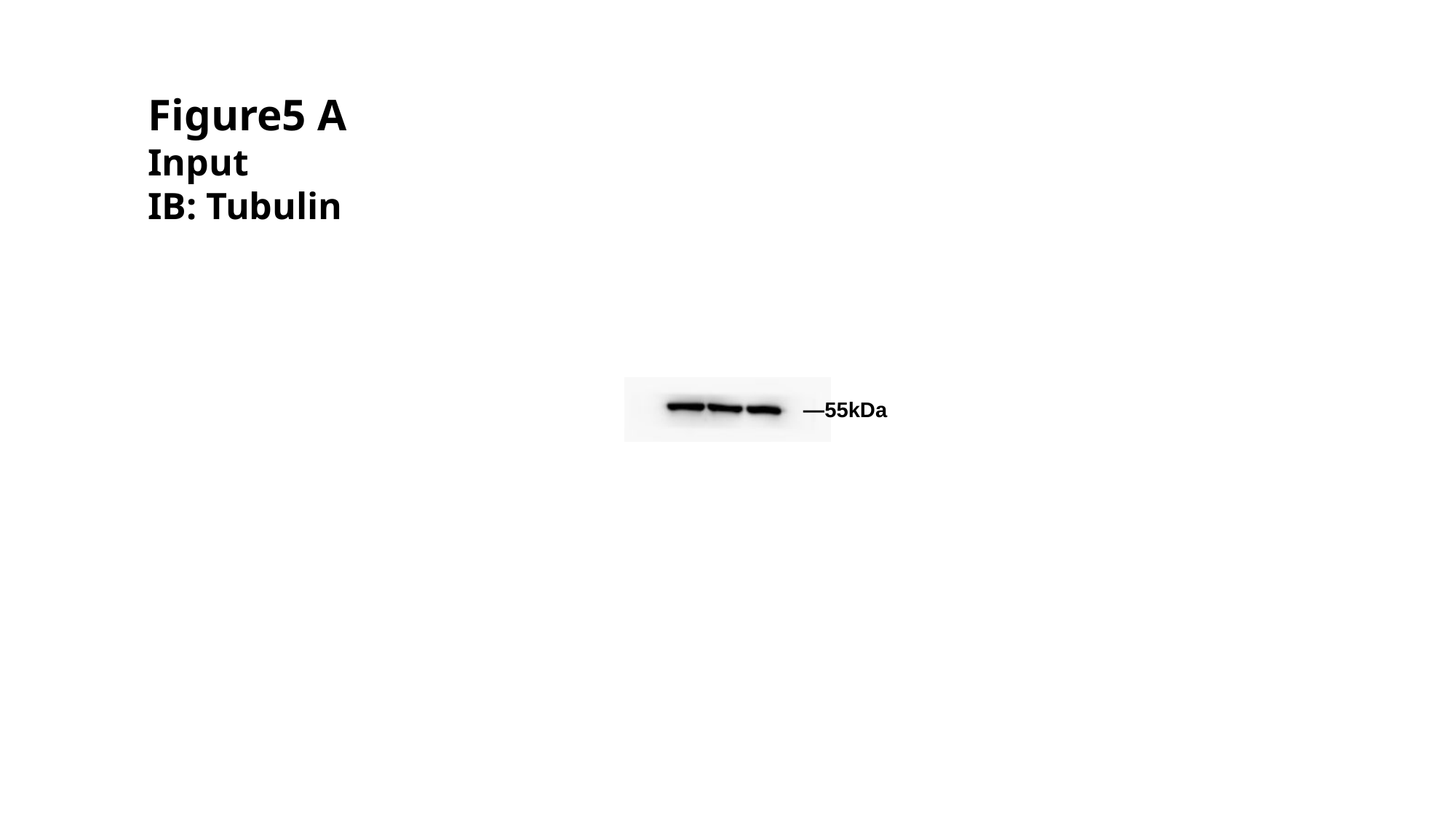

Figure5 A
Input
IB: Tubulin
—55kDa

## Slide 61
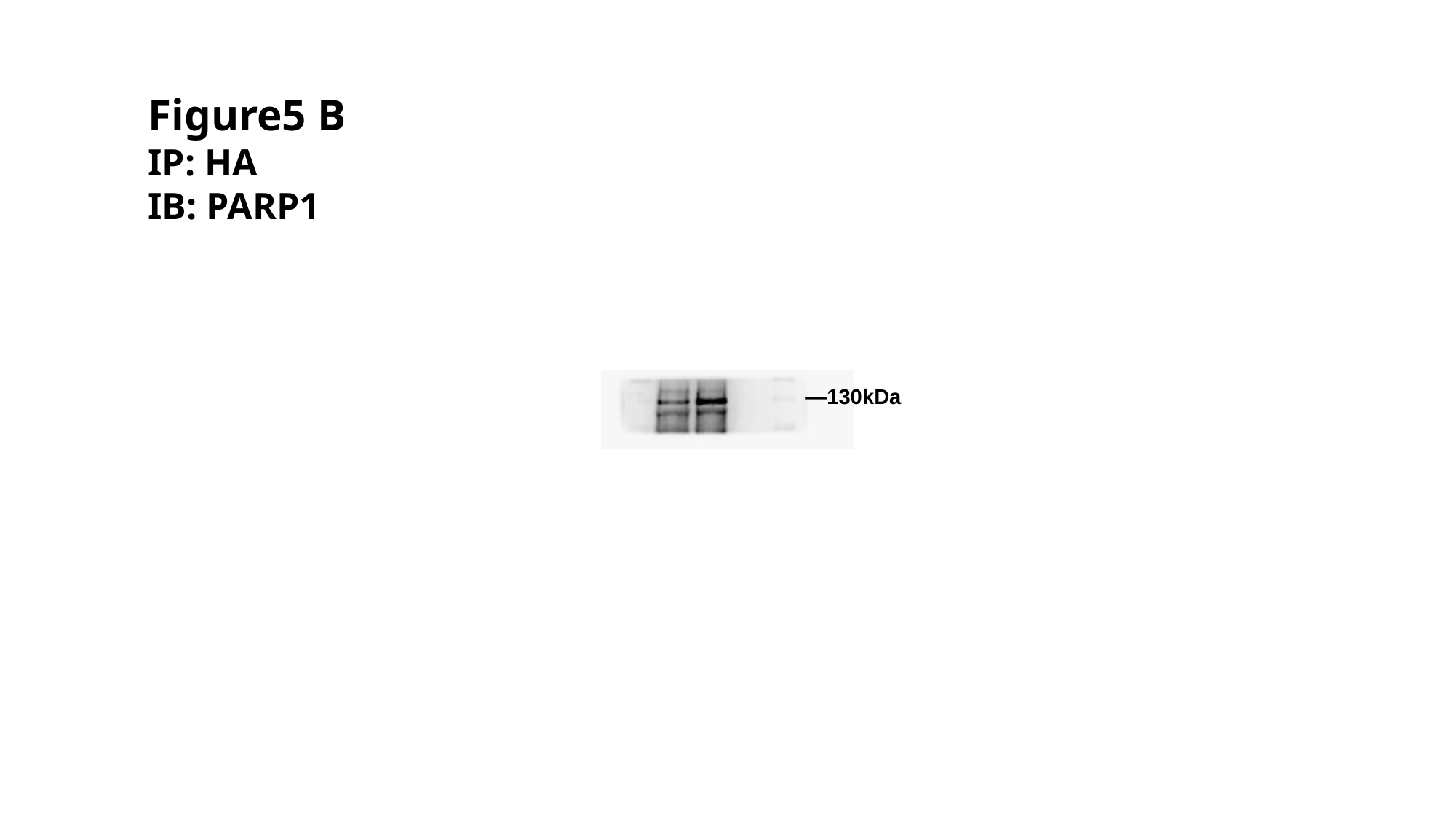

Figure5 B
IP: HA
IB: PARP1
—130kDa

## Slide 62
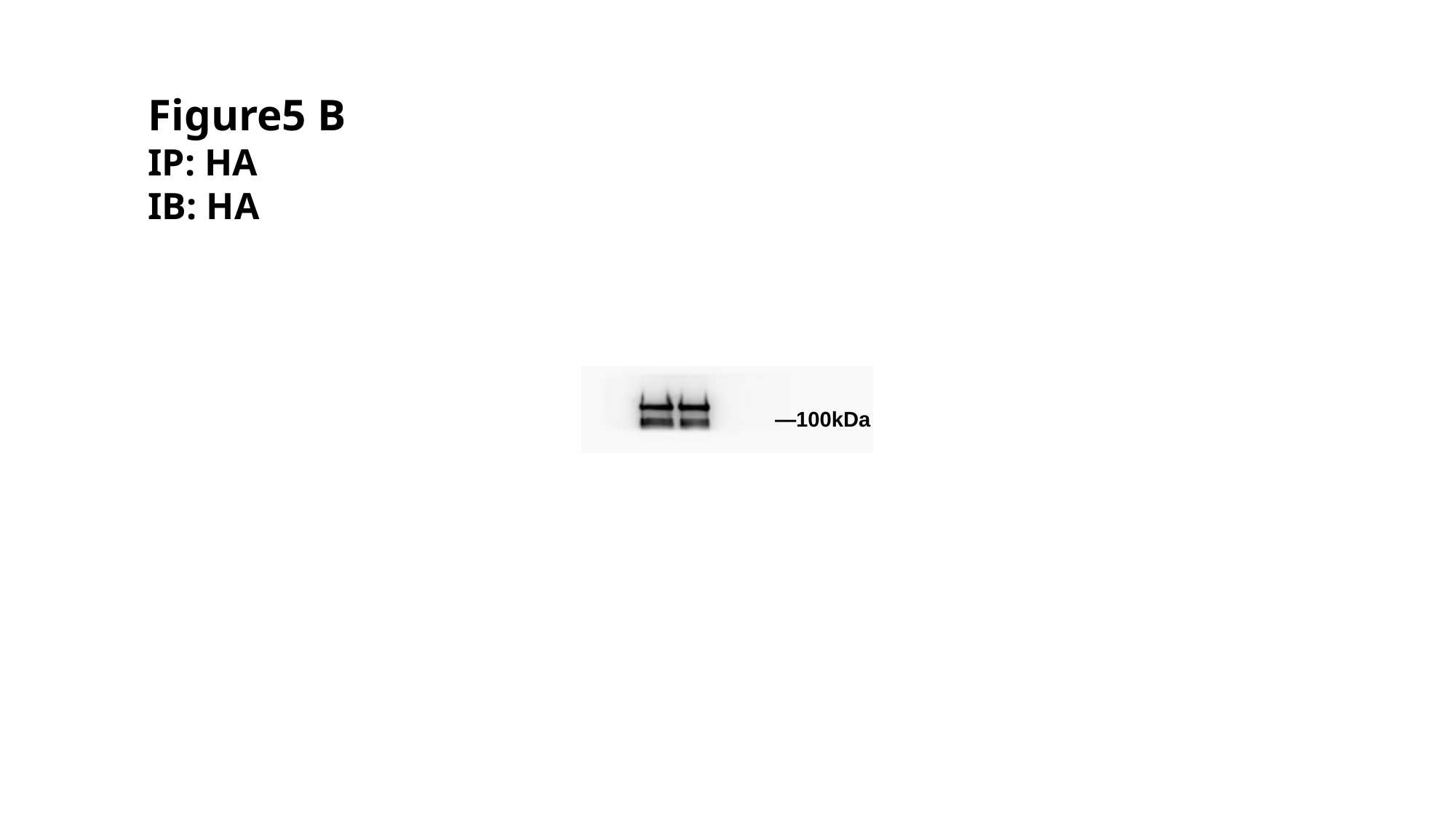

Figure5 B
IP: HA
IB: HA
—100kDa

## Slide 63
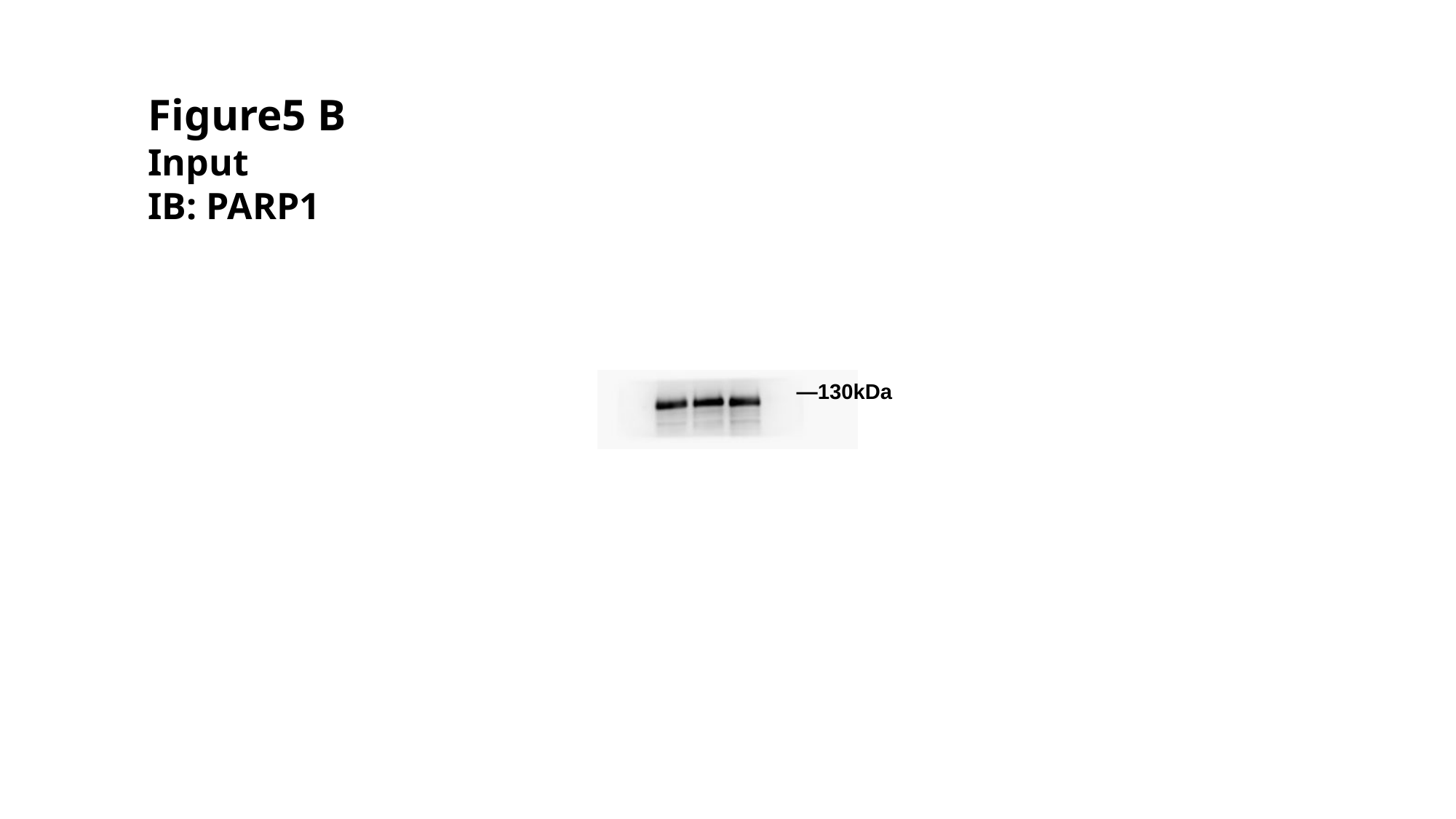

Figure5 B
Input
IB: PARP1
—130kDa

## Slide 64
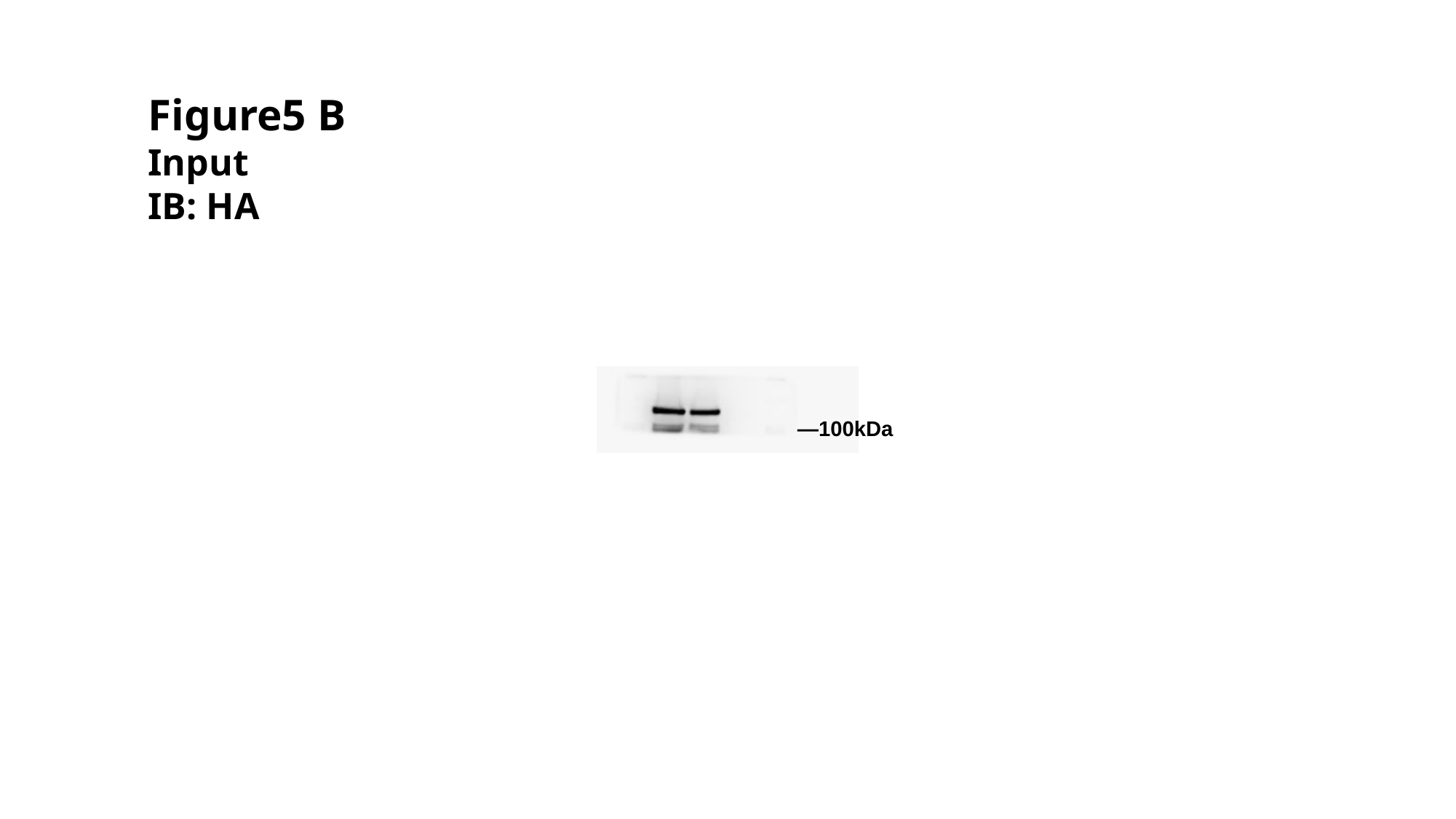

Figure5 B
Input
IB: HA
—100kDa

## Slide 65
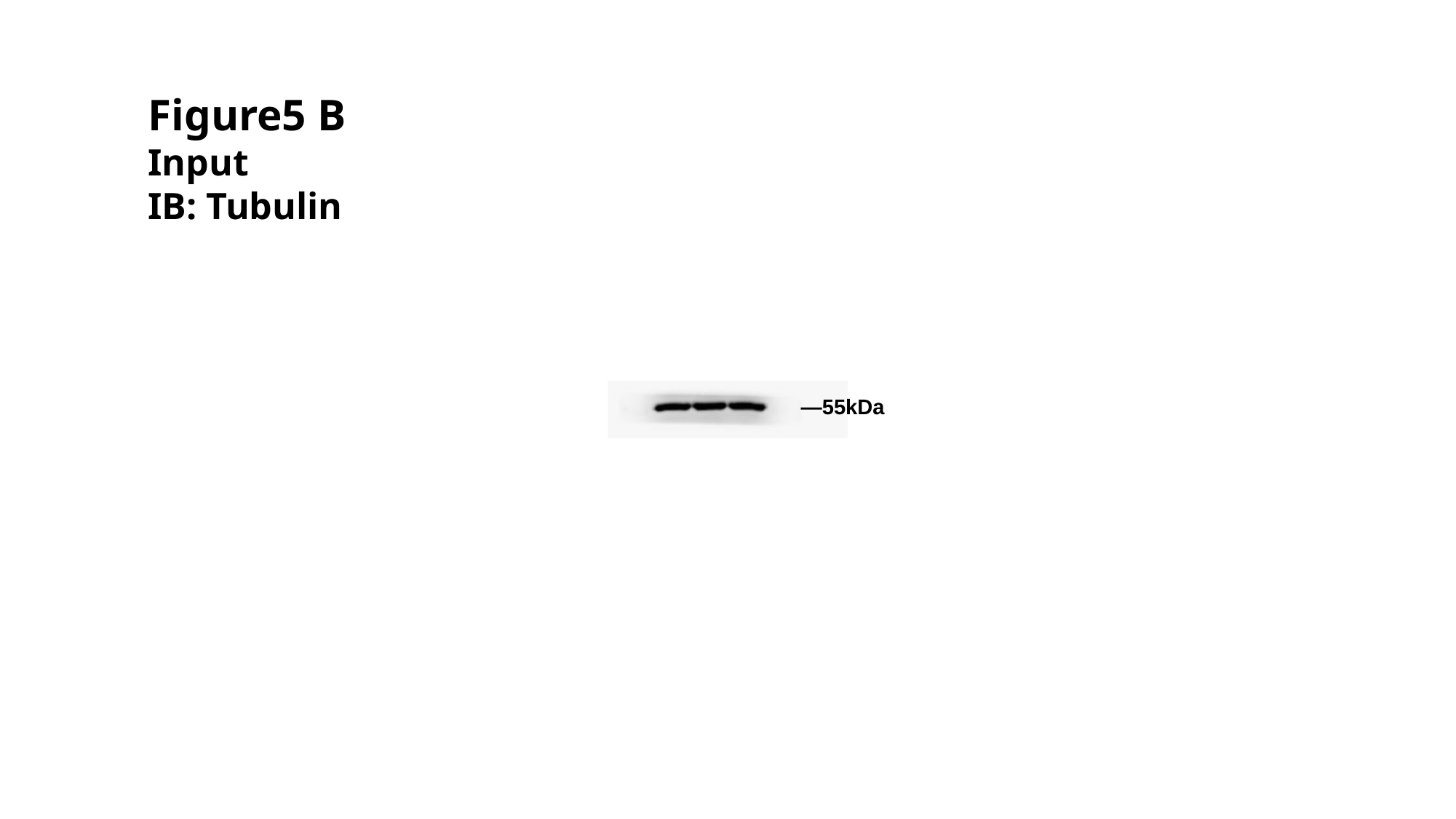

Figure5 B
Input
IB: Tubulin
—55kDa

## Slide 66
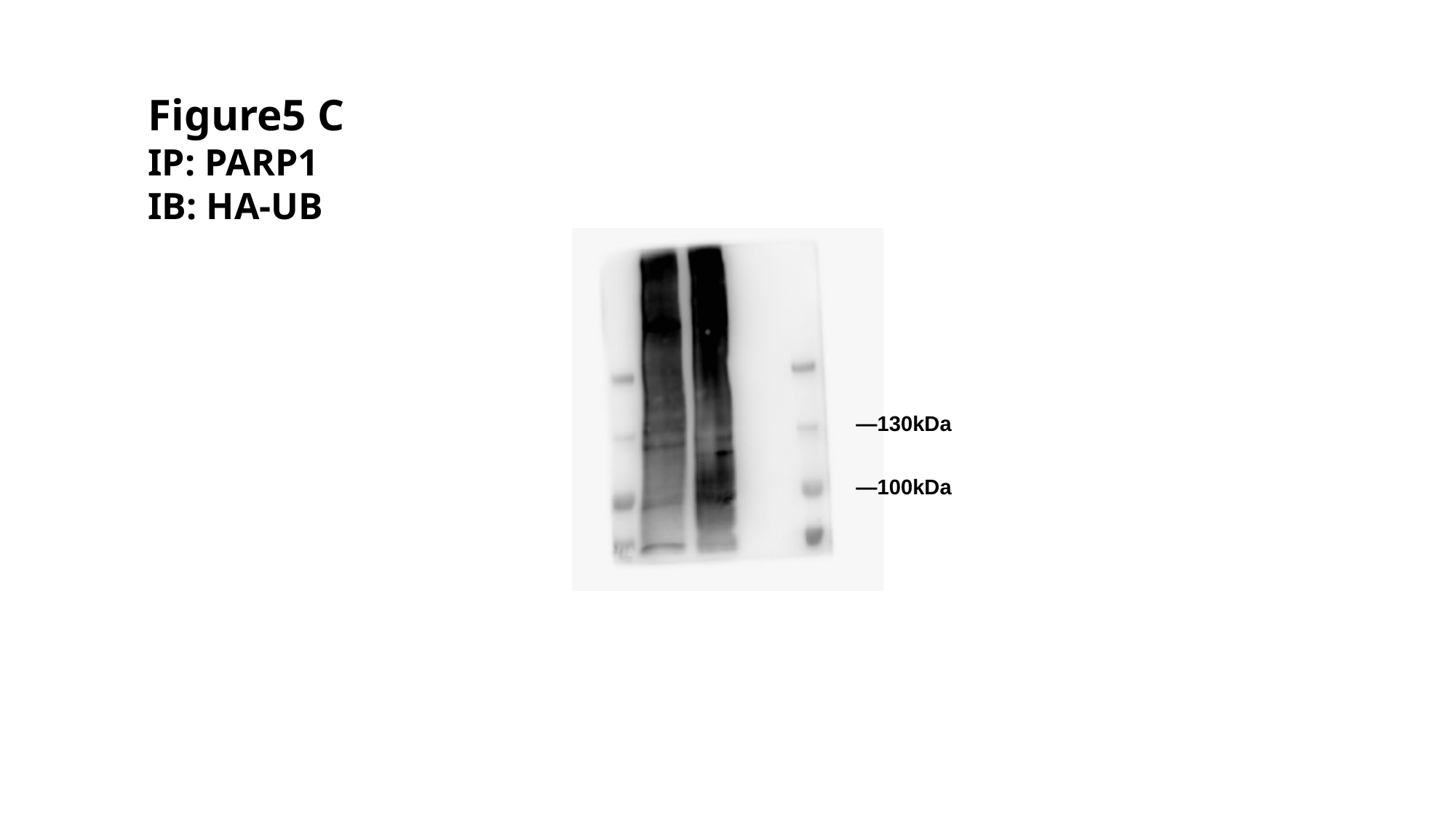

Figure5 C
IP: PARP1
IB: HA-UB
—130kDa
—100kDa

## Slide 67
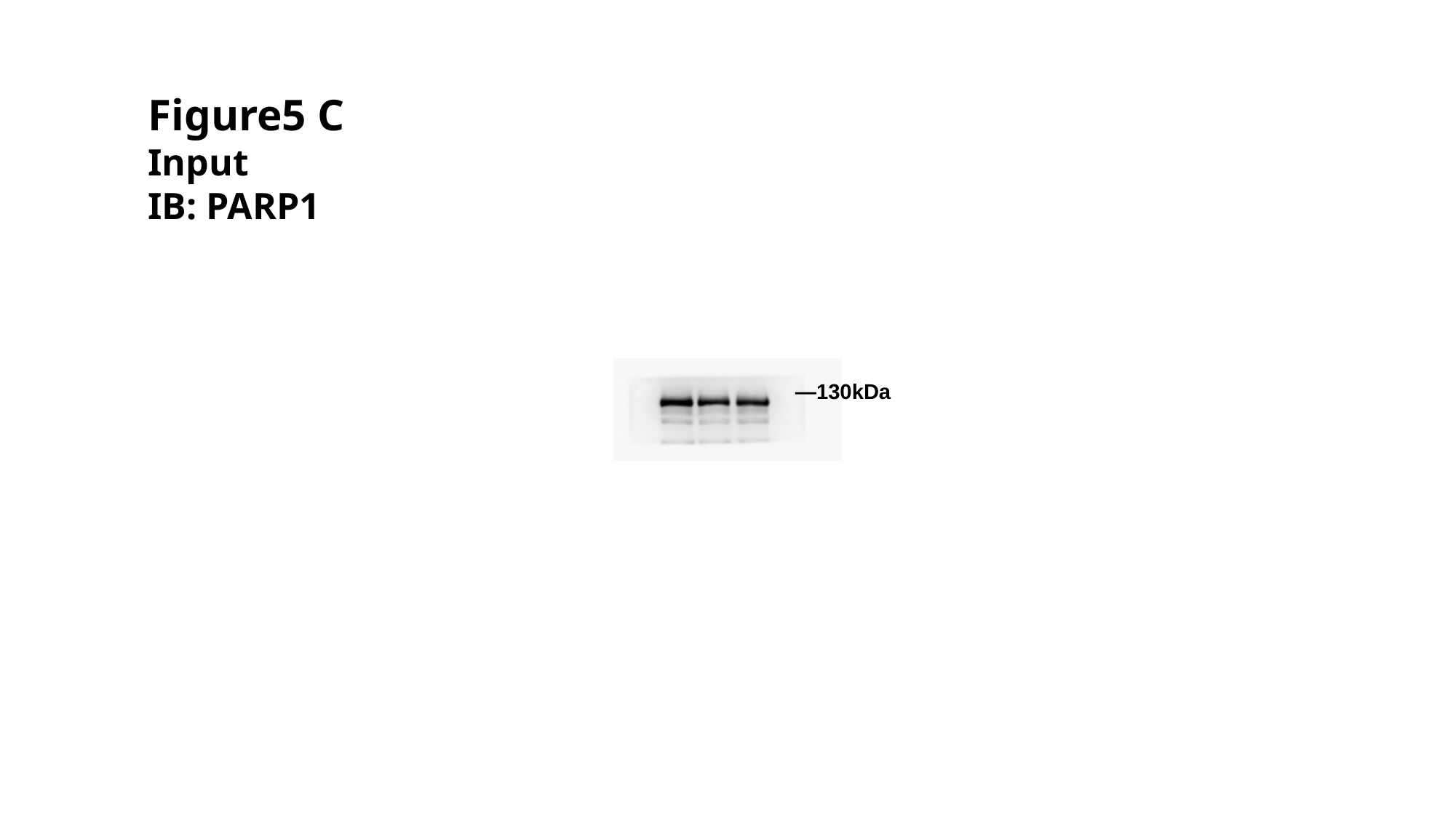

Figure5 C
Input
IB: PARP1
—130kDa

## Slide 68
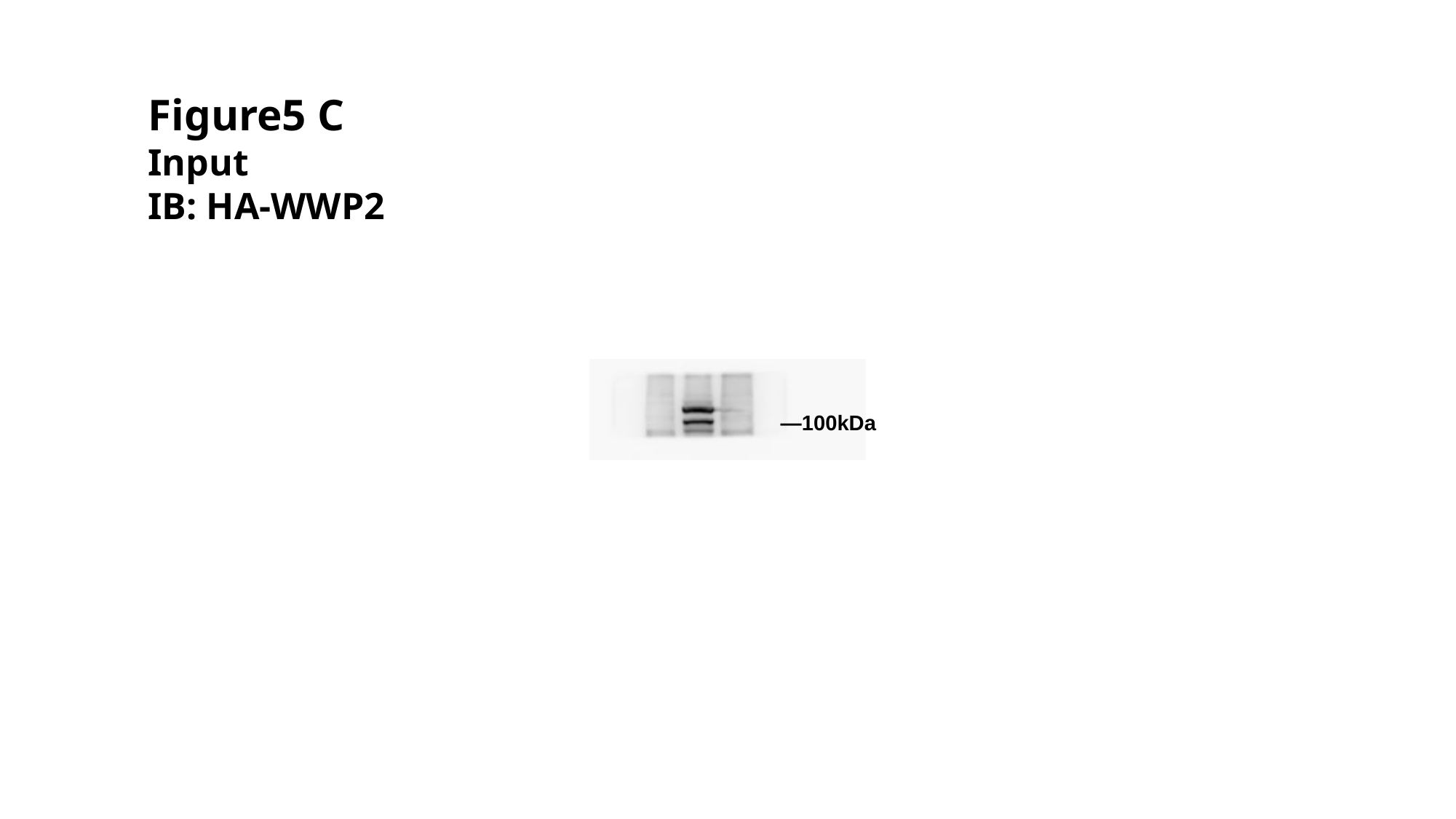

Figure5 C
Input
IB: HA-WWP2
—100kDa

## Slide 69
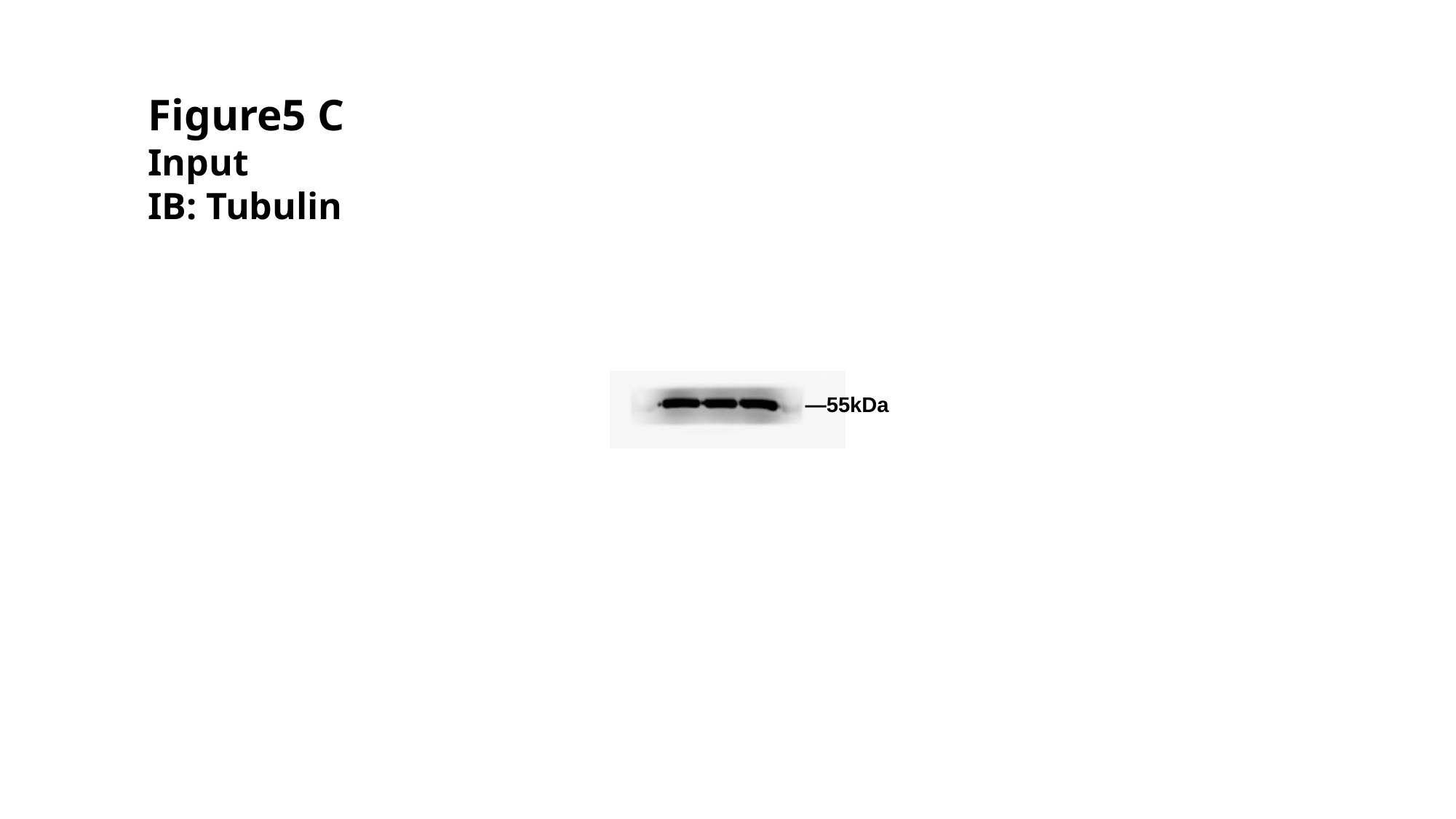

Figure5 C
Input
IB: Tubulin
—55kDa

## Slide 70
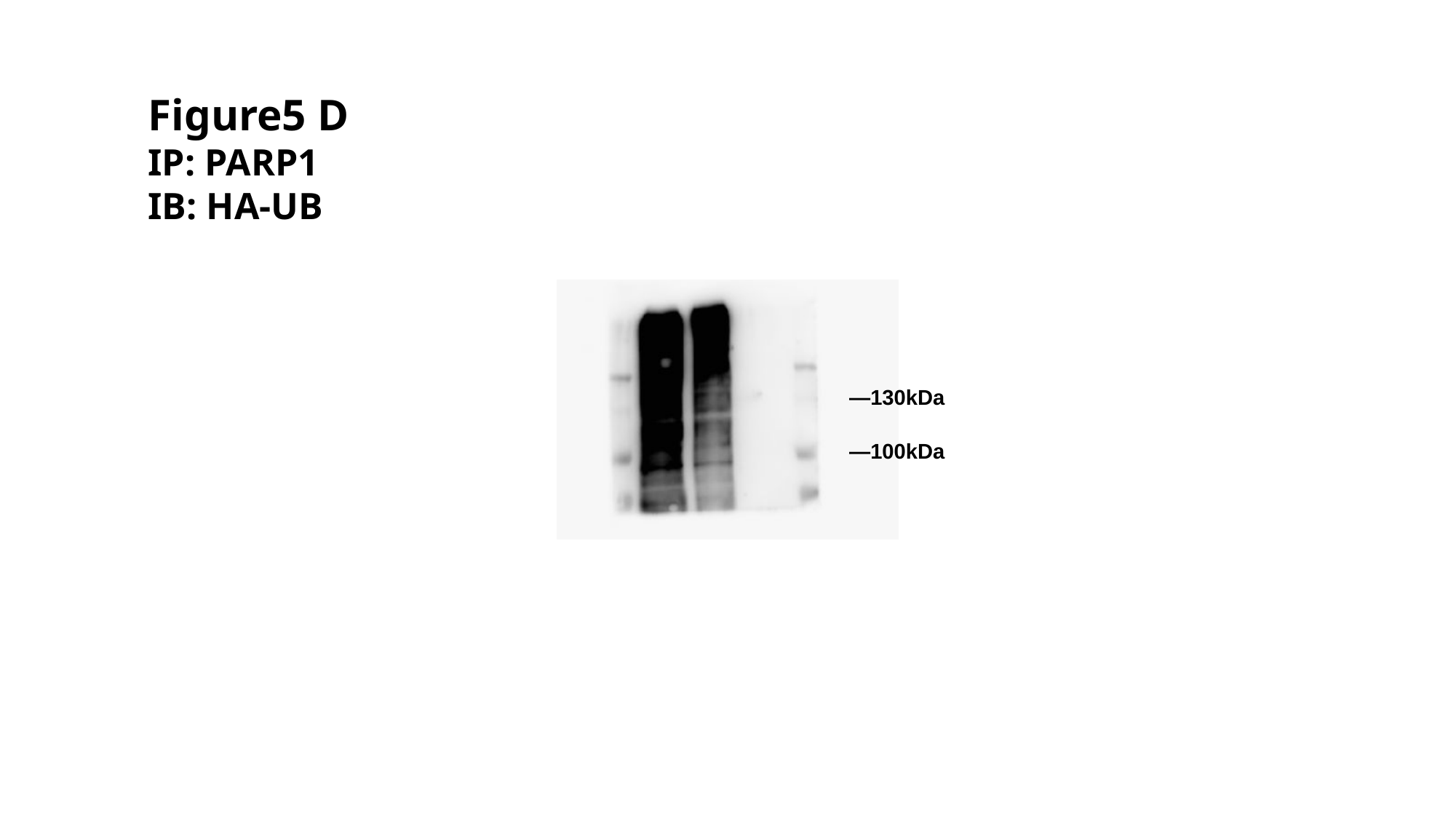

Figure5 D
IP: PARP1
IB: HA-UB
—130kDa
—100kDa

## Slide 71
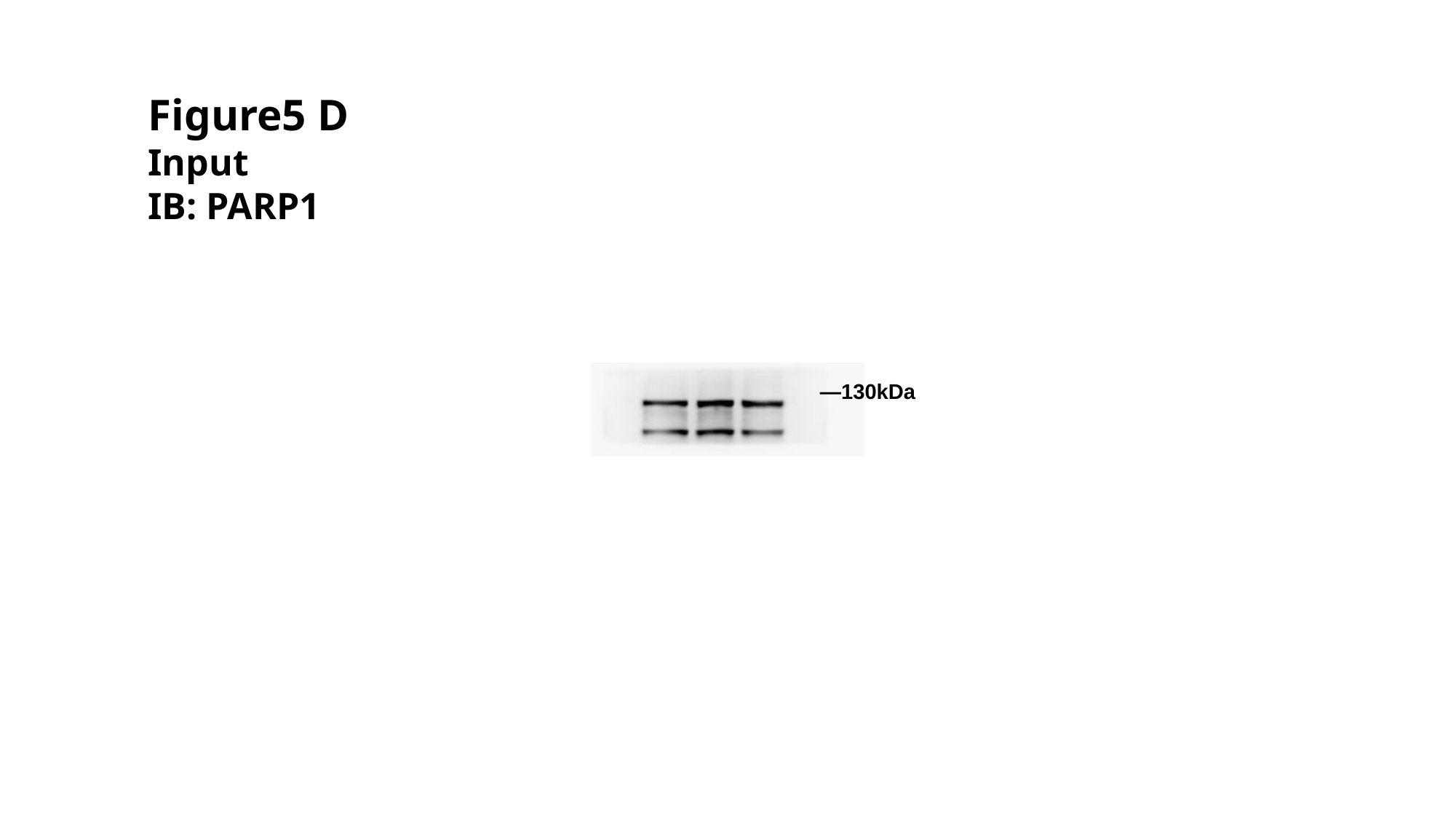

Figure5 D
Input
IB: PARP1
—130kDa

## Slide 72
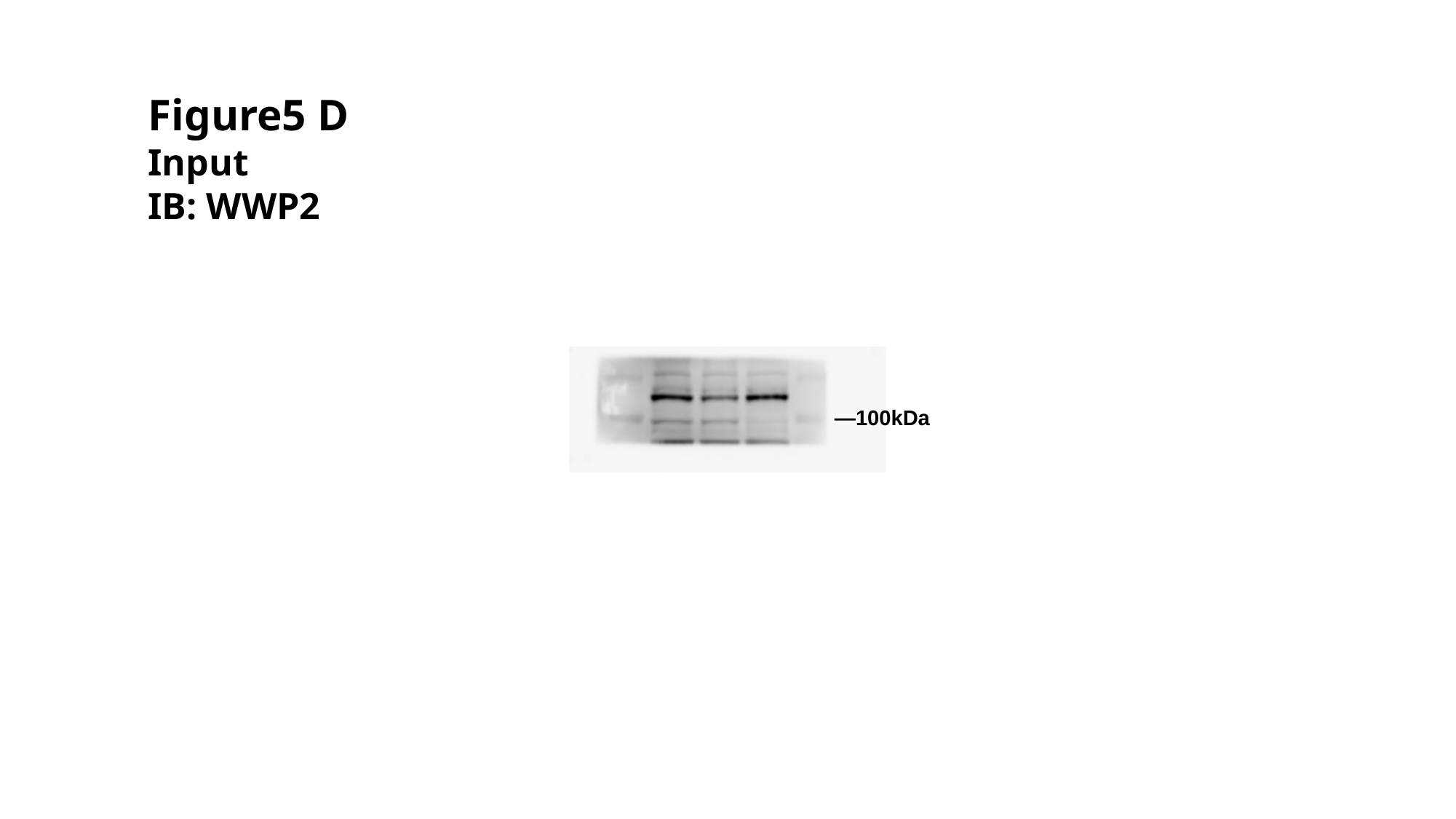

Figure5 D
Input
IB: WWP2
—100kDa

## Slide 73
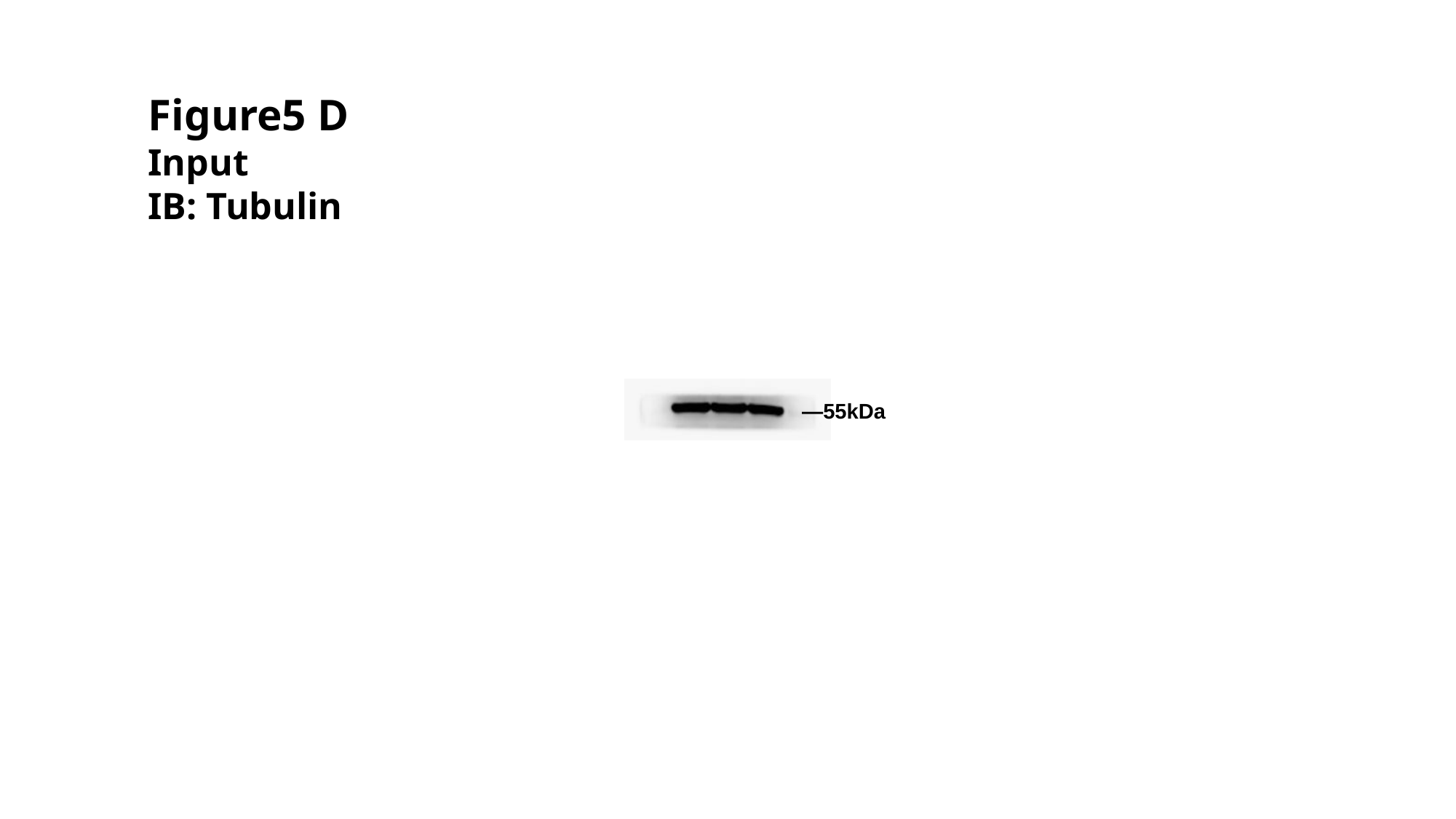

Figure5 D
Input
IB: Tubulin
—55kDa
